# Qi-Blood Harmony Formula mediates post-MI cardioprotection via FTO-driven dual modulation of energy metabolism and fibrosis: a multi-omics validation

**DOI:** 10.1186/s13020-026-01506-w

**Published:** 2026-11-02

**Authors:** Xuena Xie, Yuying Wang, Xiaonan Dong, Rong Yuan, Yu Miao, Pengqi Li, Zhitao Wang, Caizhen Zhu, Han Zhao, Lantian Hu, Mei Liu, Zihan Li, Meiwen Huang, Shudong Ma, Qiqi Xin, Weihong Cong, Keji Chen

**Affiliations:** 1 School of Pharmacy, Faculty of Medicine, Macau University of Science and Technology, Macau, 999078 SAR China; 2 Laboratory of Cardiovascular Diseases, Xiyuan Hospital, China Academy of Chinese Medical Sciences, Beijing, 100091 China; 3 Fuwai Hospital, National Center for Cardiovascular Diseases, Chinese Academy of Medical Sciences and Peking Union Medical College, Beijing, 100037 China; 4 Tianjin University of Traditional Chinese Medicine, Tianjin, 301617 China

**Keywords:** Traditional Chinese medicine, Qi-Blood Harmony Formula, Myocardial infarction, FTO, Multi-omics, Fibrosis, Energy metabolism

## Abstract

Myocardial infarction (MI) remains a leading cause of death worldwide. Persistent myocardial fibrosis and dysregulated energy metabolism following MI contribute to adverse cardiac remodeling and are associated with poor clinical prognosis. Currently, no guideline-recommended therapy provides effective long-term intervention simultaneously targeting both of these core pathological mechanisms. The Qi-Blood Harmony Formula (QBHF), a classical Chinese herbal prescription, has demonstrated cardioprotective potential on attenuating fibrosis, preserving mitochondrial integrity, and restoring myocardial ATP levels in MI, yet its underlying mechanisms and therapeutic targets remain unclear. Using an integrated multi-omics approach in a mouse MI model, we showed that QBHF reactivated genes related to energy metabolism while suppressing pro-fibrotic pathways, as revealed by bulk RNA-sequencing. Non-targeted metabolomics further confirmed its efficacy in counteracting metabolic dysregulation. Single-nucleus RNA sequencing revealed that QBHF altered cardiac cellular composition characterized by reducing a major profibrotic fibroblast subpopulation and expanding a metabolically robust cardiomyocyte subset. Integrative multi-omics analysis identified the fat mass and obesity-associated protein (FTO) as a central regulatory hub modulated by QBHF across bulk heart tissue, fibroblasts, and cardiomyocytes. Crucially, heterozygous knockout of FTO in mice abolished QBHF-mediated improvements in cardiac fibrosis, ATP level and heart function. Consistently, FTO knockdown in cardiomyocytes blunted QBHF-induced ATP restoration. Together, these findings demonstrate that QBHF confers post-MI cardioprotection via FTO-driven dual modulation of energy metabolism and fibrosis. Our findings highlight FTO as a promising therapeutic target and provide novel mechanistic insights into traditional Chinese medicine-based interventions for post-MI injury.

**Supplementary Information:**

The online version contains supplementary material available at https://doi.org/10.1186/s13020-026-01506-w.

## Introduction

Myocardial infarction (MI) is the most severe manifestation of ischemic heart disease and remains the leading cause of global morbidity and mortality [1]. Characterized by a complex pathogenesis, MI demands both immediate acute intervention and long-term prognostic management. For acute-phase treatment, despite significant advances in coronary artery bypass grafting and percutaneous coronary intervention, nearly one-third of patients are ineligible for revascularization due to underlying coronary conditions [2]. Moreover, among those who undergo intervention, approximately 30% experience poor clinical outcomes attributable to coronary microvascular “no-reflow” phenomena [3–7]. When patients fail to derive adequate benefit from rapid ischemia-relieving interventions, a downstream cascade of pathological changes ensues. These changes include energetic metabolism disorders, inflammatory infiltration, cardiomyocyte death, fibrosis, and adverse structural remodeling. Ultimately, this cascade leads to irreversible heart failure [8–10]. However, current therapeutic regimens fail to counteract the long-term structural and functional deterioration driven by molecular dysregulation [11, 12], underscoring the need for interventions that concurrently target multiple pathways [13]. Effective amelioration of these pathological processes is pivotal for limiting myocardial injury after MI. Previous studies have shown that optimizing cardiac energy metabolism following infarction reduces the incidence of angina and all-cause mortality in MI patients [14] and lowers the risk of death on day 5 by 39% [15]. Attenuating myocardial fibrosis also improves ventricular remodeling and decreases cardiac death and heart failure events [16, 17]. Indeed, existing therapeutic options aimed at post-MI pathological progression remain limited: most confer only single-dimension benefits and can even impose mutually conflicting strategies. To date, no approved drug carries guideline-level indications for the long-term modulation of cardiac energy and myocardial fibrosis in MI [18, 19]. Besides, apart from mortality, new-onset heart failure exerts the greatest impactful outcome on long-term quality of life and readmission among MI survivors. The 2022 AHA/ACC/HFSA Guideline for the Management of Heart Failure designates the improvement of myocardial metabolism and fibrosis as key future research directions for pharmacotherapy [20]. In short, identifying interventions that concurrently rectify post-MI metabolic dysfunction and myocardial fibrosis is essential to address the unmet clinical needs of patients with current therapies. Such interventions hold broad clinical promise.

Dysregulation of epitranscriptomic mechanisms, particularly *N*6-methyladenosine (m^6^A) RNA modifications, has been increasingly recognized as a critical determinant of post-injury cardiac responses [21, 22]. Fat mass and obesity-associated protein (FTO), an essential eraser of m^6^A modifications, has emerged as a pivotal regulator of metabolic disorders, including cardiovascular disease. Studies of adipose tissue further implicate FTO in the control of adipogenesis and systemic energy homeostasis [23, 24]. Its primary function is to erase m^6^A marks in RNA. This demethylation activity is essential for regulating mRNA stability, processing, and translational efficiency, thereby underscoring FTO’s relevance in adipogenesis and metabolic disorders [25, 26]. In cardiac tissue, FTO governs the stability and translation of pivotal transcripts. Key targets include sarcoplasmic/endoplasmic reticulum calcium ATPase 2a (SERCA2a) and peroxisome proliferator-activated receptor-gamma coactivator-1alpha (PGC1α). Through regulating these targets, FTO modulates calcium handling and mitochondrial biogenesis [27, 28]. Importantly, reduced FTO expression under pathological stress impairs myocardial contractile function, amplifies energetic deficits, and exacerbates interstitial fibrosis [29, 30]. These findings highlight FTO’s potential as a therapeutic target for cardiac repair.

Traditional Chinese medicine (TCM) represents a promising complementary strategy to conventional regimens for cardiovascular protection by simultaneously modulating multiple biological pathways central to MI pathophysiology [31, 32]. The cardioprotective properties of TCM formulations stem from their multi-target nature, enabling them to regulate a broad spectrum of processes, including epigenetic modifications, inflammatory responses, fibrotic progression, maladaptive remodeling, apoptosis, calcium handling, and endothelial function [33–37]. The Qi-Blood Harmony Formula (QBHF) is a four-herb Chinese formulation derived from the classical “Qi-Invigorating and Blood-Activating” therapeutic strategy in TCM. It is composed of four herbs: Huang Qi (*Astragali Radix*), Dan Shen (*Salviae Miltiorrhizae Radix et Rhizoma*), Chuan Xiong (*Chuanxiong Rhizoma*), and Chi Shao (*Paeoniae Radix Rubra*). This formula is derived from the classic Anti-myocardial Infarction Mixture (KXGHJ). KXGHJ was developed by Academician Chen Keji at Xiyuan Hospital in the 1970s and has been used for AMI management for over 50 years. Evidence supports its efficacy in alleviating symptoms, improving cardiac function, and reducing complications [38]. Historically used throughout China, these four herbs in QBHF constitute the core of classical TCM prescriptions and typical Chinese patent medicines, such as the Modified Buyang Huanwu Decoction (recorded in *Yi Lin Gai Cuo*) and Naoxintong capsules [39, 40]. These formulations are used for treating ischemic heart diseases, including coronary heart disease (CHD) and acute coronary syndromes, with the aims of promoting blood circulation, resolving blood stasis, and tonifying Qi [41–43]. Building upon this established traditional knowledge and clinical legacy, QBHF was modified to investigate the synergistic effects of its concise composition in a standardized manner. According to TCM theory, within the QBHF, Huang Qi (*Astragali Radix*) tonifies the Qi of the spleen and lung, thereby promoting blood circulation. Meanwhile, Dan Shen (*Salviae Miltiorrhizae Radix et Rhizoma*), Chuan Xiong (*Chuanxiong Rhizoma*), and Chi Shao (*Paeoniae Radix Rubra*) collaborate to promote Qi circulation, activate blood, cool the blood, and resolve stasis. The synergistic action of these herbs collectively achieves the effects of tonifying Qi, activating blood circulation, unblocking the vessels, and eliminating stasis. This combination of actions is deliberately designed to target the TCM syndrome of “Qi deficiency and blood stasis”. In contemporary TCM clinical practice, this syndrome is widely recognized as a central pathological pattern in patients during the recovery phase of MI, typically manifesting as fatigue, shortness of breath, and lingering chest pain [43, 44]. Moreover, the same four herbs contain multiple bioactive constituents with demonstrated cardioprotective properties. Key compounds include tetramethylpyrazine, astragaloside IV, paeoniflorin, salvianolic acids A/B, and tanshinone. These constituents independently protect against MI by improving myocardial contractility and energy metabolism, reducing fibrosis and oxidative stress, enhancing mitochondrial function, and suppressing inflammation [45–50]. Thus, QBHF is not only grounded in classical theory but also reflects a continuously applied treatment paradigm in modern TCM cardiology.

Despite its promising pharmacological profile, the multi-component nature of QBHF presents challenges for dissecting precise mechanisms of action through conventional pharmacological approaches. To bridge this gap, it is necessary to identify the pathways underlying QBHF’s cardioprotective effects in MI. While traditional bulk transcriptomics averages gene expression across heterogeneous cardiac cell populations, single-nucleus RNA sequencing (snRNA-seq) provides a higher-resolution approach to analyze transcriptional profiles at the level of individual cells [51, 52]. This technique allows identification of cell-type-specific gene-expression signatures and their alterations in response to TCM interventions [53–55]. Integrating snRNA-seq with bulk transcriptomic and non-targeted metabolomics data creates a multidimensional framework that can help unravel the complex mechanisms behind TCM efficacy [56, 57].

Against this background, we hypothesized that QBHF exerts a multi-target cardioprotective effect by regulating FTO to synergistically improve cardiac energy metabolism and suppress fibrosis following MI. By integrating pharmacodynamic assessments in a mouse MI model with multi-omics analyses, including bulk RNA sequencing, non-targeted metabolomics, and snRNA-seq, we examined how QBHF enhances cardiac energy metabolism and attenuates fibrosis after MI injury. Our findings further identify FTO, an m^6^A RNA demethylase, as a key mediator of these effects. Together, these results provide new insights into the multi-target actions of QBHF. They bridge traditional medicine with contemporary molecular cardiology and advance therapeutic strategies for ischemic heart disease and related conditions.

## Methods and materials

### Reagents and chemicals

RIPA lysis buffer (P0013B), enhanced BCA protein assay kit (P0009), cell counting kit-8 (CCK-8, C0037), enhanced ATP assay kit (S0027), protease and phosphatase inhibitor cocktail (P1045), and phenylmethanesulfonyl fluoride (PMSF, ST506) were obtained from Shanghai Beyotime Biotechnology Co., Ltd. (Shanghai, China). Primary antibodies targeting FTO and GAPDH for western blotting were obtained from Proteintech Group, Inc. (Wuhan, China). The horseradish peroxidase (HRP)-conjugated secondary antibody and AffiniPure goat anti-rabbit IgG (H+L) were acquired from Huaxingbio Gene Technology Co., Ltd. (Beijing, China). 2,3,5-Triphenyltetrazolium chloride (TTC) staining solution, 4% formaldehyde solution and 8% PAGE Color (Green) Gel Ultra Rapid Preparation Kit were obtained from Servicebio Technology Co., Ltd. (Wuhan, China). Phosphate-buffered saline (PBS) was purchased from WISSEN Co., Ltd. (Beijing, China). Polyvinylidene fluoride (PVDF, ISEQ00010) membrane was purchased from Millipore (Bedford, USA). Tribromoethanol, used as an anesthetic for mice, was purchased from Beijing Jitian Biotechnology Co., Ltd. (Beijing, China). Isoflurane was sourced from Lunan Beit Pharmaceutical Co., Ltd. (Shandong, China). The Evo M-MLV One Step RT-qPCR Kit was provided by Accurate Biology (Hunan, China). A mouse creatine kinase-MB isoenzyme (CK-MB) ELISA kit was acquired from Elabscience Biotechnology Co., Ltd. (Wuhan, China). Acetonitrile (LC–MS grade) was procured from Fisher Scientific (Loughborough, UK). Formic acid was supplied by TCI (Shanghai, China), and ammonium formate was obtained from Sigma-Aldrich (Shanghai, China). Ultrapure water was prepared using a Milli-Q purification system (Millipore, Bedford, USA). ATP quantitative analysis kit (ALS22033), assay medium (ALS22111), and supplement substrate (ALS22121) were bought from Alicelligent (Beijing, China). Dulbecco's Modified Eagle’s Medium/nutrient mixture F-12 (DMEM/F-12) medium (PM150312), DMEM/F-12 sugar-free basal medium (PM150333), and AC16 Cell complete medium (CM-0790) were purchased from Pricella Biotechnology Co., Ltd. (Wuhan, China). Fetal bovine serum (10099141C) and penicillin–streptomycin were purchased from Gibco (Carlsbad, CA, USA).

### Preparation of QBHF extract

All herbs were purchased from Hebei Baicaokang Pharmaceutical Co., Ltd. (Hebei, China) (Table 1). QBHF was prepared from *Astragali Radix* (Huangqi), *Salviae Miltiorrhizae Radix et Rhizoma* (Danshen, batch number), *Chuanxiong Rhizoma* (Chuanxiong), and *Paeoniae Rubra Radix* (Chishao) (ratio 2:2:1:1). To ensure consistency, a single bulk extraction was performed under Good Manufacturing Practice (GMP) guidelines by the Pharmacy Department of Xiyuan Hospital, China Academy of Chinese Medical Sciences. Briefly, 1000 g of the authenticated herbal mixture was immersed in distilled water (8 mL/g) for 30 min, followed by reflux extraction for 1.5 h and immediate hot filtration. The residue was extracted again with distilled water (6× volume, mL/g) under reflux for 1 h and filtered. The combined filtrates were concentrated under reduced pressure to a soft extract with a density of approximately 2 g/mL (expressed as crude drug equivalent) and stored at − 80 °C until use. The composition was analyzed by ultra-high-performance liquid chromatography-mass spectrometry (UHPLC-MS). Detailed methodologies are described in the Supplemental methods, and the results are presented in Supplementary Tables 1–3, and Fig. S1E-F).

**Table 1:** Detailed information of herbs in QBHF

Chinese name	Source	TCM name	Part(s) used	Amount (g)	Batch number
Huangqi	*Astragalus membranaceus* (Fisch.) Bge	*Astragali Radix*	root	30	C22032051
Danshen	*Salvia miltiorrhiza* Bunge	*Salviae Miltiorrhizae Radix et Rhizoma*	root and rhizome	30	C22090516
Chuanxiong	*Ligusticum chuanxiong* Hort	*Chuanxiong Rhizoma*	rhizome	15	C22032107
Chishao	*Paeonia lactiflora* Pall	*Paeoniae Radix Rubra*	root	15	C22032418

### Experimental animals

Eight-week-old male C57BL/6J mice (25 ± 5 g) were purchased from Vital River Laboratory Animal Technology Co., Ltd. (Beijing, China). Male FTO-KO C57BL/6J mice (25 ± 5 g, heterozygous FTO^+/−^) and FTO wild-type (WT) mice (25 ± 5 g) were provided by GemPharmatech Co., Ltd. (Nanjing, China).

All procedures were carried out at the Experimental Animal Center of Xiyuan Hospital, China Academy of Chinese Medical Sciences (Beijing, China) under controlled conditions (20–25 °C, 55–65% relative humidity, 12 h light/12 h dark cycle) and in accordance with the ARRIVE guidelines. Protocols were approved by the Institutional Animal Ethics Committee of Xiyuan Hospital (Approval No. 2023XLC008-3).

### Mouse model of left anterior descending coronary artery (LAD) ligation-induced MI

The MI model was induced through permanent ligation of the LAD in all the model and intervention groups [58]. Mice were anesthetized with intraperitoneal tribromoethanol (1.25%, 0.1 mL/10 g), intubated, and ventilated with a small-animal respirator (Model 55-7040, Harvard Apparatus; tidal volume 0.2 mL, 120 breaths/min). After routine skin preparation, a left thoracotomy was performed through the third and fourth intercostal space to expose the heart. The LAD was ligated with a 6-0 silk suture. The intercostal muscles and ribs were closed with 5-0 Vicryl, leaving a small opening for pleural decompression, and the skin was sutured with 4-0 nylon. The sham-operated mice underwent the same procedure without LAD ligation. Post-surgery, mice were administered intraperitoneal sodium penicillin (80,000 U/day) for three consecutive days to prevent infection.

### Animal grouping and intervention

For the *in vivo* efficacy experiments of QBHF in post-MI C57BL/6J mice, the animals were randomly assigned to six groups: the sham group, MI group, low-dose QBHF group (MI + QBHF 5.85 g/kg/day), medium-dose QBHF group (MI + QBHF 11.70 g/kg/day), high-dose QBHF group (MI + QBHF 23.40 g/kg/day), and positive group (MI + Perindopril tert-butylamine 0.52 mg/kg/day + metoprolol tartrate 13 mg/kg/day). At the same time, for multi-omics research, myocardial samples from the MI and medium-dose QBHF groups were used to perform non-targeted metabolomics analysis, and samples from the sham, MI, and medium-dose QBHF groups were preserved for bulk RNA-seq and snRNA-seq.

For mechanism-of-action studies, WT C57BL/6J mice and FTO heterozygous knockout (FTO^+/-^) mice were used. WT C57BL/6J mice were randomly allocated to WT sham, WT MI, and WT QBHF (MI + QBHF 11.70 g/kg/day) groups; FTO^+/-^ mice were allocated to FTO^+/-^ sham, FTO^+/-^ MI, and FTO^+/-^ QBHF (MI + QBHF 11.70 g/kg/day) groups.

QBHF dosage (5.85, 11.70, or 23.40 g/kg/day) was scaled from the adult clinical dose (90 g/day for a 70 kg patient), with the equivalent mouse dose calculated according to body surface area conversion. Dose conversion according to body surface area: animal dose (mg/kg) = human clinical dose (mg/kg) × human body surface area conversion coefficient / animal body surface area conversion coefficient, with a conversion factor of 9.1. The clinical dosage for adult use was 90 g per day, which corresponds to an intragastric dosage for the middle-dose group: 90 × 9.1 / 70 ≈ 11.7 g/kg/day [59]. The low, middle, and high dose groups were administered QBHF at a ratio of 1:2:4, corresponding to doses of 5.85 g/kg/day, 11.70 g/kg/day, and 23.40 g/kg/day, respectively. The positive controls were treated with a combination of perindopril tert-butylamine (0.52 mg/kg/day, ACE inhibitor) and metoprolol tartrate (13 mg/kg/day, β-blocker) in accordance with standard therapeutic protocols. The sham-operated and model mice received an equivalent volume of saline (0.9% NaCl, sterile). Mice were pretreated for three consecutive days before surgery, followed by intervention for 7 days post-MI in multi-omics studies and 21 days for *in vivo* efficacy experiments and mechanism-of-action studies.

### TTC staining

TTC staining was used to quantify infarct size at 24 h post-MI. Hearts were collected immediately after euthanasia and carefully rinsed with ice-cold PBS to eliminate remaining blood and subsequently sliced into 5 sections approximately 2 mm thick. The slices were incubated in 1% TTC at 37 °C for 30 min, then fixed in 4% paraformaldehyde and imaged. The infarct size was calculated as the ratio of unstained (infarcted) to stained (viable) area [60].

### Echocardiography

Twenty-one days after surgery, mice were lightly anesthetized with isoflurane (1–2%), intubated, and placed supine on an electrical heating pad at 37 °C. Transthoracic echocardiography was performed using a Vevo 2100 ultrasound system (Visualsonics, Toronto, Canada) as previously described. Continuous ECG was recorded via limb electrodes. Parasternal long-axis M-mode images were acquired at a heart rate ≥ 450 beats per minute [61]. Left ventricular ejection fraction (LVEF), LV fractional shortening (LVFS), LV end-diastolic internal diameter (LVIDd), LV end-systolic internal diameter (LVIDs), LV end-diastolic volume (LVVold), and LV end-systolic volume (LVVols) were averaged from three consecutive cardiac cycles.

### Measurement of creatine kinase MB activities

Plasma levels of creatine kinase-MB (CK-MB) were quantified using an ELISA kit following the manufacturer’s protocol.

### Heart weight to tibia length ratio

Mice were euthanized, and their hearts were excised and weighed. The heart weight-to-tibia length (HW/TL) ratio was then calculated [62].

### Pathological staining

Hematoxylin and eosin (H&E) staining was performed following the manufacturer’s instructions (Servicebio, Wuhan, China). Histopathological alterations were examined using Caseviewer software (Servicebio, Wuhan, China).

Masson’ s trichrome staining was performed following the standard operating procedures provided by Servicebio Technology Co., Ltd to assess myocardial fibrosis. Stained sections were scanned, and histological features were examined using Caseviewer software. Fibrotic areas were quantitatively analyzed with ImageJ software (National Institutes of Health, USA).

### ATP levels measurement

Myocardial ATP content was determined with the Enhanced ATP Assay Kit following the manufacturer’s instructions [63]. ATP levels were quantified with a multi-mode microplate reader (EPOCH, Thermo).

### Transmission electron microscopy

Morphological evaluation by transmission electron microscopy (TEM) was carried out following Servicebio Technology standardized protocols. After being prepared with a Leica UC7 ultramicrotome, ultrathin sections were imaged at 80 kV immediately under a HITACHI HT7800 electron microscope [64]. Mitochondrial ultrastructure was quantified from TEM images with ImageJ, focusing on density, size, and cristae organization [65].

### Bulk RNA-seq

Cardiac tissues were harvested seven days after surgery. Total RNA was extracted, and ribosomal RNA was depleted during library construction to enrich mRNA. Sequencing libraries were prepared following standard strand-specific protocols and sequenced on the Illumina HiSeq3000 platform. Raw sequencing reads were trimmed with Cutadapt (v4.1) to remove adapters and low-quality bases [66]. Clean reads were aligned to the mouse reference genome (GRCm38/mm10) using STAR (v2.7.9a) [67], and gene expression levels were quantified with featureCounts (v2.0) [68]. Differential expressions were analyzed using DESeq2 [69]. Genes with an absolute fold change (|FC|) ≥2 and a *P*‑value < 0.05 were considered differentially expressed [70]. Gene Ontology (GO) and Kyoto Encyclopedia of Genes and Genomes (KEGG) pathway enrichment analyses were conducted using clusterProfiler [71]. Gene Set Enrichment Analysis (GSEA) was also performed on the full ranked gene list [72]. Additionally, gene set variation analysis (GSVA) was conducted to assess pathway activity at the sample level by using the GSVA package [73].

### Non-targeted metabolomics by liquid chromatography-tandem mass spectrometry

Seven days post-operation, cardiac tissue was collected for non-targeted metabolomics. Metabolite profiling was performed on a Vanquish UHPLC system coupled with an Orbitrap Exploris 120 mass spectrometer [74]. Separation was achieved on an ACQUITY UPLC® HSS T3 column (150 × 2.1 mm, 1.8 μm) at 40 °C using positive and negative electrospray ionization [75]. LC and MS parameters are detailed in Supplementary Table 4.

Mass spectrometric data were acquired in full MS‑ddMS^2^ mode converted to mzXML format, and processed using XCMS for feature extraction, retention-time alignment, and peak matching [76, 77]. Metabolites were identified by accurate mass (mass error < 30 ppm) and MS/MS spectra against public databases including HMDB [78], MassBank [79], LipidMaps [80], mzCloud [81], and KEGG [82].

Signal drift was corrected by quality control‑based LOESS. Features with RSD > 30% in QC samples were excluded [83]. Multivariate analysis was carried out with the ropls package in R [84]. Differential metabolites were selected based on variable importance in projection (VIP) > 1 and *P* < 0.05 [85]. Pathways enrichment analysis was analyzed with MetaboAnalyst [86].

### SnRNA-seq

Seven days after operation, left-ventricular tissues were collected for snRNA-seq. Single nuclei were isolated, and libraries were prepared using the 10× Genomics Chromium platform and sequenced on an Illumina HiSeq 2500 (Illumina, USA). Raw reads were processed with CellRanger for alignment, barcode assignment, and UMI counting [87]. Adapters and low-quality reads were trimmed with Cutadapt (v1.17) [88] and aligned to the mouse reference genome (GRCm38) with STAR [67], and gene expression counts were generated using featureCounts [68]. Downstream analysis was conducted using the Seurat package (v3.1.2) in R [89]. Cells expressing fewer than 200 or more than 7000 genes, or with mitochondrial gene content exceeding 5%, were excluded. After quality control, gene expression data were normalized with NormalizeData and scaled with ScaleData [90]. The 2000 most variable features were identified with FindVariableFeatures [91]. Principal component analysis (PCA) and the top 20 principal components were selected for subsequent clustering with the FindClusters algorithm [92]. Cellular heterogeneity was visualized with uniform manifold approximation and projection (UMAP) using RunUMAP. Cell types were annotated based on established marker genes and cross-validated with the SingleR database [93]. Differential expression was determined using FindAllMarkers (min.pct = 0.25) and FindMarkers (Wilcoxon rank-sum test, adjusted *P* < 0.05).

Functional enrichment analysis of GO terms and KEGG pathways was conducted with clusterProfiler (v3.10.1) [71]. Pseudotime trajectory analysis was performed using Monocle2 (v2.24.0) [94]. Pathway activity scores were calculated with AUCell and Ucell [95, 96], and metabolic activity was assessed with scMetabolism [97].

### Cell culture

AC16 human cardiomyocyte cell line was obtained from the Chinese Academy of Sciences Cell Bank (Shanghai, China) and maintained in DMEM/F-12 supplemented with 10% fetal bovine serum (FBS) and 1% penicillin–streptomycin at 37 °C and 5% CO₂ [98].

### Preparation of QBHF-containing serum

Sixty male Wistar rats (200 ± 20 g) were purchased from HFK Bio-Technology Co., Ltd. (Beijing, China) and randomly allocated to five groups: (a) control group (n = 20), (b) low-dose QBHF group (4.05 g/kg/day, n = 10), (c) middle-dose QBHF group (8.10 g/kg/day, n = 10), (d) high-dose QBHF group (16.20 g/kg/day, n = 10), and (e) positive group (Perindopril tert-butylamine 7.48 mg/kg/day + metoprolol tartrate 18.91 mg/kg/day, n = 10). QBHF or saline (0.9% NaCl, sterile) was administered intragastrically once daily for five consecutive days at a constant gavage volume. Two hours after the final administration, rats were anaesthetized, subjected to abdominal aortic blood collection, and subsequently euthanized [99]. Serum was separated by incubating the blood at 37 °C for 30 min and then centrifuging it at 3000 rpm, 4 °C for 15 min. The supernatant was filtered through 0.22 μm membrane filter and stored at − 80 °C. The constituents of QBHF-containing serum were identified by UHPLC‑MS. Detailed methods are provided in the Supplemental Methods, and the results are listed in Supplementary Tables 5‑6 and Fig. S1E, F.

### Oxygen and glucose deprivation (OGD)

OGD was carried out as described previously [100]. In brief, the cells were transferred to glucose-free DMEM/F-12 supplemented with 10% FBS and incubated for 12 h at 37 °C in anaerobic pouches (MGC, Japan) for a duration of 12 h. Normoxic controls were maintained in complete DMEM/F-12 (3151 mg/L D-Glucose) under 95% air and 5% CO_2_ at 37 °C.

### Cell transfection

Lentiviral vectors for FTO overexpression (OE) and knockdown (KD) were generated by Genechem Co., Ltd. (Shanghai, China). Following the manufacturer’s protocol, AC16 cells were infected at a multiplicity of infection (MOI) of 10 (OE) or 20 (KD) for 12 h, after which the medium was replaced with complete DMEM/F-12 and cells were cultured for an additional 60 h under standard conditions (95% air / 5% CO_2_, 37 °C). GFP expression was examined under a fluorescence inverted microscope (Leica, Germany) after 72 h, prior to further experimental analysis [101]. OE or KD efficiency was verified by RT-qPCR.

### QBHF-containing serum treatment

AC16 cells were seeded in 96-well plates (5 × 10^4^ cells/100 μL/well) and 6-well plates (2 × 10^5^ cells/1 mL/well) and then cultured overnight. After the medium was removed, cells were incubated with QBHF-containing serum for 12 h and then subjected to hypoxia for another 12 h. Controls remained in complete DMEM/F-12 at 37 °C, 95% air, 5% CO₂ [102].

### Cell viability

Cell viability was detected using the CCK-8 assay [103]. AC16 cells were seeded in 96-well plates and incubated overnight to adhere. Cells in treatment groups received QBHF-containing or positive drug-containing serum for 12 h prior to OGD. Cells maintained under normoxic conditions served as a control. Controls and models received control serum. After treatment, cell viability was evaluated with CCK-8 reagent (diluted in DMEM/F-12), and absorbance was read using a microplate reader at 450 nm (BioTek, Agilent Technologies, USA).

### Cellular ATP production rate

ATP production rates in AC16 cells were assessed using a Seahorse XF96 Analyzer (Agilent Technologies, USA) as previously reported [104]. AC16 cells were seeded in Seahorse XF96 microplates (Agilent Technologies, USA), equilibrated in Seahorse Base Medium, and incubated for 1 h at 37 °C in a CO_2-_free chamber.

For the XF ATP Rate Assay (Alicelligent, China), oligomycin (1.5 μmol/L), antimycin A (1.0 μmol/L), and rotenone (1.0 μmol/L) were injected sequentially to monitor real-time changes in the oxygen consumption rate (OCR) and extracellular acidification rate (ECAR). Data acquisition and analysis were conducted using Seahorse Wave Pro software (Agilent Technologies, USA), and key metabolic parameters and plots were generated via Seahorse Analytics. Values were normalized to total protein content for accurate comparison [105].

### Quantitative real-time PCR

Total RNA was extracted with the MagaBio Plus Total RNA Purification Kit II and quantified using the Evo M-MLV One Step RT-qPCR Kit (SYBR) on an Archimed R6 Real-Time PCR System. Reverse transcription and amplification were performed in a one-step procedure. Gene expression was calculated by the 2^(−ΔΔCT)^ method and normalized to *β-actin*. Primer sequences are listed in Supplementary Table 7.

### Western blot

Protein samples for western blot were prepared with ice-cold lysis in RIPA buffer containing protease/phosphatase inhibitors and 1 mM PMSF. After centrifugation, protein concentrations in the supernatants were determined using an Enhanced BCA assay. Equal amounts of protein were resolved on 8% SDS–polyacrylamide gels and electrotransferred to PVDF membranes. Membranes were blocked, incubated with primary antibodies and corresponding HRP-conjugated secondary antibodies, and visualized using sensitive or ultrasensitive chemiluminescent substrates. Band intensities were quantified using SWE Image Gray Analysis Software (Servicebio, Wuhan, China) and normalized to loading control.

### Statistical analysis

The data were presented as the mean ± standard error of the mean (SEM), and the corresponding sample sizes (n) were provided in the figure legends. Statistical analysis was performed using R software (v 4.4.3) [106] and GraphPad Prism 9.5 (GraphPad Software, Inc., IL, USA). Comparisons between two independent groups were examined using two-tailed Student’s t-test. Differences among multiple groups with a single independent variable were evaluated using analysis of variance (ANOVA). The threshold for statistical significance was set at *P* < 0.05.

## Results

### QBHF ameliorates cardiac function and alleviates myocardial remodeling in mice with LAD-induced MI

**Fig. 1:**
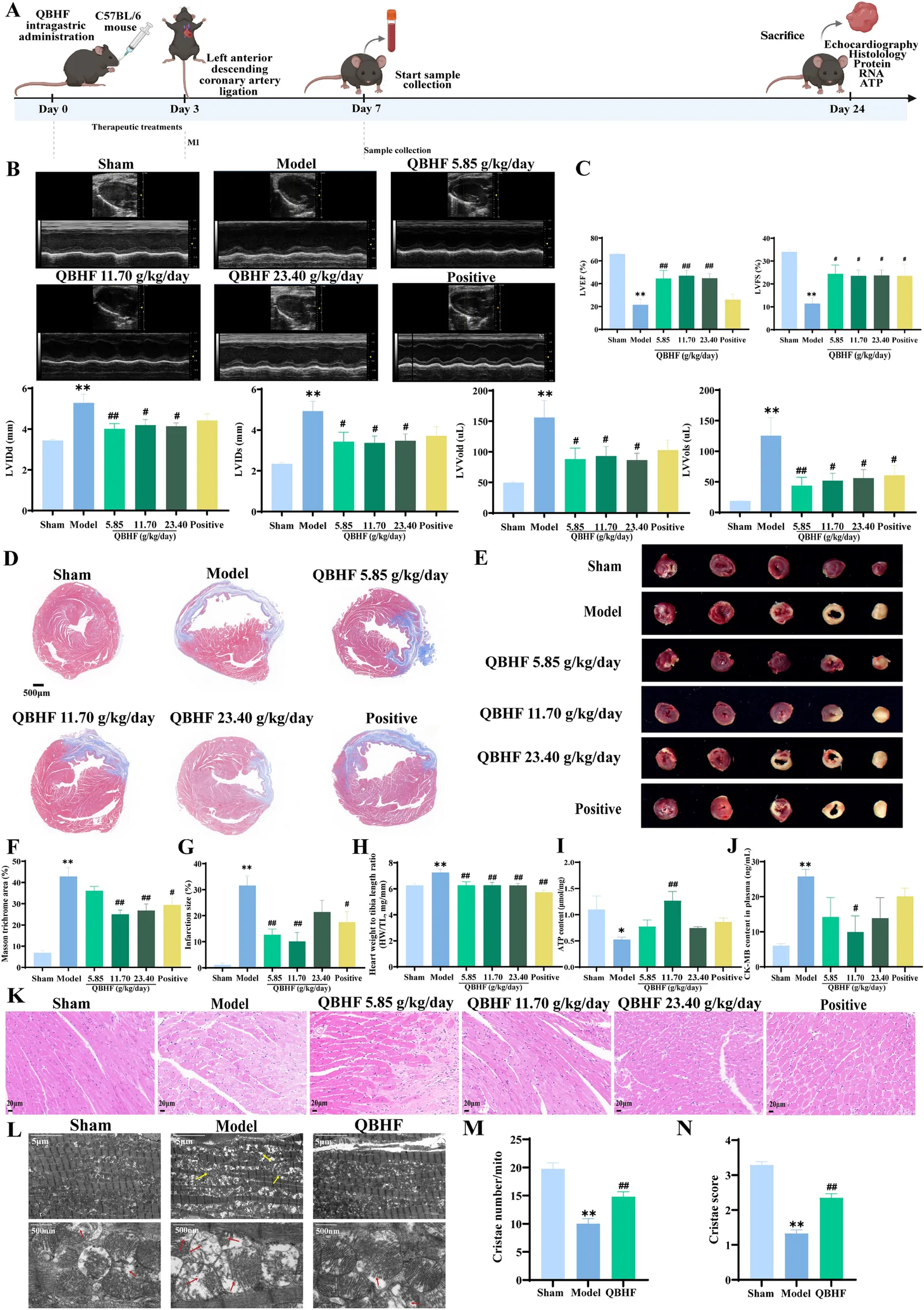
Protective effects of QBHF on cardiac function and structure in MI mice. **A** Schematic illustration of the experimental workflow. **B** Representative M-mode traces and **C** statistical evaluation of LVEF, LVFS, LVIDd, LVIDs, LVVold, and LVVols after 4-week intervention (n ≥ 6). **D** Representative images of Masson’s trichrome staining. **E** Representative TTC staining image. **F** Quantification of fibrosis (n = 6). **G** Quantification of infarct size (n = 6). **H** HW/TL ratio (n ≥ 6). **I** Myocardial ATP levels (n = 5). **J** Serum CK-MB levels by ELISA in sham, model, QBHF group (n = 6). **K** Representative H&E of the myocardial tissues (n = 6). **L** Representative TEM images of sham, model, and QBHF group hearts (n = 3). Red arrows indicate the missing cristae and vacuole inside the mitochondrion. Yellow arrows indicate the faded and disarrayed sarcomeric structure. **M** Mitochondrial cristae count and **N** cristae integrity score (> 50 mitochondria from 3 mice per group). Data are presented as the mean ± SEM. *P < 0.05, **P < 0.01 vs. sham group; ^#^*P* < 0.05, ^##^*P* < 0.01 vs. model group

Based on a LAD-induced MI mouse model, the protective effects of QBHF on cardiac function were assessed (Fig. 1A). After LAD ligation, echocardiography revealed that LVEF and LVFS were markedly reduced, and LVIDd, LVIDs, LVVold, and LVVols were markedly increased in MI mice compared to sham-operated controls (Fig. 1B, C). However, compared with the MI mice, LVEF and LVFS were markedly increased, and LVIDd, LVIDs, LVVold, LVVols, and HW/TL ratio were decreased in the QBHF group (Fig. 1C and H).

TTC staining showed a markedly enlarged infarct size in MI hearts compared with sham. This enlargement was substantially attenuated by treatment with QBHF at 5.85 or 11.70 g/kg/day, and by the positive drug control. Notably, the middle dose of QBHF (11.70 g/kg/day) produced the greatest reduction in infarct size (Fig. 1E–G) and significantly lowered the elevated plasma levels of the myocardial injury biomarker CK-MB (Fig. 1J).

H&E staining of cardiomyocytes exhibited well-preserved myocardium in the sham hearts, with orderly cell arrangement, intact morphology, and uniformly distributed nuclei (Fig. 1K). In contrast, the MI hearts showed severe myocardial necrosis, extensive inflammatory infiltration, and fibrosis, whereas both QBHF and the positive drug reduced inflammation and fibrosis, and preserved myocardial tissue integrity (Fig. 1K). Additionally, Masson’s trichrome staining confirmed a marked increase in collagen content in model hearts (Fig. 1D and F). Notably, QBHF significantly decreased collagen deposition, especially at the 11.70 g/kg/day dose (Fig. 1D and F).

TEM further revealed mitochondrial injury in the model hearts, including a reduction in mitochondrial density, irregular ultrastructure, disrupted cristae, vacuolation, and disorganized myofibrils compared to sham. In the QBHF group, however, the mitochondrial structure remained relatively intact (Fig. 1L–N). Furthermore, QBHF significantly restored the MI-depleted myocardial ATP content (Fig. 1I), indicating its beneficial effect on cardiac energy metabolism (Fig. 1I).

In summary, these findings demonstrate that QBHF not only mitigates cardiac dysfunction and adverse remodeling following LAD-induced MI, but also supports metabolic recovery by preserving mitochondrial integrity and restoring bioenergetic capacity.

### Bulk transcriptomic analysis reveals QBHF-induced reversal of metabolic dysfunction and alleviation of myocardial fibrosis after MI

**Fig. 2:**
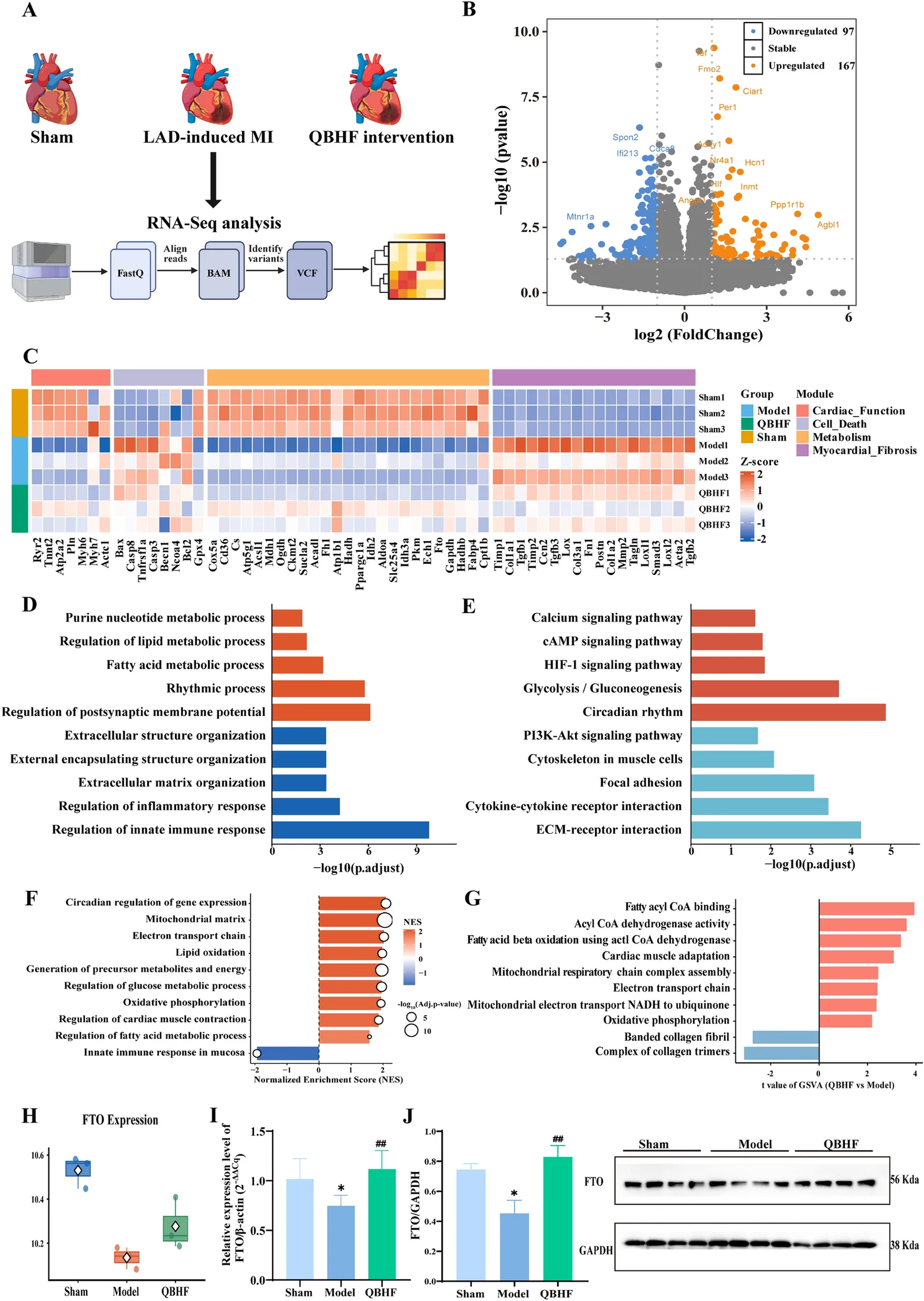
Transcriptomic profiling reveals QBHF-induced reversal of disrupted metabolic and fibrotic programs in the post-MI heart. **A** Schematic workflow of the bulk RNA-sequencing experiment. **B** Volcano plot of DEGs between QBHF‑treated and model mice (criteria: |log₂FC|> 1, adjusted *P* < 0.05); significantly up‑regulated and down‑regulated genes are shown in orange and blue, respectively. **C** Heatmap of DEGs in sham, model, and QBHF groups. **D** GO terms for significantly enriched biological processes. **E** Significantly enriched KEGG pathways. **F** Significantly enriched GSEA pathways in MI model and QBHF-treated mice. **G** GSVA pathway analysis across groups. **H** FTO expression levels from bulk RNA‑seq data (n = 3). **I** RT‑qPCR validation of FTO mRNA levels (n = 6). **J** Western blot analysis of FTO protein expression. Representative images show FTO levels in sham, model, and QBHF groups, with GAPDH as the loading control (n = 4). Data are presented as the mean ± SEM. *P < 0.05 vs. sham group; ^##^*P* < 0.01 vs. model group

To elucidate the cardioprotective mechanisms of QBHF, bulk RNA sequencing was performed (Fig. 2A). Differential expression analysis between the QBHF-treated and model groups identified 167 up-regulated and 97 down-regulated genes (Fig. 2B). Heatmap and unsupervised clustering of DEGs revealed a key gene set markedly dysregulated in MI hearts that is linked to cardiac function, cardiomyocyte survival, metabolism, and fibrosis. Conversely, QBHF restored the expression of these genes to sham-like levels (Fig. 2C). GO enrichment showed that up-regulated genes were primarily associated with purine nucleotide metabolism, lipid metabolism, fatty acid metabolism, and circadian rhythm-related processes, whereas down-regulated genes were enriched in extracellular structure/matrix organization, inflammatory, and innate immune responses (Fig. 2D). Consistently, KEGG pathway analysis confirmed that QBHF up-regulated pathways related to calcium, cAMP, glycolysis/gluconeogenesis, HIF-1, and circadian rhythm signaling, while suppressing those involved in focal adhesion, PI3K-Akt, cytoskeletal regulation, cytokine-cytokine receptor interaction, and extracellular matrix (ECM)-receptor interaction pathways (Fig. 2E). GSEA demonstrated that QBHF positively regulated oxidative phosphorylation, fatty acid metabolism, cardiac muscle contraction, and electron transport chain pathways (Fig. 2F), and GSVA revealed enhanced activity of oxidative phosphorylation and fatty acid metabolism pathways, accompanied by decreased activity of collagen formation-related pathways in QBHF-treated hearts (Fig. 2G). Transcriptomic and RT‑qPCR analyses indicated that QBHF increased FTO mRNA expression (Fig. 2H, I), and western blot analysis confirmed a corresponding up-regulation at the protein level (Fig. 2J).

Collectively, these transcriptomic data indicate that QBHF mitigates post-infarction remodeling by restoring metabolic and contractile programs while suppressing fibrotic and inflammatory signaling.

### Non-targeted metabolomics shows that QBHF alleviates energy metabolism disturbances after MI

**Fig. 3:**
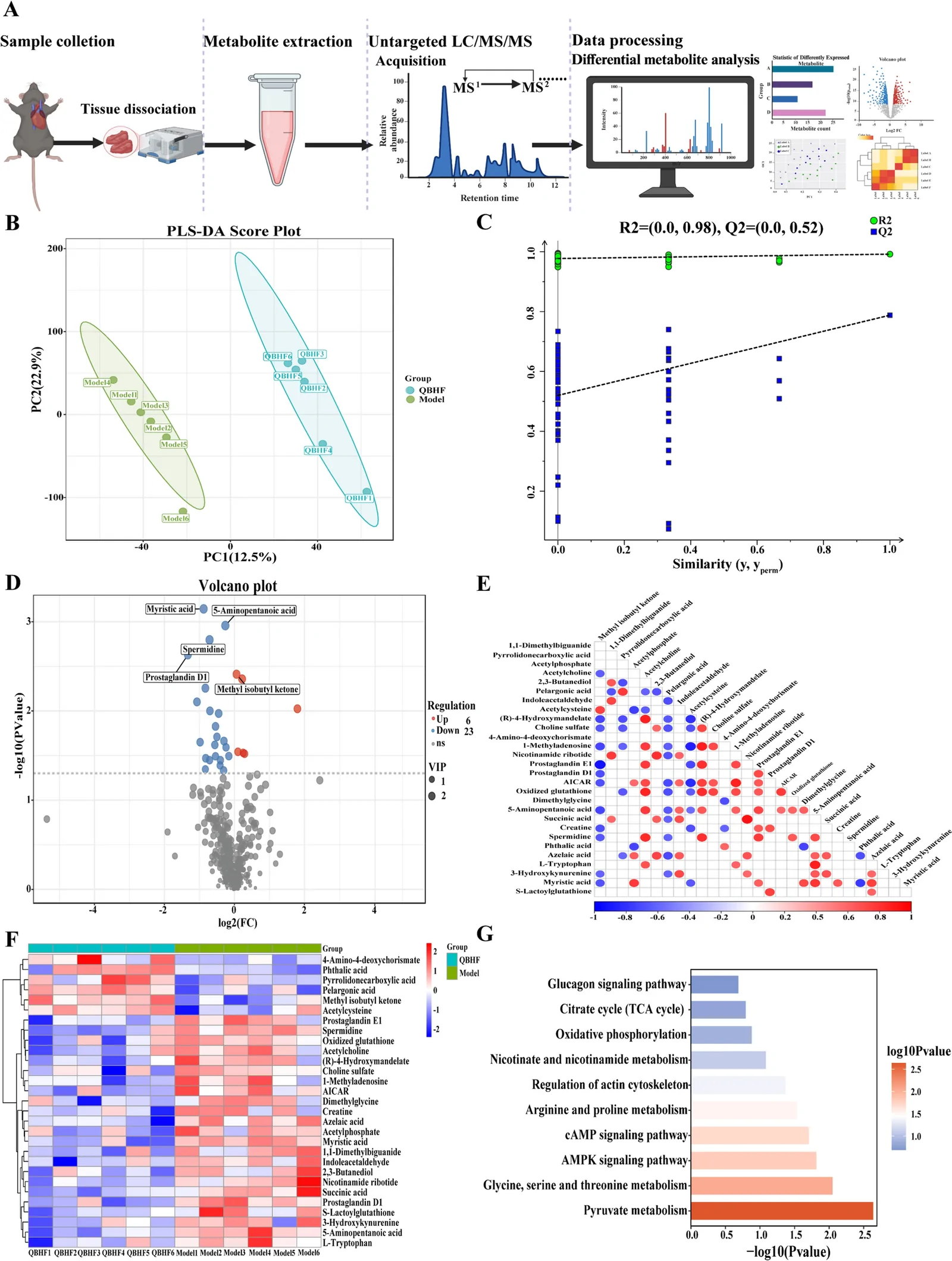
QBHF ameliorates MI by restoring metabolic homeostasis. **A** Experimental workflow for LC–MS‑based non‑targeted metabolomics analysis of myocardial tissue. **B** PLS‑DA score plot. **C** PLS‑DA permutation test plot (200 permutations). **D** Volcano plot of differential metabolites between groups. **E** Correlation heatmap of differential metabolites. **F** Hierarchical clustering heatmap of 29 significantly altered metabolites (red and blue denote increased and decreased levels in the QBHF group compared with the MI group, respectively). **G** KEGG pathway enrichment analysis of differential metabolites; pathways are ranked by − log₁₀(*P*‑value)

Prompted by transcriptomic data, non-targeted metabolomics was performed on peri-infarct tissue to characterize the metabolic effects of QBHF after MI (Fig. 3A). A clear separation was observed in PLS-DA between the MI model and QBHF-treated groups (Fig. 3B). Model validation via a 200-permutation test confirmed the absence of overfitting, as all permuted Q^2^ values remained below the original Q^2^ point on the right, thereby ensuring the reliability of the model (Fig. 3C). Comparative analysis identified 29 differential metabolites (VIP > 1, *P* < 0.05): 6 up-regulated and 23 down-regulated after QBHF intervention, indicating that QBHF helps restore MI-induced metabolic disturbances toward baseline (Fig. 3D–F).

KEGG pathway analysis indicated that QBHF may exert its effects by modulating key metabolic pathways, including pyruvate metabolism, glycine/serine/threonine metabolism, and AMPK signaling, thereby ameliorating energy dysregulation in post‑MI mice (Fig. 3G). Taken together, these findings provide mechanistic insights into the cardioprotective effects of QBHF, revealing that metabolic modulation critically contributes to its therapeutic efficacy against MI.

### SnRNA-seq identifies QBHF-induced benefits: improved cardiac function, restored energy metabolism, and attenuated fibrosis

**Fig. 4:**
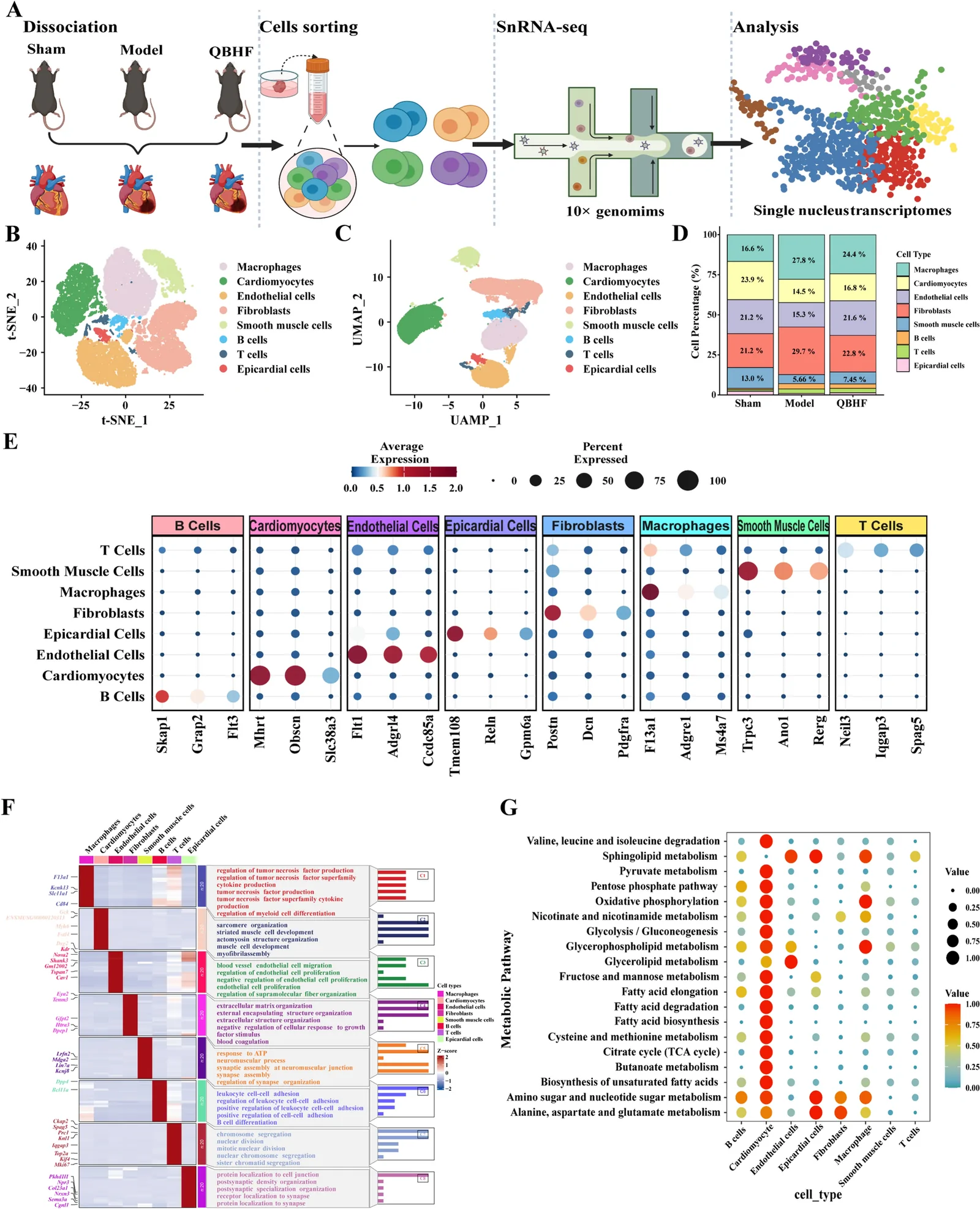
Experimental pipeline and snRNA-seq landscape of QBHF intervention in MI mice. **A** Schematic of the experimental and analytical workflow for snRNA‑seq. **B**, **C** t-SNE (**B**) and UMAP (**C**) visualizations of all sequenced nuclei, colored by major cell type. **D** Bar plot of relative cellular abundance across the sham, model, and QBHF groups. **E** Dot plot of canonical marker-gene expression used to assign cluster identity. **F** Heatmap summarizing sub-cluster annotation, top marker genes per subset, and representative GO terms. **G** Cell-type-specific metabolic pathway enrichment profiles

To elucidate the mechanism underlying the cardioprotective effect of QBHF, peri-infarct tissue was collected from sham, MI, and QBHF-treated groups (Fig. 4A). Single nuclei were isolated using the droplet-based 10× Genomic snRNA-seq. After rigorous quality control filtering, 36,625 high-quality single nuclei were retained for further analysis. Clustering algorithms and dimensionality reduction resolved eight distinct cardiac cell populations, including macrophages, cardiomyocytes, endothelial cells, B cells, fibroblasts, T cells, smooth muscle cells, and epicardial cells. These populations were validate y canonical marker genes and public databases (Fig. 4B, C, E, F). Cell abundance analysis showed MI increased fibroblast and macrophage numbers and decreased cardiomyocyte, endothelial, and smooth muscle cells numbers, while QBHF partially reversed these changes (Fig. 4D). GO enrichment analysis of the cell-type-specific highly expressed genes revealed their corresponding physiological functions (Fig. 4F). Cardiomyocytes displayed the highest metabolic activity among all cell types and were predominantly enriched in metabolic pathways related to nicotinate and nicotinamide, pyruvate, oxidative phosphorylation, fatty acid metabolism, glycolysis/gluconeogenesis, and the citrate cycle (TCA cycle) (Fig. 4G).

**Fig. 5:**
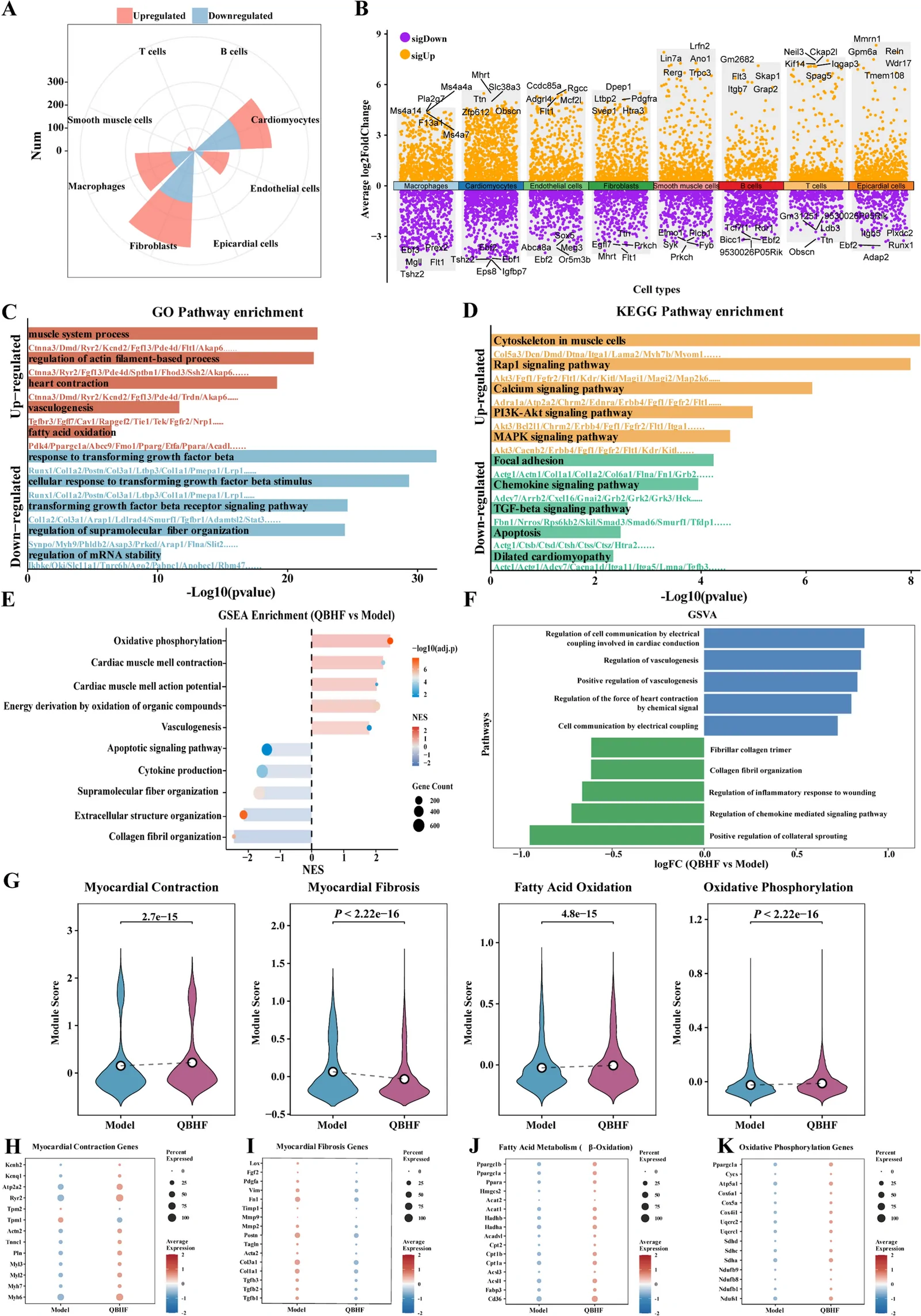
snRNA-seq analysis uncovers the molecular mechanisms of QBHF’s therapeutic effects in MI. **A** Rose chart of DEGs counts between QBHF and MI model groups across major cardiac cell types. **B** Volcano plots of DEGs for each cell type between QBHF and MI model groups. **C**–**F** Enrichment analyses of DEGs: **C** GO terms, **D** KEGG pathways, **E** GSEA of hallmark pathways, and **F** GSVA activity scores. **G** Comparison of pathway activity scores for key biological processes: myocardial contraction, fibrosis, fatty acid oxidation, and oxidative phosphorylation. **H**–**K** Expression of genes linked to **H** cardiac contraction, **I** myocardial fibrosis, **J** fatty acid oxidation, and **K** oxidative phosphorylation in MI and QBHF-treated hearts

To explore the molecular mechanisms driving these cellular changes, differential expression analysis was performed using Seurat’s FindMarkers algorithm to identify DEGs across distinct cell types. Comparison of QBHF-treated versus model groups revealed cell-type-specific transcriptional profiles with notable alterations (Fig. 5A, B). Fibroblasts exhibited the highest number of DEGs, followed by cardiomyocytes. In contrast, T cells, B cells, smooth muscle cells, and epicardial cells showed few or no significant DEGs. These findings highlight the heterogeneous responses of distinct cardiac cell types to QBHF intervention after MI. GO analysis revealed that up-regulated genes were enriched in cardiac contractility, angiogenesis, and fatty acid oxidation, whereas down-regulated genes were associated with TGF-β signaling, fibrotic pathways, and mRNA stability (Fig. 5C). KEGG pathway analysis further indicated up-regulated DEGs mapped to MAPK, calcium, PI3K-Akt signaling, and muscle cytoskeleton organization, while down-regulated DEGs were largely linked to apoptosis, TGF-β, and chemokine signaling (Fig. 5D). GSEA confirmed that the up-regulated pathways in QBHF-treated hearts were related to myocardial contraction, oxidative phosphorylation, and angiogenesis. Conversely, the down-regulated pathways were associated with apoptosis, fibrosis, and extracellular matrix organization (Fig. 5E). GSVA scoring further corroborated these results, showing increased activity in myocardial contraction and angiogenesis pathways, and reduced activity in fibrosis-related pathways in the QBHF group (Fig. 5F). AUCell scoring analysis confirmed these shifts, revealing elevated pathway scores for myocardial contraction, fatty acid oxidation, and oxidative phosphorylation in the QBHF group, with decreased myocardial fibrosis pathway scores (Fig. 5G). Furthermore, QBHF elevated expression of key genes regulating myocardial contraction, fatty acid oxidation, and oxidative phosphorylation, while suppressing that of pro-fibrotic genes (Fig. 5H–L).

In conclusion, snRNA-seq analysis reveals that QBHF exerts cardioprotection through cell-type-specific transcriptional reprogramming, primarily in cardiomyocytes and fibroblasts. This remodeling promotes cardiac functional recovery by enhancing cardiomyocyte contractility and energy metabolism while suppressing maladaptive fibrosis. Thus, QBHF acts as a multi-target therapy that coordinates these key cellular programs to ameliorate post-MI remodeling.

### QBHF modulates fibroblast subpopulations and suppresses cardiac fibrosis in MI injury

**Fig. 6:**
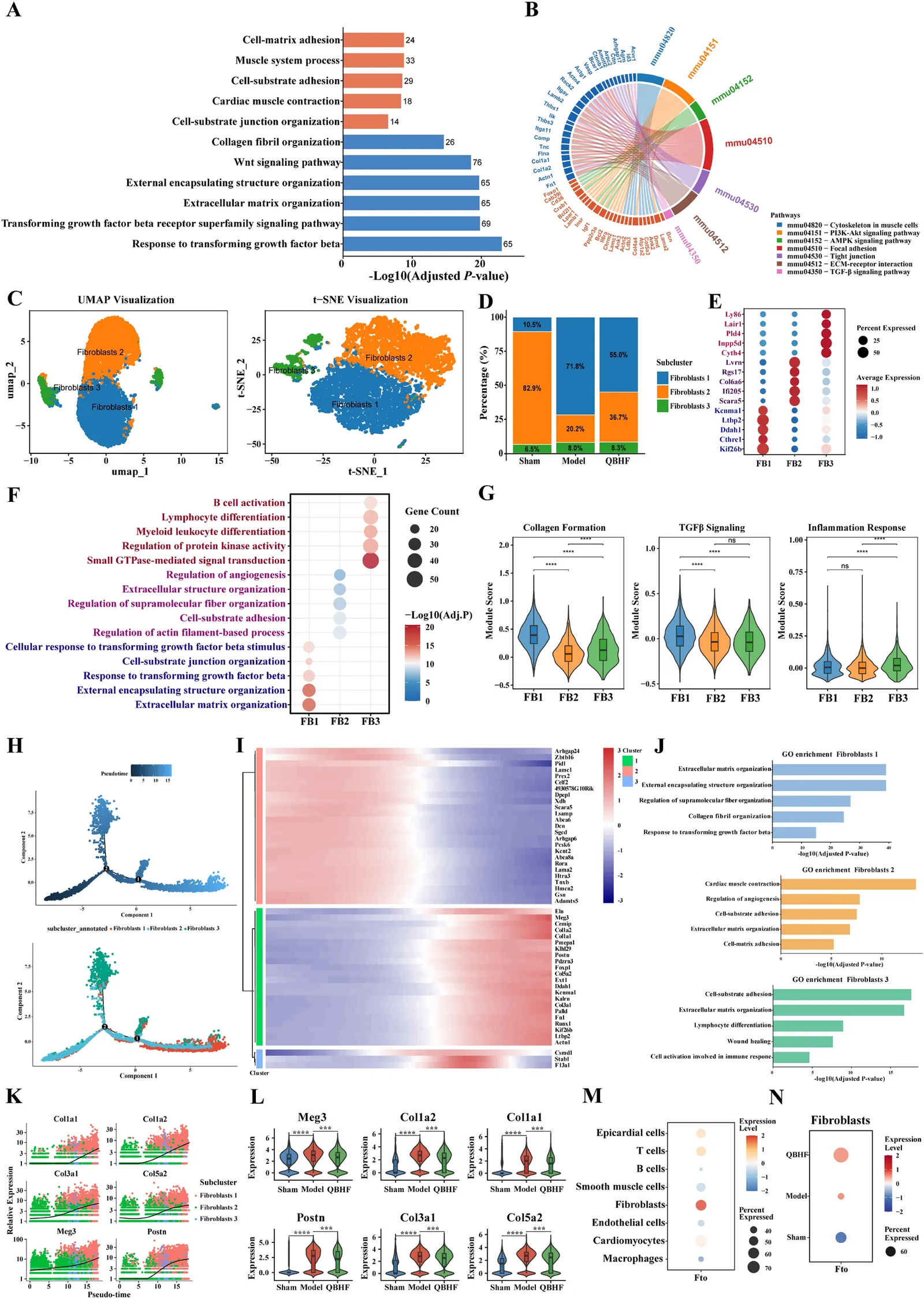
SnRNA-seq analysis demonstrates that QBHF intervention effectively suppresses fibroblast activation and fibrosis. **A** GO enrichment of genes up-regulated or down-regulated by QBHF in fibroblasts. **B** KEGG pathway enrichment of genes regulated by QBHF in fibroblasts. **C** UMAP (left) and t‑SNE (right) visualizations identifying three transcriptionally distinct fibroblast subpopulations. **D** Relative abundance of the fibroblast subpopulations across experimental groups. **E** Dot plots of subpopulation-specific marker genes across the three fibroblast subpopulations. **F** Bubble plots of GO terms enriched in DEGs from fibroblast subpopulations. **G** Functional comparison of collagen formation, TGF-β signaling, and inflammatory response scores among fibroblast subpopulations. **H** Pseudotime trajectory analysis of fibroblasts, colored by pseudotime (top) and subpopulations (bottom). **I** Heatmap of representative gene expression dynamics along the pseudotime trajectory. **J** GO enrichment of DEGs from fibroblast trajectory analysis. **K** Dynamic expression profiles of collagen-related genes (*Col1a1*, *Col1a2*, *Col3a1*, *Col5a2*, *Meg3*, and *Postn*) during the transition period from FB2 to FB1 fibroblasts. **L** Expression levels of the same collagen‑related genes as in **K** across sham, model, and QBHF groups. **M** Bubble plots of FTO expression across cardiac cell types in integrated snRNA-seq dataset. **N** Bubble plots of FTO expression in fibroblasts from sham, model, and QBHF groups

MI injury triggers cascade pathological responses in the heart, where cardiac fibrosis is a primary consequence [107]. The ensuing remodeling process features robust fibroblast activation and proliferation. The subsequent overproduction of extracellular matrix components by specific fibroblast subpopulations leads to tissue disorganization and progressive functional impairment [108, 109]. As previously reported, Masson’s trichrome staining confirmed a marked increase in collagen content in model hearts, validating the presence of myocardial fibrosis (Fig. 1D and F). QBHF reduced fibroblast numbers in the infarcted heart (Fig. 4D) and significantly decreased collagen deposition (Fig. 1D, F). To investigate the mechanism by which QBHF suppresses fibroblast activation and collagen deposition, differential gene expression analysis was conducted on fibroblasts using the snRNA-seq dataset. Functional enrichment revealed that DEGs regulated by QBHF were primarily involved in pathways related to collagen fibril organization, Wingless/Int-1 signaling, extracellular matrix remodeling, and transforming growth factor-β (TGF-β) response (Fig. 6A, B). Three fibroblast subpopulations were resolved (Fig. 6C). Cell abundance analysis showed an expansion of subpopulation 1 (FB1) in the model group, which was significantly reduced following QBHF intervention (Fig. 6D). GO enrichment highlighted distinct functional profiles for the three fibroblast subpopulations, suggesting specialized roles in cardiac homeostasis and injury response (Fig. 6E, F). FB1 was enriched in TGF-β-responsive and fibrosis-related processes, with markers such as *Kif26b*, *Cthrc1*, *Ddah1*, and *Ltbp2* [108, 110, 111], consistent with a profibrotic identity. Subpopulation 2 (FB2) displayed angiogenesis, extracellular matrix production, and structural support functions. It expressed markers including *Scaras*, *Ifi205*, *Col6a6*, *Rgs17*, and *Lvrn*, characteristic of matrix-producing fibroblasts. Subpopulation 3 (FB3) harbored immune-related and inflammatory pathways, with immune-associated genes such as *Inpp5d*, *Pld4*, *Lair1*, and *Ly86* [112], indicating an immunomodulatory fibrocyte-like population. Functional scoring confirmed that among the three fibroblast subpopulations, FB 1 exhibited high levels of collagen-formation and TGF-β-signaling activity, whereas FB 3 displayed elevated inflammatory activity (Fig. 6G). Pseudotime trajectory analysis revealed the clearest transition from quiescent (FB2) to activated fibroblast (FB1), consistent with their distinct roles in matrix production and fibrosis (Fig. 6H–J). FB1 showed high expression of pro-fibrotic genes, including *Col1a1*, *Col1a2*, *Col3a1*, *Col5a2*, *Meg3*, and *Postn* (Fig. 6K), and QBHF significantly down-regulated these fibrosis-related genes (Fig. 6L). To assess the involvement of the m^6^A demethylase FTO in fibroblast activation, FTO expression was visualized across cardiac cell types. SnRNA-seq revealed variable FTO distribution across cardiac cell types, with a notable enrichment in fibroblasts (Fig. 6M). Within this compartment, a lower proportion of FTO‑expressing cells was observed in the model group compared to the sham group. QBHF intervention was associated with a partial restoration of this proportion, implicating that FTO might be involved in mediating QBHF’s effects on fibroblast activation and fibrosis resolution (Fig. 6N). These findings indicated that QBHF mitigates myocardial fibrosis by decreasing the profibrotic fibroblasts fraction, inhibiting fibrotic signaling pathways, and limiting extracellular matrix deposition.

### QBHF confers cardioprotection after MI by modulating cardiomyocyte energy metabolism

**Fig. 7:**
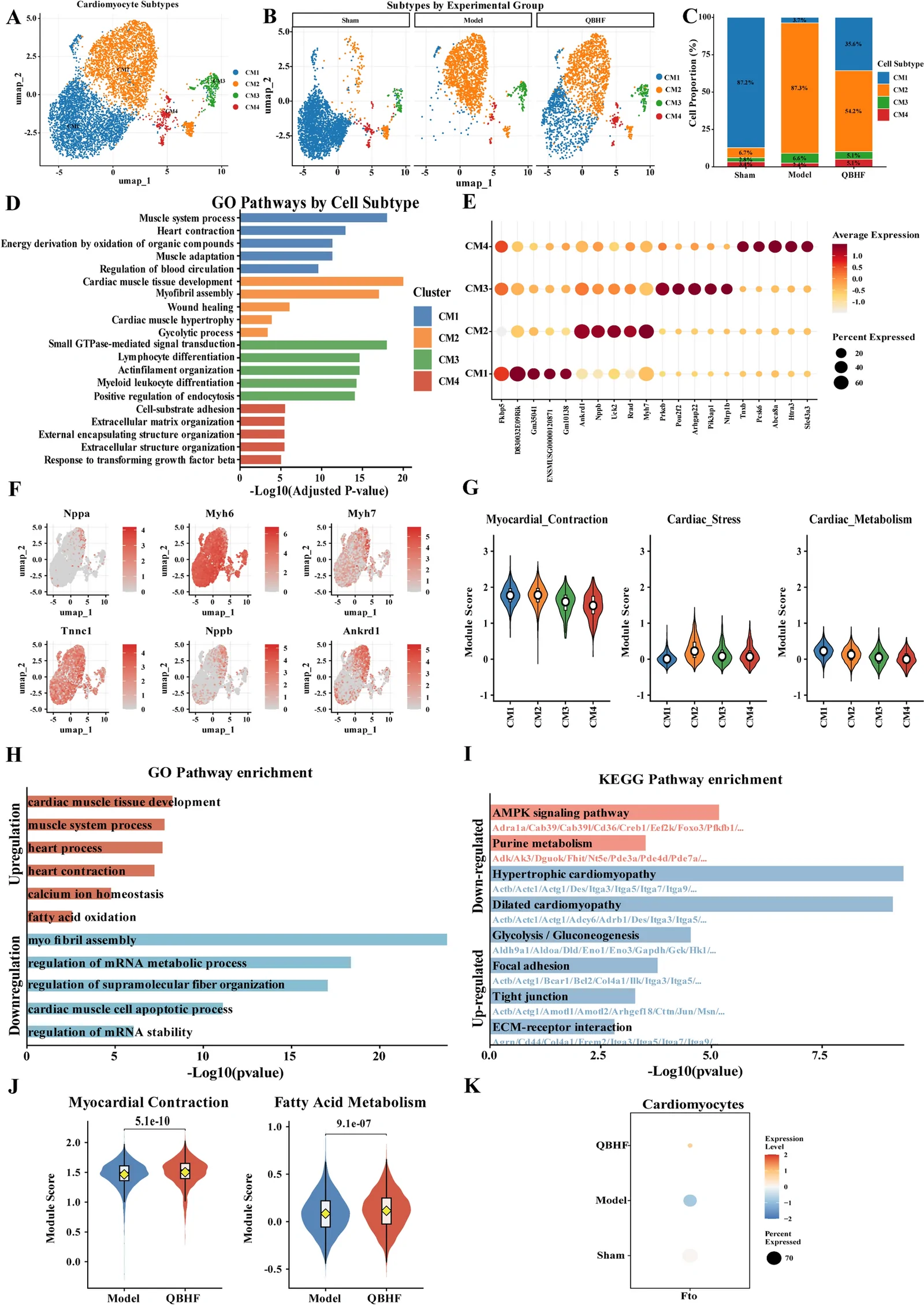
SnRNA-seq reveals transcriptional reprogramming of cardiomyocytes after QBHF intervention. **A** UMAP visualization of cardiomyocytes, colored by the four identified transcriptional subpopulations. **B** UMAP projection of cardiomyocytes, colored by experimental group (sham, model, QBHF). **C** Relative abundance of the cardiomyocyte subpopulations across groups. **D** GO enrichment of subpopulation-specific DEGs. **E** Dot plot of subpopulation-specific marker genes across the four identified cardiomyocyte subpopulations. **F** FeaturePlot visualization of representative marker genes on the UMAP coordinates. **G** Bar plot of average functional scores for myocardial contraction, cardiac stress, and metabolism across cardiomyocyte subpopulations. **H** GO terms enriched in cardiomyocyte DEGs comparing QBHF with model group. **I** KEGG enrichment of DEGs. **J** Functional comparison of myocardial contraction and fatty acid metabolism scores between QBHF and model groups. **K** Bubble plot of FTO expression in cardiomyocytes across the sham, model, and QBHF groups

Cardiomyocytes were resolved into four transcriptionally distinct subpopulations (Fig. 7A, B, D, E). Cardiomyocyte subpopulation 1 (CM1) specifically expressed *Fkbp5* and was enriched in muscle system processes, heart contraction, energy metabolism by oxidation of organic compounds, muscle adaptation, and cardiac blood circulation. This profile indicates an essential role in sustaining cardiac functional and metabolic homeostasis (Fig. 7D–F). Cardiomyocyte subpopulation 2 (CM2), marked by high expression of classic markers such as *Ankrd1*, *Nppa*, and *Myh7* (Fig. 7E, F), was associated with GO terms linked to cardiac muscle tissue development, myofibril assembly, wound healing, cardiac hypertrophy, and glycolytic processes. This profile reflects an adaptive stress-response phenotype that may drive pathological remodeling (Fig. 7D). Cardiomyocyte subpopulation 3 (CM3) expressed *Prkcb, Pik3ap1*, and *Nlrp1b*, and was enriched in immune-related pathways (Fig. 7D–F), suggesting a non-canonical immunoregulatory role during cardiac inflammation and injury (Fig. 7D). Cardiomyocyte subpopulation 4 (CM4) displayed high levels of matrix genes, like *Tnxb* and *Htra3*, and was enriched in extracellular matrix pathways. This indicates its involvement in maintaining myocardial structure and facilitating matrix remodeling (Fig. 7D–F). Gene expression scoring analysis of the cardiomyocyte subpopulations across key cardiac function-related gene sets confirmed their functional specializations (Fig. 7G). CM1 scored higher for myocardial contraction and cardiac metabolism, whereas CM2 scored higher for cardiac stress response (Fig. 7G). Analysis of cardiomyocyte subpopulations distribution across different experimental groups revealed disease-linked dynamics in CM1 and CM2. CM1 abundance was significantly reduced in the model group compared to sham controls, and the loss was restored by QBHF. Conversely, the progressive expansion of CM2 after MI was effectively suppressed by QBHF intervention (Fig. 7C).

To define the regulatory effects of QBHF on cardiomyocytes, the transcriptomic profiles were compared between the QBHF and model groups. GO enrichment analysis revealed that up-regulated genes following QBHF intervention were enriched in cardiac muscle tissue development, muscle system processes, heart contraction, calcium-ion homeostasis, and fatty acid oxidation. In contrast, down-regulated genes were predominantly associated with myofibril assembly, mRNA metabolism regulation, supramolecular fiber organization, cardiomyocyte apoptosis, and mRNA stability (Fig. 7H). KEGG analysis further indicated that up-regulated DEGs mapped to the AMPK signaling and purine metabolism, while down-regulated DEGs were largely linked to hypertrophic cardiomyopathy, glycolysis/gluconeogenesis, and ECM-receptor interaction pathways (Fig. 7I). Moreover, QBHF-treated hearts exhibited significantly higher scores for both cardiac muscle contraction and fatty acid metabolism than the model group, reflecting improved myocardial function and metabolic activity (Fig. 7J). Finally, FTO expression in cardiomyocytes was reduced after MI and partially restored by QBHF (Fig. 7K), suggesting that FTO-mediated m^6^A demethylation contributes to the observed improvements in cardiac function and metabolism.

In summary, snRNA-seq in this study reveals a dual cellular mechanism by which QBHF promotes post-MI recovery. First, QBHF mitigates fibrosis by inhibiting the activation of the pro-fibrotic fibroblast and reducing collagen deposition. Second, QBHF preserves the CM1 cardiomyocyte subpopulation that sustains contractile and oxidative-metabolic function, thereby supporting the observed gains in systolic performance and energy metabolism. Restoration of FTO expression in fibroblasts and cardiomyocytes constitutes an early shared upstream event that positions FTO as a central mediator of QBHF’s cardioprotective effects.

### FTO is an essential mediator of QBHF-induced cardioprotection against pathological remodeling after MI

**Fig. 8:**
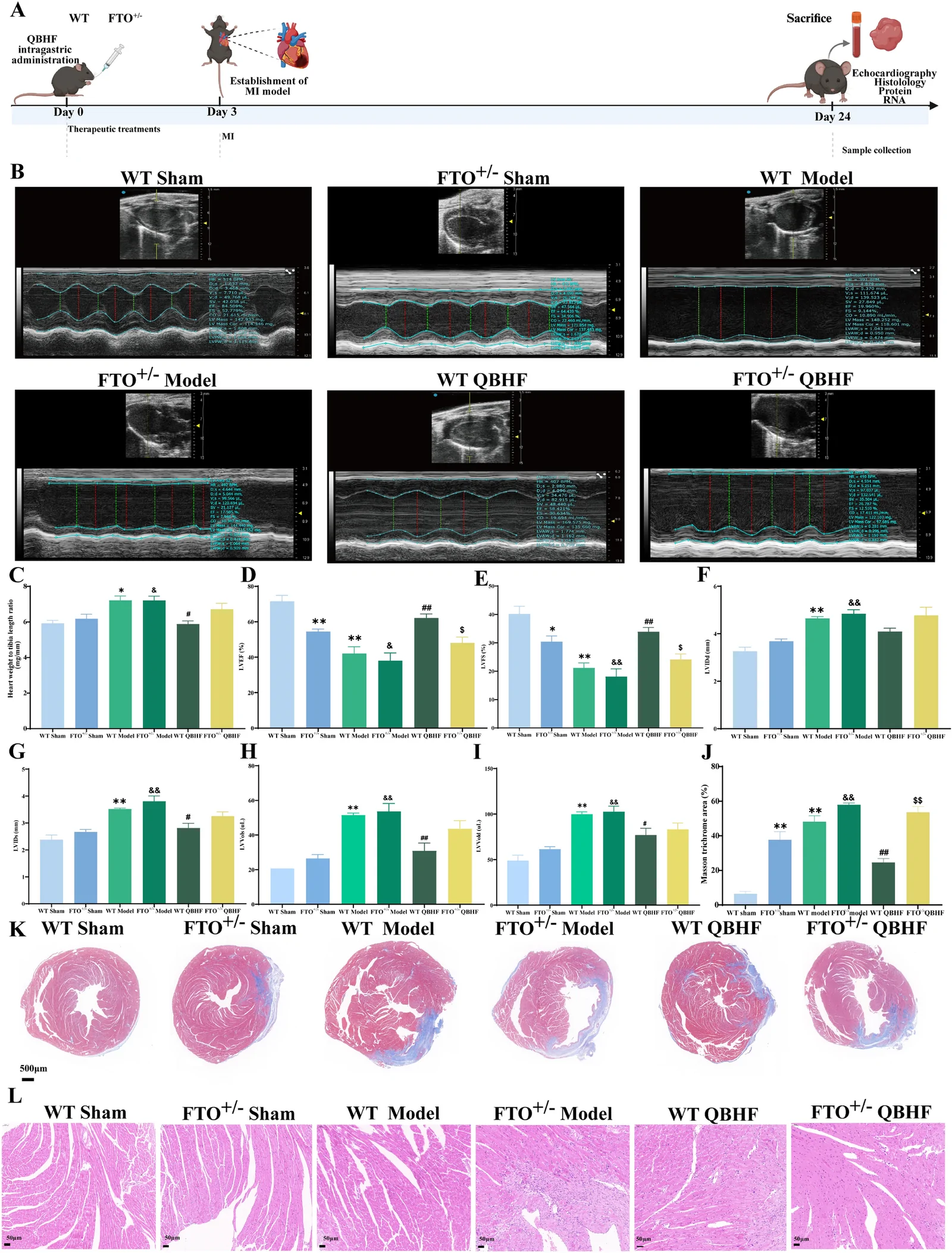
Heterozygous FTO knockout attenuates QBHF-mediated cardioprotective effects after MI. **A** Experimental workflow. **B** Representative M‑mode echocardiograms. **C** HW/TL ratio (n = 6). **D**–**I** Echocardiographic parameters of left ventricular function and geometry: LVEF, LVFS, LVIDd, LVIDs, LVVols, and LVVold (n = 6). **J** Quantitative analysis of myocardial fibrosis area. **K** Representative Masson’s trichrome‑stained sections (collagen shown in blue). **L** Representative H&E-stained myocardial sections (n = 6). Data are presented as the mean ± SEM. *P < 0.05, **P < 0.01 vs. WT sham group; ^#^*P* < 0.05, ^##^*P* < 0.01 vs. WT model group; ^&^*P* < 0.05, ^&&^*P* < 0.01 vs. FTO⁺_/−_ sham group; ^$^*P* < 0.05, ^$$^*P* < 0.01 vs. FTO⁺_/−_ QBHF group

Transcriptomic analyses indicated that QBHF restores the RNA demethylase FTO, and the requirement of FTO for cardioprotection was therefore examined. Homozygous deletion of FTO is embryonic lethal; therefore, heterozygous FTO knockout (FTO⁺_/__−_) mice were used in this study. Cardiac function and tissue remodeling were assessed by echocardiography, H&E staining, and Masson’s trichrome staining in FTO⁺_/−_ mice subjected to MI (Fig. 8A).

Echocardiographic assessment showed that MI induced significant cardiac hypertrophy and functional impairment in WT mice, as indicated by an elevated HW/TL ratio, LVIDd, LVIDs, LVVold, and LVVols, in conjunction with reduced LVEF and LVFS compared to sham (*P* < 0.05). QBHF significantly reversed these changes (*P* < 0.05). FTO⁺_/−_ sham mice showed a higher HW/TL ratio and lower baseline LVEF/LVFS than WT sham mice (*P* < 0.05). MI further impaired cardiac function and elevated HW/TL ratio in FTO⁺_/−_ mice, but QBHF did not significantly improve either parameter in the heterozygous background (Fig. 8B–I).

Masson’s trichrome staining revealed the increased myocardial fibrosis in WT mice post-MI, which was attenuated by QBHF (*P* < 0.05). Notably, FTO⁺_/−_ sham mice exhibited greater baseline fibrosis than WT sham mice, and this fibrosis was further exacerbated by MI (*P* < 0.01). In contrast to the findings in WT mice, QBHF failed to ameliorate fibrotic area in FTO⁺_/−_ MI hearts (Fig. 8J, K).

Histopathological analysis using H&E staining revealed significant myocardial damage in WT mice. This included myocyte swelling, myofibril disarray, interstitial edema, inflammatory infiltration, and thinned left ventricular wall. QBHF markedly attenuated these lesions. In contrast, FTO⁺_/−_ sham-operated hearts exhibited an elevated baseline inflammatory status. Furthermore, following MI, FTO⁺_/−_ mice developed more severe structural damage compared with WT mice. QBHF did not significantly ameliorate these alterations (Fig. 8L).

Histopathological analysis using immunohistochemistry (IHC) staining revealed that Collagen Type I protein expression was significantly upregulated in the MI model group compared with the sham group. QBHF treatment markedly reduced Collagen Type I expression levels. However, in FTO⁺_/−_ mice, although Collagen Type I expression was elevated in the FTO⁺_/−_ model group relative to the FTO⁺_/−_ sham group, QBHF treatment failed to significantly decrease Collagen Type I levels, indicating that the anti-fibrotic effect of QBHF is FTO-dependent (Fig. S2).

**Fig. 9:**
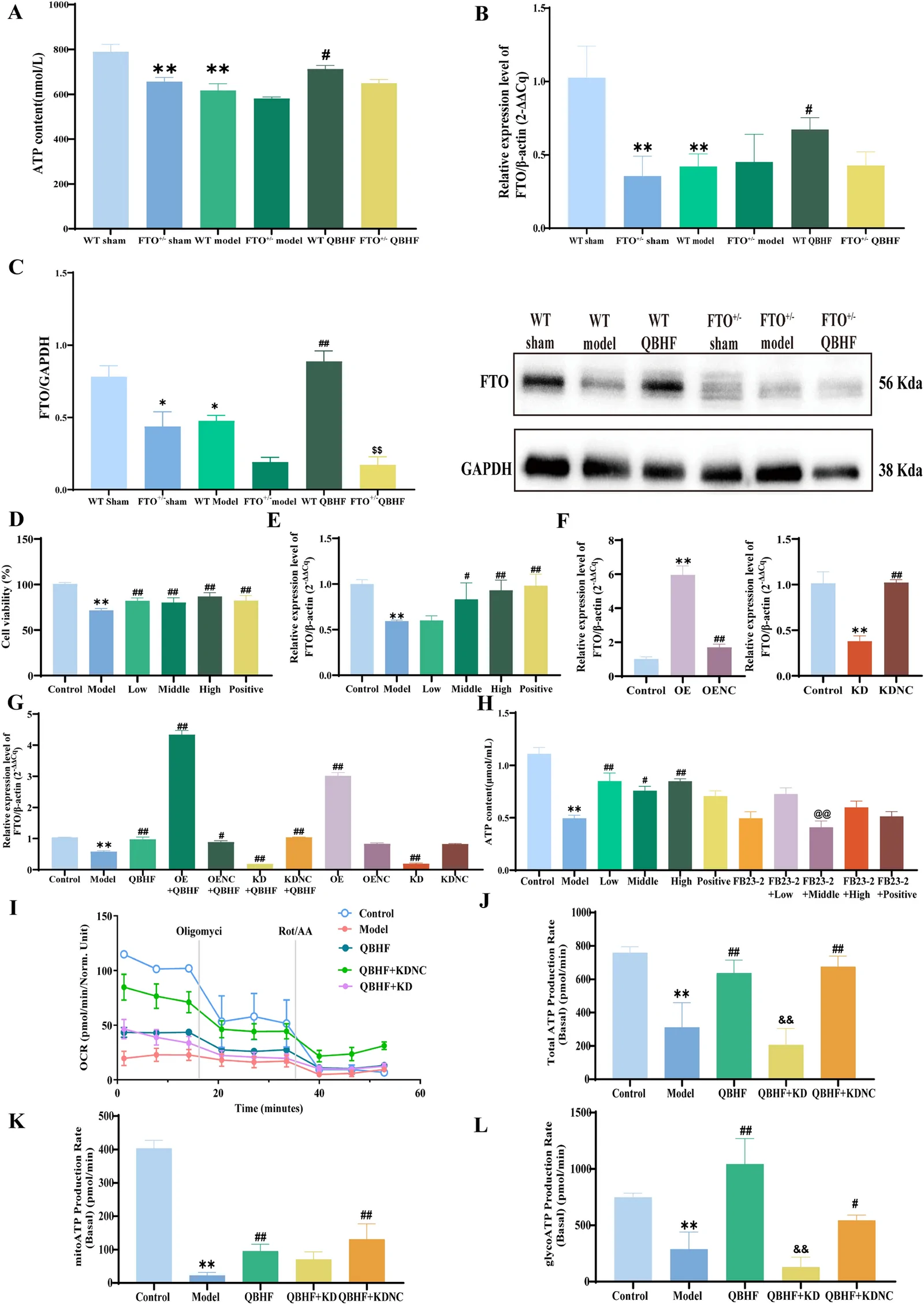
QBHF enhances cardiomyocyte energy metabolism via FTO upregulation *in vitro*. **A** Myocardial ATP levels across groups (n = 6). **B** FTO mRNA expression in cardiac tissue of FTO^+/−^ mice determined by RT-qPCR (n = 6). **C** Representative western blot of FTO protein expression in heart; GAPDH loading control (n = 4). **D** Cell viability assay of OGD-injured cardiomyocytes treated with graded concentrations of QBHF-serum. **E** FTO mRNA expression in cardiomyocytes treated with different doses of QBHF-serum measured by RT-qPCR (n = 6). **F** Efficiency of FTO overexpression via lentiviral transduction assessed by RT-qPCR (n = 6). **G** FTO expression across experimental groups determined by RT-qPCR. **H** ATP levels in cardiomyocytes across groups (n = 6). **I**–**L** ATP production rates determined by Seahorse extracellular flux analysis in AC16 cardiomyocytes under the indicated conditions (n ≥ 4). **I** Representative OCR traces. Gray lines indicate the addition of mitochondrial functional modulators (oligomycin, rotenone/antimycin A [Rot/AA]). Vertical axis: oxygen consumption rate (OCR); horizontal axis: time. **J** Total ATP production rate. **K** Mitochondrial ATP production rate (aerobic respiration). **L** Glycolytic ATP production rate (anaerobic respiration). Data are presented as the mean ± SEM. In **A**–**C**: *P < 0.05, **P < 0.01 vs. sham group; ^#^*P* < 0.05, ^##^*P* < 0.01 vs. model group; ^$$^*P* < 0.01 vs. FTO^+/-^ QBHF group. In **D**–**K**: **P < 0.01 vs. control group; ^#^*P* < 0.05, ^##^*P* < 0.01 vs. model (OGD) group; ^@@^*P* < 0.01 vs. middle (middle dose QBHF-serum) group; ^&&^*P* < 0.01 vs. QBHF group

RT-qPCR and Western blot showed reduced FTO mRNA and protein levels in WT MI mice, while both were robustly restored by QBHF. This restoration was absent in QBHF-treated FTO⁺_/−_ mice (Fig. 9B). Consistently, FTO protein remained low in FTO⁺_/−_ mice regardless of intervention (Fig. 9C).

In summary, genetic ablation of FTO diminishes the therapeutic benefits of QBHF on cardiac function, structure, fibrosis, and energy metabolism, supporting the conclusion that FTO is an indispensable mediator of QBHF-induced cardioprotection.

### QBHF improves cardiomyocyte energy metabolism via FTO up-regulation *in vitro*

Hearts of MI mice showed a significant decrease in ATP, which was restored by QBHF intervention. In contrast, FTO⁺_/−_ hearts displayed lower baseline ATP, and did not respond to QBHF (Fig. 9A). These findings suggest that FTO is crucial for QBHF-mediated preservation of myocardial energy metabolism.

Since snRNA-seq indicated cardiomyocytes as the primary target, AC16 cells were used to investigate the mechanism. CCK-8 assays were performed to evaluate the effects of different treatments on cell viability. Compared to model (OGD), 10% serum containing low-, middle-, or high-dose QBHF, as well as positive drug-serum, significantly increased viability, with high-dose QBHF-serum yielding the maximal effect (Fig. S1A-C). Therefore, high-dose (10%) QBHF-serum was used for all subsequent experiments (Fig. 9D).

Ischemia was simulated *in vitro* by subjecting AC16 cardiomyocytes to OGD, which mimicked the down-regulation of FTO transcription observed in MI hearts. QBHF-serum intervention and positive drug-serum significantly reversed this suppression and restored FTO transcript levels, supporting a role for QBHF in maintaining FTO expression during hypoxia stress (Fig. 9E).

FTO-overexpressing (FTO-OE) and FTO-knockdown (FTO-KD) AC16 cell lines were generated via lentiviral transduction. RT‑qPCR confirmed efficient FTO modulation at 72 h post-transduction (Fig. 9F, G). QBHF-serum intervention further elevated FTO transcript in FTO-OE cells but failed to upregulate FTO in FTO-KD cells (Fig. 9F), indicating that an intact endogenous FTO regulatory circuit is required for QBHF-mediated induction.

Functional assays demonstrated that QBHF-serum restored intracellular ATP levels in OGD-stressed cardiomyocytes; this effect was abrogated by intervention with the selective FTO inhibitor FB23-2 (10 μmol/L; Fig. 9H and Fig. S1D). Analysis of ATP production rates using the Seahorse extracellular flux analyzer confirmed that QBHF-serum significantly increased ATP generation under OGD. However, this enhancement was abolished in FTO-KD cells, and ATP levels remained equivalent to those of the OGD model group (Fig. 9I–L). These metabolic defects recapitulate the energy deficit observed in the LAD-induced MI model.

Together, these results confirm that QBHF-serum exerts its cardioprotective effects through an FTO-dependent mechanism. QBHF-serum requires functional FTO to enhance cardiomyocyte viability and restore energy metabolism under ischemic conditions, establishing FTO as a critical mediator of QBHF’s therapeutic action.

## Discussion

Myocardial ischemia precipitates a cascade of cardiomyocyte death, energy dysmetabolism, and maladaptive remodeling that culminates in heart failure. Although timely reperfusion combined with standard pharmacotherapy remains the cornerstone of MI therapy, these interventions are largely insufficient to counteract ischemia-induced metabolic dysfunction, inflammatory injury, or fibrosis, underscoring the need for adjunctive strategies with multi-target activity [113]. This study provides robust evidence that the traditional formula QBHF confers broad and significant cardioprotection in a mouse model of permanent LAD-induced MI. QBHF improved cardiac function, limited infarct size, reduced fibrosis, and lowered CK-MB release, while preserving mitochondrial ultrastructure and restoring ATP production. These data indicate that QBHF simultaneously restrains fibrosis and restores energy metabolism after infarction. To elucidate the underlying mechanisms, an integrated multi-omics pipeline combining bulk RNA-seq, non-targeted metabolomics, and snRNA-seq was developed. Bulk transcriptomics indicated that QBHF reinstates metabolic and contractile gene programs while suppressing pro-fibrotic and pro-inflammatory signatures. Metabolomics revealed that QBHF modulates key metabolic pathways, including pyruvate metabolism, glycine/serine/threonine metabolism, and AMPK signaling. Together, these pathways rebalance energy supply. snRNA-seq resolved cell-type-specific responses. QBHF preserved a CM1 cardiomyocyte subpopulation enriched for oxidative metabolism and contractile genes. It also attenuated a fibroblast-1 subpopulation characterized by TGF-β-driven ECM production. Cross-platform integration revealed that the RNA demethylase FTO was consistently up-regulated and emerged as a key hub gene in both fibroblasts and cardiomyocytes after QBHF intervention. This finding suggests its pivotal role in the therapeutic response. Functional validation confirmed FTO as a required mediator. Specifically, heterozygous knockout of FTO attenuated QBHF-induced improvements in left ventricular systolic function, fibrosis, and energy recovery.

**Fig. 10:**
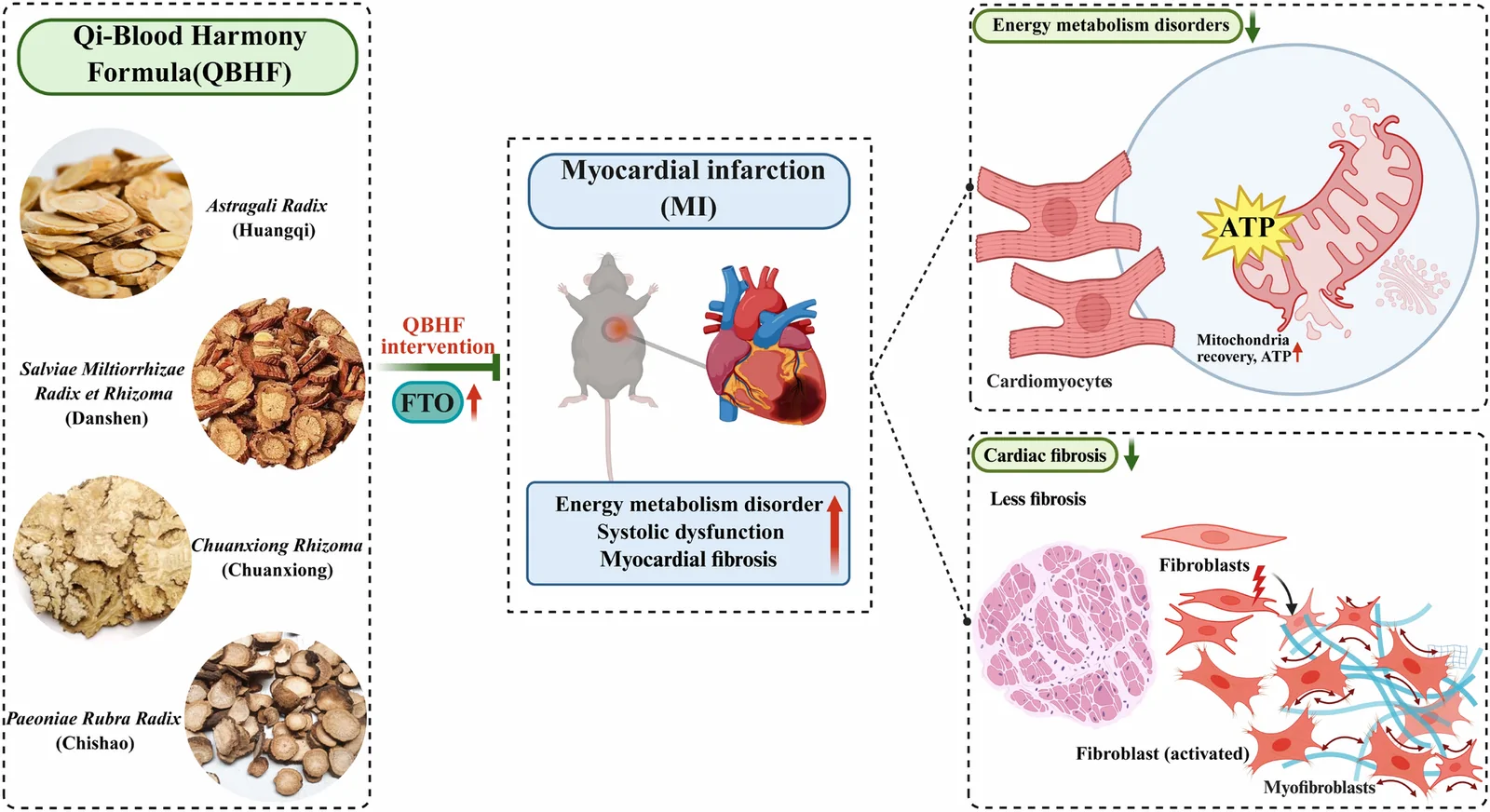
Summary: The traditional Chinese medicine QBHF provides comprehensive cardioprotection after MI via FTO-mediated amelioration of both metabolic dysfunction and fibrosis, thereby providing a multi-dimensional therapeutic approach to prevent MI progression

In OGD-stressed AC16 cardiomyocytes, QBHF-serum enhanced cell viability and restored ATP levels; these benefits were significantly attenuated upon FTO-specific knockout, confirming direct FTO dependence. By synchronously limiting maladaptive fibrosis and restoring energetic competence, QBHF achieves a cardioprotective phenotype that extends beyond the reach of current mono-target therapies (Fig. 10).

Under physiological conditions, the heart generates approximately 90% of its ATP demand through mitochondrial oxidative phosphorylation, predominantly via fatty acid oxidation, with glycolysis supplying the remainder [114, 115]. Following MI, ischemia forces a rapid metabolic switch from this efficient fatty acid β-oxidation to glycolysis [116]. This shift is initially adaptive, but becomes maladaptive over time, driving mitochondrial damage, ATP depletion, and contractile failure [117, 118]. The molecular drivers behind this transition remain incompletely defined, yet recent studies highlight the role of epitranscriptomic regulation, particularly the m^6^A demethylase FTO [119]. Initially linked to systemic energy balance and obesity [120], FTO is now known to be essential for contractility and limiting pathological remodeling [121]. FTO sustains cardiac energy metabolism by maintaining mitochondrial function and promoting mitochondrial biogenesis, as demonstrated in myocardial ischemia/reperfusion injury models [28]. In addition, it regulates glycolysis and glucose utilization via m^6^A-sensitive targets such as PGAM2 and the AKT-GLUT4 axis [30, 122]. Under stress conditions such as hypoxia, reduced FTO expression exacerbates apoptosis and inflammation [123–125]. Furthermore, FTO exerts direct anti-fibrotic effects in the infarcted heart. Overexpression of FTO attenuates cardiac fibrosis in murine MI models [29, 126]. Given FTO’s pivotal role in cardiac energy metabolism and fibrosis, its involvement in the cardioprotective effects of QBHF was explored. Integrative multi-omics and pharmacodynamic analyses revealed that QBHF up-regulates FTO, which in turn improves cardiac function, reduces infarct size and fibrosis, and restores energy metabolism. These findings establish FTO as a central hub that mediates the cardioprotective effects of QBHF. These benefits were critically FTO-dependent: QBHF up-regulated FTO and exerted cardioprotection, yet all structural, functional, and metabolic benefits were abolished in FTO heterozygous knockout mice. Thus, QBHF limits fibrosis and enhances energy metabolism through FTO-dependent coordination of interconnected pathways, underscoring its comprehensive therapeutic potential. Consistent with this framework, our bulk RNA-seq analysis identified several downstream candidate effectors whose expression was coordinately altered following QBHF intervention. Among these, *Ppargc1a*, *Acsl1*, *Col1a1*, *Acta2*, and *Smad3* have been independently reported as FTO-regulated targets in various disease contexts, including myocardial ischemia/reperfusion, cardiac fibrosis, and ischemic stroke [28, 127–130]. The convergence of our transcriptomic data with these published findings suggests that QBHF, through upregulation of FTO, may coordinately modulate the expression of these functionally related transcripts to achieve synergistic cardioprotection. However, direct m^6^A modification evidence on these transcripts in the context of QBHF treatment remains to be established, and the full spectrum of FTO-dependent m^6^A modifications awaits systematic characterization in future studies.

Over the past decades, TCM contributed significantly to both primary prevention and post-infarction treatment of MI [131, 132]. Increasing evidence supports that herbal formulations alleviate ischemic injury by modulating inflammation, fibrosis, and metabolic dysfunction, with fewer adverse effects than Western medicine [133–135]. Guided by the principle of restoring physiological balance, TCM exerts cardioprotection through coordinated multi-pathway regulation, offering a holistic therapeutic paradigm [136]. QBHF, a representative formulation, consists of four traditional herbs: *Astragali Radix* (Huang Qi), *Salviae Miltiorrhizae Radix* (Dan Shen), *Chuanxiong Rhizoma* (Chuan Xiong), and *Paeoniae Radix Rubra* (Chi Shao). Each herb synergistically enhances the overall therapeutic effect. QBHF exhibits substantial chemical complexity, with 67 compounds identified across multiple structural classes, predominantly terpenoids, phenolic acids, and flavonoids [137]. Among these, paeoniflorin and salvianolic acid B were the most abundant constituents. Serum pharmacology analyses reveal that multiple prototype components reach systemic circulation and are considered the principal bioactive mediators of QBHF efficacy. These include calycosin and biochanin A (from *Astragali Radix*), paeonol (from *Paeoniae Radix Rubra*), and ferulic acid (from *Chuanxiong Rhizoma*) [138, 139]. Notably, astragaloside IV, one of the major constituents identified in QBHF, has been independently shown to modulate FTO-mediated m^6^A methylation [140], suggesting that specific QBHF components may possess FTO-modulating activity and providing a plausible mechanistic link that warrants further investigation. The individual herbs contribute distinct yet complementary mechanisms: *Astragali Radix* improves mitochondrial energetics [50]; *Salviae Miltiorrhizae Radix* promotes blood circulation and alleviates ischemic injury and fibrosis [141–143]; *Chuanxiong Rhizoma* attenuates fibroblast activation and fibrotic remodeling [144]; and *Paeoniae Radix Rubra* suppresses pro-inflammatory pathways during post-MI repair [145]. Altogether, the QBHF herbs, their native components, and their bioavailable metabolites jointly attenuate myocardial fibrosis and enhance energy metabolism. This dual therapeutic benefit is achieved through the coordinated modulation of fibrotic and metabolic pathways, providing comprehensive cardiac protection.

This study revealed that QBHF confers comprehensive, multi-target cardioprotection by simultaneously targeting fibrosis and energy metabolism. Nevertheless, four limitations merit consideration. First, one limitation is that direct *in vitro* fibroblast-autonomous validation was not performed. Our experimental design was guided by the biological nature of the two processes examined. Cardiomyocyte energy metabolism is a cell-autonomous and dynamically measurable process, making *in vitro* cardiomyocyte models well suited for functional validation. By contrast, post-MI fibrosis is a tissue-level remodeling process involving multicellular interactions within the injured myocardium, for which *in vivo* histological assessment and snRNA-seq-based fibroblast profiling provide more physiologically relevant evidence. Nevertheless, future studies using primary cardiac fibroblasts and TGF-β-induced activation models are required to determine whether QBHF directly regulates fibroblast activation through cell-autonomous FTO-dependent mechanisms. Second, although UHPLC-MS identified multiple bioactive components of QBHF in systemic circulation, the specific compound responsible for FTO upregulation remains to be identified. To systematically identify the direct molecular target(s) of QBHF, future studies will integrate serum pharmacochemistry with advanced target fishing strategies, including affinity-based proteomics approaches (e.g., SILAC, CETSA, DARTS) [146–148], TCM-microsphere-based photo-affinity platforms [149], and complementary computational methods such as molecular docking and machine learning-based target prediction [150–152]. Third, the full repertoire of FTO-dependent m^6^A modifications underlying QBHF’s effects awaits comprehensive mapping. Future studies will employ multi-dimensional epigenomic profiling to characterize FTO-mediated m^6^A regulation, including methylated RNA immunoprecipitation sequencing (MeRIP-seq), cross-linking and immunoprecipitation high throughput sequencing (CLIP-seq), assay for Transposase-Accessible Chromatin with high-throughput sequencing (ATAC-seq), and chromatin Immunoprecipitation followed by sequencing (ChIP-seq) [153, 154]. Subsequently, candidate targets will be functionally validated through knockdown/overexpression and site-specific m^6^A reporter assays in the context of QBHF treatment. Finally, findings obtained in an LAD-induced mouse model require verification in larger species and ultimately in humans.

## Conclusion

Myocardial energy-metabolism dysregulation and fibrosis are central drivers of adverse outcome after MI and strongly influence long-term prognosis, yet no universally established therapy specifically corrects these defects. This study shows QBHF confers potent, multi-cellular cardioprotection by simultaneously restoring energetic competence and limiting fibrotic remodeling. Multi-omics integration and functional validation identify FTO as the indispensable mediator through which QBHF orchestrates these benefits. The findings reveal tractable pharmacological targets for MI management, provide a mechanistic rationale for clinical deployment of QBHF, and exemplify how integrated omics can decode the therapeutic logic of traditional medicine.

## Supplementary Information

Supplementary Material 1.

## Data Availability

All data generated in this study will be made available on request.

## Abbreviations

MI: Myocardial infarction
DEGs: Differentially expressed genes
ELISA: Enzyme-linked immunosorbent assay
ESI: Electrospray ionization
FTO: Fat mass and obesity-associated protein
GO: Gene ontology
LVEF: Left ventricular ejection fraction
LVFS: Left ventricular fractional shortening
LVIDd: Left ventricular end-diastolic internal diameter
LVIDs: Left ventricular end-systolic internal diameter
LVVold: Left ventricular end-diastolic volume
LVVols: Left ventricular end-systolic volume
m^6^A: *N*6-methyladenosine
PCA: Principal component analysis
PCI: Percutaneous coronary intervention
QBHF: Qi-Blood Harmony Formula
snRNA-seq: Single-nucleus RNA sequencing
TCM: Traditional Chinese medicine
t-SNE: T-stochastic neighbor embedding
TTC: 2, 3, 5-Triphenyl tetrazolium chloride
UMAP: Uniform manifold approximation and projection
