# Supplementary material for: Qi-Blood Harmony Formula mediates post-MI cardioprotection via FTO-driven dual modulation of energy metabolism and fibrosis: a multi-omics validation
Source: Chin Med. 2026 Nov 2;21:235. doi: 10.1186/s13020-026-01506-w (PMC13726175; doi:10.1186/s13020-026-01506-w)
Supplement: Supplementary file 1 [file 13020_2026_1506_MOESM1_ESM.docx]

**Supporting Material**

**1. Supplemental methods**

## **1. Identification of QBHF *in vitro* and *in vivo* by ultra-high-performance liquid chromatography-mass spectrometry (UHPLC-MS/MS)**

The chemical profile of the QBHF *in vitro* and *in vivo* was determined using UHPLC-MS/MS. To preserve chemical integrity, sample preparation was conducted on ice. In brief, 100 μL of extract was mixed with 800 μL of pre-chilled methanol in a 2.0 mL Eppendorf tube. The mixture was vortexed (2 minutes) and subsequently subjected to ice-bath ultrasonication for extraction [1]. The extract of the supernatant was subsequently concentrated at low temperature and stored at -20 °C.

Before analysis, samples were reconstituted in 100 μL of 20% methanol/water (v/v), vortexed until fully dissolved, and centrifuged again to remove particulates. The blank control used a 20% methanol/water solution. UHPLC-MS/MS analysis was conducted on an AB SCIEX UHPLC system with an X500R high-resolution mass spectrometer, controlled by OS data acquisition software. LC-MS data acquisition was performed in both positive and negative ionization modes to ensure comprehensive metabolite coverage [2]. Compound identification was achieved by matching experimental spectra against the MSMS_Public_ExpBioInsilico_NEG_VS17.msp database employing MS-DIAL software [2]. Detailed chromatographic and mass spectrometry parameters are listed in Supplementary table 1. In negative and positive ionization modes, 381 and 559 QBHF-related ions were detected, respectively (Fig. S1E-F, Supplementary table 2-3). These features correspond to flavonoids, phenolic acids, coumarins, terpenoids (e.g., diterpenoids and triterpenoids), and lignans. Serum pharmacochemical analysis further confirmed that multiple prototype constituents and their metabolites were present in the bloodstream following QBHF administration, as detailed in Supplementary tables 5-6.

## **2. Immunohistochemistry Analysis of Collagen Type I**

Cardiac specimens were fixed with 4% paraformaldehyde, embedded in paraffin, and cut into 3-μm-thick sections. After deparaffinization and gradient rehydration, heat-mediated antigen retrieval was carried out using citrate buffer (pH 6.0) at 95°C for 20 min. To block endogenous peroxidase activity, sections were treated with 0.3% hydrogen peroxide for 25 min, followed by incubation with 5% goat serum for 20 min to reduce nonspecific binding. The slides were then probed with anti-Collagen Type I primary antibody (66761-1, Proteintech Group, Inc., Wuhan, China) overnight at 4°C, and subsequently incubated with HRP-labeled secondary antibody for 2 h at 37°C. Color development was achieved using DAB substrate for 5 min, after which the sections were counterstained with hematoxylin, dehydrated through an ascending ethanol series, and mounted with coverslips. Imaging was performed under a Nikon ECLIPSE E100 microscope.

**2. Supplemental tables**

## **2.1 Supplementary Table 1. UHPLC-MS/MS analysis parameters for QBHF extracts UHPLC conditions**

***Chromatographic Conditions***

| **Ion Mode** | **Column** | **Mobile Phases (A/B)** | **Flow Rate** | **Gradient Program** | **Column Temp** |
| --- | --- | --- | --- | --- | --- |
| Positive | Waters ACQUITY UPLC® BEH C8 (1.7 μm, 2.1×100 mm) | A: 0.1% Formic Acid in Water B: 0.1% Formic Acid in Acetonitrile | 0.3 mL/min | 0-2 min: 5% B 2-37 min: 5%→99% B 37-42 min: 100% B 42.1-45 min: 5% B | 50 °C |
| Negative | Waters ACQUITY UPLC® HSS T3 (1.8μm, 2.1×100 mm) | A: 6.5 mM Ammonium Bicarbonate in Water B: 6.5 mM Ammonium Bicarbonate in 90% Methanol | 0.3 mL/min | 0-2 min: 5% B 2-42 min: 5%→99% B 42-47 min: 99% B 47.1-50 min: 5% B | 50 °C |

***Mass Spectrometry Conditions***

| **Ion Mode** | **Instrument** | **Source Temp (°C)** | **Curtain Gas (psi)** | **Delustering Potential (V)** | **MS CE (eV)** | **MS/MS CE (eV)** | **MS1 Range (Da)** | **MS2 Range (Da)** |
| --- | --- | --- | --- | --- | --- | --- | --- | --- |
| Positive | AB SCIEX X500R | 550 | 35 | +80 | +10 | 40 ± 15 | 70-1050 | 50-1050 |
| Negative | AB SCIEX X500R | 350 | 35 | -80 | -10 | -35 ± 15 | 70-1050 | 50-1050 |

## **2.2 Supplementary Table 2. Chemical annotation of QBHF-related MS features in traditional Chinese medicines (negative-ion mode).**

| **Name** | **RT (min)** | **Precursor m/z** | **Area** | **Reference m/z** | **Formula** | **Ontology** |
| --- | --- | --- | --- | --- | --- | --- |
| Palmitic Acid | 32.48 | 255.23362 | 14715 | 255.233 | C16H32O2 |  |
| Ursolic Acid | 29.90 | 455.35422 | 8839 | 455.35306 | C30H48O3 | Triterpenoids |
| 16-Hydroxyhexadecanoic acid | 29.02 | 271.22871 | 10952 | 271.22733 | C16H32O3 | Long-chain fatty acids |
| 2-hydroxy-6-[(8Z,11Z)-pentadeca-8,11,14-trienyl]benzoic acid | 28.23 | 341.21253 | 76181 | 341.21439 | C22H30O3 |  |
| (E)-5-(1,2,4a,5-tetramethyl-7-oxo-3,4,8,8a-tetrahydro-2H-naphthalen-1-yl)-3-methylpent-2-enoic acid | 28.07 | 317.21271 | 100726 | 317.21222 | C20H30O3 | Colensane and clerodane diterpenoids |
| 12-Hydroxyoctadecanoic acid | 26.98 | 299.25933 | 477923 | 299.25919 | C18H36O3 | Long-chain fatty acids |
| 2,6-Di-tert-butyl-4-nitrophenol | 26.91 | 250.14561 | 8531 | 250.1449 | C14H21NO3 | Nitrophenols |
| 12-HEPE | 26.75 | 317.2126 | 303197 | 317.21219 | C20H30O3 | Hydroxyeicosapentaenoic acids |
| FA 18:3+1O | 25.80 | 293.21234 | 318442 | 293.2121 | C18H30O3 | Oxidized fatty acids |
| Triptophenolide | 25.73 | 311.16935 | 59504 | 311.16525 | C20H24O3 | Oxosteroids |
| 5,7-dihydroxy-2-(4-hydroxyphenyl)-8-[3,4,5-trihydroxy-6-(hydroxymethyl)oxan-2-yl]-6-(3,4,5-trihydroxyoxan-2-yl)chromen-4-one | 25.68 | 563.20778 | 20156 | 563.20978 | C26H28O14 | Flavonoid 8-C-glycosides |
| FA 18:1+1O | 25.59 | 297.24465 | 11334 | 297.24399 | C18H34O3 | Lineolic acids and derivatives |
| 3,7-Epoxycaryophyllan-6-One | 25.48 | 235.17103 | 8694 | 235.17035 | C15H24O2 | Oxepanes |
| MGMG 18:2; PlaSMA ID-1541 | 25.43 | 561.32893 | 5549 | 561.32898 | C27H48O9 | Lipids |
| (4,7,7-trimethyl-3-bicyclo[2.2.1]heptanyl) (E)-3-(4-hydroxy-3-methoxyphenyl)prop-2-enoate | 25.32 | 329.1763 | 48745 | 329.17581 | C20H26O4 | Coumaric acids and derivatives |
| Hederagenin | 25.00 | 471.3483 | 51427 | 471.34796 | C30H48O4 | Triterpenoids |
| Corosolic acid | 25.00 | 517.3544 | 12161 | 517.35345 | C30H48O4 | Triterpenoids |
| 9-HODE | 24.89 | 295.22872 | 23524 | 295.22791 | C18H32O3 | Lineolic acids and derivatives |
| conocarpan | 24.67 | 265.12392 | 173968 | 265.12338 | C18H18O2 | 2-arylbenzofuran flavonoids |
| 9-hydroxy-5b,8,11a-trimethyl-1-prop-1-en-2-yl-1,2,3,4,5,6,7,7a,9,10,11,11b,12,13,13a,13b-hexadecahydrocyclopenta[a]chrysene-3a,5a,8-tricarboxylic acid | 24.61 | 515.33934 | 9232 | 515.33881 | C30H44O7 | Triterpenoids |
| (1R,2R,4aR,8aS)-1-[2-(furan-3-yl)ethyl]-2,4a,5-trimethyl-2,3,4,7,8,8a-hexahydronaphthalene-1-carboxylic acid | 24.52 | 315.19769 | 197288 | 315.19656 | C20H28O3 | Colensane and clerodane diterpenoids |
| dehydroabietic acid | 24.51 | 299.2023 | 465945 | 299.20163 | C20H28O2 | Diterpenoids |
| beta-Hydroxymyristic acid | 24.47 | 243.19744 | 48704 | 243.19591 | C14H28O3 | Long-chain fatty acids |
| 9-hydroxy-10,12-octadecadienoic acid | 24.45 | 295.22866 | 48263 | 295.22784 | C18H32O3 | Lineolic acids and derivatives |
| FA 18:4+1O | 24.44 | 291.19777 | 14880 | 291.19449 | C18H28O3 | Oxidized fatty acids |
| LPC 16:0 | 24.42 | 540.3322 | 8631 | 540.33069 | C24H50NO7P | Lipids |
| (1S,4aS,5R)-5-[2-(furan-3-yl)ethyl]-1,4a-dimethyl-6-methylidene-3,4,5,7,8,8a-hexahydro-2H-naphthalene-1-carboxylic acid | 24.17 | 315.19675 | 382163 | 315.19656 | C20H28O3 | Colensane and clerodane diterpenoids |
| NCGC00380867-01 | 23.86 | 559.31357 | 7768 | 559.31201 | C27H46O9 | Glycosylmonoacylglycerols |
| LPE 18:2 | 23.76 | 476.27961 | 13947 | 476.27759 | C23H44NO7P | 1-acyl-sn-glycero-3-phosphoethanolamines |
| 1,4a-dimethyl-9-oxo-7-propan-2-yl-3,4,10,10a-tetrahydro-2H-phenanthrene-1-carboxylic acid | 23.21 | 313.18171 | 25933 | 313.18091 | C20H26O3 | Diterpenoids |
| 13-HOTrE | 23.15 | 293.21266 | 104613 | 293.21219 | C18H30O3 | Lineolic acids and derivatives |
| 1,7-bis(4-hydroxyphenyl)heptan-3-one | 22.52 | 297.18708 | 49105 | 297.18283 | C19H22O3 | Linear diarylheptanoids |
| 5-[2-(furan-3-yl)ethyl]-8a-(hydroxymethyl)-5,6-dimethyl-3,4,4a,6,7,8-hexahydronaphthalene-1-carboxylic acid | 22.45 | 331.1921 | 56522 | 331.19119 | C20H28O4 | Colensane and clerodane diterpenoids |
| NCGC00168838-03 | 22.32 | 531.33404 | 8816 | 531.33301 | C30H46O5 | Triterpenoids |
| methyl (2E,4E,8E)-7,13-dihydroxy-4,8,12-trimethyltetradeca-2,4,8-trienoate | 22.25 | 309.20795 | 8926 | 309.20712 | C18H30O4 | Sesquiterpenoids |
| LPC 18:3 | 22.11 | 562.31591 | 40271 | 562.31299 | C26H48NO7P | 1-acyl-sn-glycero-3-phosphocholines |
| 3,12-dihydroxy-4,6a,6b,11,12,14b-hexamethyl-1,2,3,4a,5,6,7,8,9,10,11,12a,14,14a-tetradecahydropicene-4,8a-dicarboxylic acid | 22.05 | 501.32305 | 10293 | 501.32199 | C30H46O6 | Triterpenoids |
| 2-[(3S)-3-hydroxy-4a,8-dimethyl-2,3,4,5,6,7-hexahydro-1H-naphthalen-2-yl]prop-2-enoic acid | 21.91 | 249.15057 | 21138 | 249.14961 | C15H22O3 | Eudesmane, isoeudesmane or cycloeudesmane sesquiterpenoids |
| DGMG 18:3 | 21.86 | 721.36547 | 47054 | 721.36292 | C33H56O14 | Lipids |
| 1-(9Z,12Z-Octadecadienoyl)-2-hydroxy-sn-glycero-3-phosphoethanolamine (NMR) | 21.72 | 475.27091 | 18621 | 475.271 | C23H43NO7P | 1-acyl-sn-glycero-3-phosphoethanolamines |
| 5-hydroxy-2,2-dimethyl-10-(2-methylbut-3-en-2-yl)pyrano[3,2-g]chromen-8-one | 21.29 | 311.13016 | 16368 | 311.12888 | C19H20O4 | Linear pyranocoumarins |
| ent-kauran-17,19-dioic acid | 20.94 | 333.20815 | 22724 | 333.20712 | C20H30O4 | Kaurane diterpenoids |
| Asiatic acid | 20.82 | 533.35032 | 18903 | 533.34991 | C30H48O5 | Triterpenoids |
| ESTRIOL | 20.75 | 269.15589 | 30319 | 269.15469 | C18H22O2 | Estrogens and derivatives |
| FA 18:1+2O | 20.52 | 313.23927 | 277410 | 313.23749 | C18H34O4 | Long-chain fatty acids |
| 9-hydroxy-7-(2-hydroxypropan-2-yl)-1,4a-dimethyl-2,3,4,9,10,10a-hexahydrophenanthrene-1-carboxylic acid | 20.44 | 331.19203 | 147471 | 331.19147 | C20H28O4 | Diterpenoids |
| 1-[3-methoxy-4-[(2S,3R,4S,5S,6R)-3,4,5-trihydroxy-6-[[(2R,3R,4R,5R,6S)-3,4,5-trihydroxy-6-methyloxan-2-yl]oxymethyl]oxan-2-yl]oxyphenyl]ethanone | 20.33 | 473.16176 | 18881 | 473.16644 | C21H30O12 | Phenolic glycosides |
| N-Benzyladenine | 20.27 | 224.09373 | 9871 | 224.09419 | C12H11N5 | 6-alkylaminopurines |
| (9a-hydroxy-3,8a-dimethyl-5-methylidene-2-oxo-4,4a,6,7,8,9-hexahydrobenzo[f][1]benzofuran-8-yl) acetate | 20.03 | 305.14008 | 131308 | 305.13943 | C17H22O5 | Eudesmanolides, secoeudesmanolides, and derivatives |
| (2S)-4-hydroxy-2-(2-hydroxypropan-2-yl)-7-methyl-2,3-dihydrofuro[3,2-g]chromen-5-one | 19.98 | 275.12989 | 27792 | 275.12662 | C15H16O5 | Furanochromones |
| (E)-4-[(1R,2S,3S,4R,8aS)-2,3,4-trihydroxy-2,5,5,8a-tetramethyl-3,4,4a,6,7,8-hexahydro-1H-naphthalen-1-yl]but-3-en-2-one | 19.66 | 309.20772 | 246619 | 309.20712 | C18H30O4 | Sesquiterpenoids |
| Chikusetsusaponin IV | 19.48 | 925.48125 | 26157 | 925.48022 | C47H74O18 | Triterpene saponins |
| 6,8-dihydroxy-2,2,4,4-tetramethyl-5-(2-methylpropanoyl)-9-propan-2-yl-9H-xanthene-1,3-dione | 19.38 | 413.19799 | 8624 | 413.19699 | C24H30O6 | Xanthenes |
| FA 18:5+2O | 19.11 | 305.17657 | 31452 | 305.17459 | C18H26O4 | Lineolic acids and derivatives |
| NCGC00169840-02![2-(4-hydroxy-3-methoxyphenyl)-4-[(4-hydroxy-3-methoxyphenyl)methyl]oxolan-3-yl]methyl (E)-3-(4-hydroxyphenyl)prop-2-enoate | 19.10 | 505.18787 | 27198 | 505.18701 | C29H30O8 | 7,9'-epoxylignans |
| (4aS)-6,7-dihydroxy-1,1,4a-trimethyl-3,4,10,10a-tetrahydro-2H-phenanthren-9-one | 19.04 | 273.15037 | 29023 | 273.1496 | C17H22O3 | Diterpenoids |
| (9-hydroxy-5,8-dimethyl-1-methylidene-2,6-dioxo-4,8a,9,9a-tetrahydro-3aH-azuleno[6,7-b]furan-4-yl) 3-methylbut-2-enoate | 18.75 | 357.13491 | 97051 | 357.13437 | C20H22O6 | Sesquiterpene lactones |
| Biochanin A * | 18.58 | 283.06211 | 16418 | 283.06119 | C16H12O5 | Isoflavone O-glycosides |
| 5-[6-(3-hydroxy-4-methoxyphenyl)-1,3,3a,4,6,6a-hexahydrofuro[3,4-c]furan-3-yl]-2-methoxyphenol | 18.53 | 357.13544 | 65435 | 357.13437 | C20H22O6 | Furanoid lignans |
| [1,3-dihydroxy-1-(7-methoxy-2-oxochromen-6-yl)-3-methylbutan-2-yl] 3-methylbut-2-enoate | 18.44 | 375.19491 | 20483 | 375.194 | C20H24O7 | Coumarins and derivatives |
| 1-[2-hydroxy-4-(3-hydroxy-5-methylphenoxy)-6-methylphenyl]-3-methylbutane-2,3-diol | 18.42 | 331.15573 | 122974 | 331.15509 | C19H24O5 | Diphenylethers |
| FA 18:1+2O; PlaSMA ID-662 | 18.41 | 313.23947 | 8064 | 313.23749 | C18H34O4 | Oxidized fatty acids |
| 3-hydroxy-5,5,8a-trimethyl-3,4,4a,6,7,8-hexahydronaphthalene-2-carboxylic acid | 18.33 | 237.15081 | 10858 | 237.14961 | C14H22O3 | Beta hydroxy acids and derivatives |
| Soyasaponin Bb * | 18.18 | 987.51712 | 290967 | 987.51703 | C48H78O18 | Triterpene saponins |
| Soyasapogenol B base + O-HexA-Pen-dHex | 18.12 | 911.50185 | 19271 | 911.50208 | C47H76O17 | Triterpene saponins |
| 3-[5-(3,4-dihydroxy-4-methyl-5-oxooxolan-2-yl)-2-hydroxy-4-methoxyphenyl]propanoic acid | 17.84 | 325.20286 | 26601 | 325.20618 | C15H18O8 | Phenylpropanoic acids |
| Tridecanedioic acid | 17.84 | 243.16109 | 23336 | 243.1602 | C13H24O4 | Long-chain fatty acids |
| NCGC00169620-03_C30H48O6_Olean-12-en-28-oic acid, 2,3,19,23-tetrahydroxy-, (2alpha,3beta,5xi,9xi,19alpha)- | 17.52 | 549.34581 | 8528 | 549.34302 | C30H48O6 | Triterpenoids |
| (Z)-9,12,13-trihydroxyoctadec-15-enoic acid | 17.14 | 329.23377 | 1118800 | 329.23621 | C18H34O5 | Long-chain fatty acids |
| (3Z,5E)-4-hydroxy-6-(4-hydroxyphenyl)hexa-3,5-dien-2-one | 16.90 | 203.07239 | 23018 | 203.07137 | C12H12O3 | Styrenes |
| 4-tert-Butylbenzoic acid | 16.87 | 177.09283 | 16783 | 177.0921 | C11H14O2 | Phenylpropanes |
| MONENSIN | 16.86 | 669.42388 | 6070 | 669.42291 | C36H62O11 | Heterocyclic fatty acids |
| 1-(2,4-dihydroxyphenyl)-2-(3,5-dimethoxyphenyl)propan-1-one | 16.84 | 301.10932 | 10677 | 301.10815 | C17H18O5 | Alpha-methyldeoxybenzoin flavonoids |
| AKOS040734751 | 16.79 | 411.10978 | 76538 | 411.10855 | C22H20O8 | 4-prenylated xanthones |
| Pechueloic Acid | 16.72 | 247.13504 | 16678 | 247.134 | C15H20O3 |  |
| matairesinol | 16.60 | 357.13609 | 14023 | 357.13437 | C20H22O6 | Dibenzylbutyrolactone lignans |
| 6-[3-(3,4-dimethoxyphenyl)-1,3,3a,4,6,6a-hexahydrofuro[3,4-c]furan-6-yl]-4-methoxy-1,3-benzodioxole | 16.54 | 399.14642 | 80918 | 399.14493 | C22H24O7 | Furanoid lignans |
| 4-hydroxy-3-(3-methylbut-2-enyl)benzoic acid | 16.51 | 205.08743 | 1670642 | 205.08702 | C12H14O3 | Hydroxybenzoic acid derivatives |
| Cohumulone | 16.46 | 347.18766 | 39688 | 347.18619 | C20H28O5 | Acyloins |
| 3',4',7-Trihydroxyflavanone | 16.41 | 271.06234 | 15815 | 271.06119 | C15H12O5 |  |
| FA 18:2+3O | 16.31 | 327.21835 | 117190 | 327.21649 | C18H32O5 | Oxidized fatty acids |
| 5,7-Dihydroxy-2,6,8-trimethylchromone | 16.31 | 219.06729 | 9358 | 219.065 | C12H12O4 | Chromones |
| Dodecanedioic acid | 16.16 | 229.14532 | 11667 | 229.1445 | C12H22O4 | Medium-chain fatty acids |
| Kanakugiol | 16.15 | 343.1203 | 18571 | 343.1185 | C19H20O6 |  |
| Sulfamethoxazole | 16.02 | 252.04347 | 24724 | 252.0448 | C10H11N3O3S | Aminobenzenesulfonamides |
| Formononetin * | 16.02 | 267.06657 | 2277623 | 267.06628 | C16H12O4 | 4'-O-methylisoflavones |
| NCGC00380146-01_C20H28O5_ | 15.94 | 347.18885 | 8000 | 347.186 | C20H28O5 | Diterpene lactones |
| 1-[4-hydroxy-3-(3-methylbut-2-enyl)phenyl]ethanone | 15.90 | 203.10921 | 14499 | 203.10774 | C13H16O2 | Alkyl-phenylketones |
| (Z)-5,8,11-trihydroxyoctadec-9-enoic acid | 15.87 | 329.23362 | 5167677 | 329.2334 | C18H34O5 | Long-chain fatty acids |
| Arnicolide D | 15.81 | 331.1559 | 365118 | 331.15509 | C19H24O5 | Sesquiterpene lactones |
| angoletin | 15.77 | 299.13102 | 11931 | 299.12891 | C18H20O4 |  |
| NCGC00384722-02!5-hydroxy-2-(3-hydroxy-4-methoxyphenyl)-7-[(2S,3R,4S,5S,6R)-3,4,5-trihydroxy-6-[[(2R,3R,4R,5R,6S)-3,4,5-trihydroxy-6-methyloxan-2-yl]oxymethyl]oxan-2-yl]oxy-2,3-dihydrochromen-4-one | 15.18 | 655.1835 | 23218 | 655.18799 | C28H34O15 | Flavonoid-7-O-glycosides |
| Mycophenolic Acid | 14.95 | 319.12057 | 9114 | 319.1188 | C17H20O6 |  |
| (10E,15Z)-9,12,13-trihydroxyoctadeca-10,15-dienoic acid | 14.91 | 327.21817 | 950421 | 327.2178 | C18H32O5 | Lineolic acids and derivatives |
| Koparin | 14.83 | 299.0571 | 27922 | 299.05612 | C16H12O6 | 3'-hydroxy,4'-methoxyisoflavonoids |
| Dipteryxin | 14.60 | 313.07272 | 56063 | 313.07175 | C17H14O6 | 4'-O-methylisoflavones |
| Dalbergione, 4-Methoxy-4'-Hydroxy- | 14.51 | 269.08287 | 27692 | 269.08191 | C16H14O4 | Dalbergiones |
| Undecanedioic acid | 14.39 | 215.12965 | 16629 | 215.12891 | C11H20O4 | Medium-chain fatty acids |
| (2E)-2-[hydroxy(phenyl)methylidene]-6-methoxy-1-benzofuran-3-one | 14.31 | 267.06734 | 5647 | 267.06628 | C16H12O4 | Aurone flavonoids |
| lucidin 3-O-beta-primveroside | 14.19 | 563.12319 | 6629 | 563.12073 | C26H28O14 | Anthraquinones |
| NCGC00169221-01 | 14.14 | 629.18815 | 1261201 | 629.18707 | C30H32O12 | Terpene glycosides |
| Procyanidin B1 (isomer of 1574); PlaSMA ID-1575 | 14.02 | 577.13549 | 442348 | 577.13483 | C30H26O12 | Biflavonoids |
| NCGC00380901-01_C23H24O10S_ | 13.88 | 491.09919 | 138525 | 491.10199 | C23H24O10S | 1-hydroxy-2-unsubstituted benzenoids |
| Naringenin | 13.78 | 271.06234 | 20767 | 271.06119 | C15H12O5 |  |
| FA 18:2+4O | 13.73 | 343.21387 | 24147 | 343.21219 | C18H32O6 | Long-chain fatty acids |
| (E)-1,4-bis(4-hydroxy-3-methoxyphenyl)-2,3-dimethylbut-2-ene-1,4-dione | 13.69 | 355.1196 | 49955 | 355.11871 | C20H20O6 | Lignans, neolignans and related compounds |
| Goniothalenol | 13.69 | 231.06752 | 7318 | 231.06628 | C13H12O4 | Furopyrans |
| Sappanone A Dimethyl Ether | 13.35 | 311.0939 | 10116 | 311.0925 | C18H16O5 | Homoisoflavonoids |
| NCGC00385222-01_C19H26O13_beta-D-Fructofuranosyl 6-O-(4-hydroxybenzoyl)-alpha-D-glucopyranoside | 13.34 | 507.13036 | 330637 | 507.13599 | C19H26O13 | O-glycosyl compounds |
| isorhapontin | 13.22 | 419.13536 | 5691 | 419.13474 | C21H24O9 | Stilbene glycosides |
| NCGC00381207-01 | 13.22 | 347.18717 | 7146 | 347.186 | C20H28O5 | Diterpene lactones |
| Bayogenin base + O-Hex-Hex | 13.15 | 857.45674 | 5073 | 857.45062 | C42H68O15 | Triterpene saponins |
| (2R,3S,4S,5R,6R)-2-[[(2R,3R,4R,5S)-3,4-dihydroxy-5-(hydroxymethyl)oxolan-2-yl]oxymethyl]-6-[(2E)-3,7-dimethylocta-2,6-dienoxy]oxane-3,4,5-triol | 12.94 | 447.22444 | 40115 | 447.22357 | C21H36O10 | Terpene glycosides |
| 2-[2-[(Z)-pent-2-enyl]-3-[3,4,5-trihydroxy-6-(hydroxymethyl)oxan-2-yl]oxycyclopentyl]acetic acid | 12.94 | 373.18797 | 45051 | 373.18677 | C18H30O8 | Fatty acyl glycosides of mono- and disaccharides |
| 2-(4-Methyl-3-cyclohexen-1-yl)-2-propanyl 6-O-(6-deoxy-alpha-L-mannopyranosyl)-beta-D-glucopyranoside | 12.93 | 507.24562 | 61034 | 507.245 | C22H38O10 | O-glycosyl compounds |
| 3-Hydroxy-N-(3-nitrophenyl)naphthalene-2-carboxamide | 12.92 | 307.07341 | 41446 | 307.07239 | C17H12N2O4 | Naphthalene-2-carboxanilides |
| FA 9:1+1O | 12.84 | 171.10343 | 28625 | 171.10181 | C9H16O3 | Medium-chain fatty acids |
| Acacetin | 12.80 | 283.0617 | 1358891 | 283.06219 | C16H12O5 |  |
| 2-acetyl-4-[(3-butanoyl-2,4,6-trihydroxy-5-methylphenyl)methyl]-3,5-dihydroxy-6,6-dimethylcyclohexa-2,4-dien-1-one | 12.79 | 463.16196 | 132666 | 463.16101 | C22H26O8 | Alkyl-phenylketones |
| 3-[5-(3,4-dihydroxy-4-methyl-5-oxooxolan-2-yl)-2-[(2S,3R,4S,5S,6R)-6-[3-[5-(3,4-dihydroxy-4-methyl-5-oxooxolan-2-yl)-4-methoxy-2-[(2S,3R,4S,5S,6R)-3,4,5-trihydroxy-6-(hydroxymethyl)oxan-2-yl]oxyphenyl]propanoyloxymethyl]-3,4,5-trihydroxyoxan-2-yl]oxy-4-methoxyphenyl]propanoic acid | 12.77 | 957.28475 | 12841 | 957.28815 | C42H54O25 | Phenolic glycosides |
| 5-Hydroxymethylcytidine | 12.50 | 272.09392 | 29152 | 272.08841 | C10H15N3O6 | Pyrimidine nucleosides |
| 3-(3,4-dihydroxyphenyl)-2-[(E)-3-[2-(3,4-dihydroxyphenyl)-7-hydroxy-3-methoxycarbonyl-2,3-dihydro-1-benzofuran-4-yl]prop-2-enoyl]oxypropanoic acid | 12.40 | 551.12072 | 20256 | 551.12 | C28H24O12 | 2-arylbenzofuran flavonoids |
| Isoliquiritigenin | 12.01 | 255.06827 | 5530 | 255.0663 | C15H12O4 |  |
| santin | 11.78 | 343.08355 | 19512 | 343.08234 | C18H16O7 | 6-O-methylated flavonoids |
| Isopeonol | 11.74 | 165.05676 | 8498 | 165.05571 | C9H10O3 | Alkyl-phenylketones |
| BUTYL PARABEN | 11.67 | 193.08771 | 259467 | 193.08702 | C11H14O3 | p-Hydroxybenzoic acid alkyl esters |
| Formononetin-7-O-glucoside * | 11.61 | 475.12491 | 650720 | 475.12457 | C22H22O9 | Isoflavonoid O-glycosides |
| Scutellarioside II | 11.37 | 507.15069 | 299259 | 507.15079 | C24H28O12 |  |
| (2R)-2-[(E)-3-[3-[(1R)-1-carboxy-2-(3,4-dihydroxyphenyl)ethoxy]carbonyl-2-(3,4-dihydroxyphenyl)-7-hydroxy-2,3-dihydro-1-benzofuran-4-yl]prop-2-enoyl]oxy-3-(3,4-dihydroxyphenyl)propanoic acid | 11.20 | 717.145 | 11827600 | 717.146 | C36H30O16 | 2-arylbenzofuran flavonoids |
| Irisxanthone | 11.10 | 435.0947 | 12110 | 435.09439 | C20H20O11 |  |
| FA 9:0+1O | 11.04 | 173.11875 | 78249 | 173.11819 | C9H18O3 | Medium-chain hydroxy acids and derivatives |
| 2-(4-hydroxyphenyl)ethyl (1S,4aR,7aR)-4a-hydroxy-7-methyl-5-oxo-1-[(2S,3R,4S,5S,6R)-3,4,5-trihydroxy-6-(hydroxymethyl)oxan-2-yl]oxy-1,6,7,7a-tetrahydrocyclopenta[c]pyran-4-carboxylate | 10.93 | 509.16673 | 97586 | 509.16644 | C24H30O12 | Iridoid O-glycosides |
| Myricetin | 10.87 | 315.01458 | 348003 | 315.01199 | C15H10O8 | Flavonols |
| Albiflorin * | 10.85 | 479.15704 | 42605 | 479.15588 | C23H28O11 | Terpene glycosides |
| Methyl 7-Deshydroxypyrogallin-4-Carboxylate | 10.64 | 261.04219 | 5398 | 261.04044 | C13H10O6 | Tropolones |
| Umbelliferone | 10.57 | 161.02546 | 81753 | 161.02431 | C9H6O3 |  |
| 2-[2-[1-carboxy-2-(3,4-dihydroxyphenyl)ethoxy]carbonyl-3,4-bis(3,4-dihydroxyphenyl)cyclobutanecarbonyl]oxy-3-(3,4-dihydroxyphenyl)propanoic acid | 10.57 | 719.1622 | 2463486 | 719.16199 | C36H32O16 | Cyclobutane lignans |
| Rosmarinic acid * | 10.57 | 359.07802 | 4262733 | 359.07724 | C18H16O8 | Coumaric acids and derivatives |
| 4-(3,4-dihydroxyphenyl)-7-methoxy-5-[(2S,3R,4S,5S,6R)-3,4,5-trihydroxy-6-(hydroxymethyl)oxan-2-yl]oxychromen-2-one | 10.49 | 507.11516 | 23228 | 507.11401 | C22H22O11 | Coumarin glycosides |
| (2S,3R,4S,5S,6R)-2-[4-[(3S,3aR,6S,6aR)-3-(4-hydroxy-3,5-dimethoxyphenyl)-1,3,3a,4,6,6a-hexahydrofuro[3,4-c]furan-6-yl]-2,6-dimethoxyphenoxy]-6-(hydroxymethyl)oxane-3,4,5-triol | 10.45 | 579.20854 | 41487 | 579.20898 | C28H36O13 | Lignan glycosides |
| (1S,3R,4R,5R)-3,4-bis[[(E)-3-(3,4-dihydroxyphenyl)prop-2-enoyl]oxy]-1,5-dihydroxycyclohexane-1-carboxylic acid | 10.42 | 515.12013 | 91878 | 515.12 | C25H24O12 | Quinic acids and derivatives |
| [4-[3,4,5-trihydroxy-6-(hydroxymethyl)oxan-2-yl]oxyphenyl]methyl 3-acetyloxy-2-hydroxy-2-[(4-hydroxyphenyl)methyl]butanoate | 10.28 | 535.18251 | 11742 | 535.18207 | C26H32O12 | Phenolic glycosides |
| Salicylic acid | 10.03 | 137.02461 | 1137327 | 137.0244 | C7H6O3 | Salicylic acids |
| (2R,3S,4S,5R,6R)-5-[(2S,3R,4R)-3,4-dihydroxy-4-(hydroxymethyl)oxolan-2-yl]oxy-2-(hydroxymethyl)-6-[2-(4-hydroxyphenyl)ethoxy]oxane-3,4-diol | 9.98 | 431.15715 | 8136 | 431.15588 | C19H28O11 | O-glycosyl compounds |
| NCGC00385031-01!4-[(2S,3R,4S,5S,6R)-6-[(3,4-dihydroxybenzoyl)oxymethyl]-3,4,5-trihydroxyoxan-2-yl]oxy-3-methoxybenzoic acid | 9.97 | 465.10609 | 7681 | 465.104 | C21H22O12 | Hydrolyzable tannins |
| RESORCINOL | 9.88 | 151.04142 | 14979 | 151.03999 | C8H8O3 | Phenol esters |
| 2-(4-hydroxyphenyl)-7-[(2S,3R,4S,5S,6R)-3,4,5-trihydroxy-6-(hydroxymethyl)oxan-2-yl]oxy-2,3-dihydrochromen-4-one | 9.83 | 417.08185 | 7342221 | 417.0838 | C21H22O9 | Flavonoid-7-O-glycosides |
| (1R,3R,4S,5R)-3,4-bis[[(E)-3-(3,4-dihydroxyphenyl)prop-2-enoyl]oxy]-1,5-dihydroxycyclohexane-1-carboxylic acid | 9.68 | 515.11998 | 50878 | 515.12 | C25H24O12 | Quinic acids and derivatives |
| Indole-3-carboxyaldehyde | 9.64 | 144.04609 | 24012 | 144.04494 | C9H7NO | Indoles |
| Theaflavin Monogallates | 9.60 | 715.13078 | 95926 | 715.13049 | C36H28O16 | Catechins |
| salviaflaside | 9.47 | 521.13048 | 108266 | 521.13007 | C24H26O13 | Phenolic glycosides |
| Hydroxysebacic acid; PlaSMA ID-308 | 9.45 | 217.10869 | 31161 | 217.10789 | C10H18O5 | Organic acids |
| (3S,5R,8S)-2-(benzoyloxymethyl)-8-methyl-3-[(2S,3R,4S,5S,6R)-3,4,5-trihydroxy-6-(hydroxymethyl)oxan-2-yl]oxy-9,10-dioxatetracyclo[4.3.1.02,5.03,8]decane-6-sulfonic acid | 9.32 | 543.11477 | 165724 | 543.1178 | C23H28O13S | Terpene glycosides |
| 4-[(E)-3-[1-carboxy-2-(3,4-dihydroxyphenyl)ethoxy]-3-oxoprop-1-enyl]-2-(3,4-dihydroxyphenyl)-7-hydroxy-2,3-dihydro-1-benzofuran-3-carboxylic acid | 9.17 | 537.10359 | 1348024 | 537.10382 | C27H22O12 | 2-arylbenzofuran flavonoids |
| Sissotrin * | 9.12 | 491.11936 | 716592 | 491.12 | C22H22O10 | Isoflavonoid O-glycosides |
| Biochanin A * | 9.12 | 283.06229 | 49517 | 283.06119 | C16H12O5 | 4'-O-methylisoflavones |
| calceolarioside A | 9.06 | 477.14076 | 20298 | 477.1402 | C23H26O11 |  |
| barceloneic acid A | 9.06 | 319.08319 | 19999 | 319.08234 | C16H16O7 | Diphenylethers |
| Malonyltryptophan | 8.96 | 289.08453 | 7409 | 289.0813 | C14H14N2O5 | N-acyl-alpha amino acids |
| N-acetyltryptophan | 8.96 | 245.0943 | 15240 | 245.09315 | C13H14N2O3 | N-acyl-alpha amino acids |
| isoferulic acid | 8.96 | 193.05124 | 177807 | 193.05063 | C10H10O4 | Hydroxycinnamic acids |
| Ellagic Acid | 8.96 | 300.99985 | 186974 | 300.99902 | C14H6O8 | Hydrolyzable tannins |
| NCGC00384596-01 | 8.92 | 537.10352 | 2211678 | 537.104 | C27H22O12 | 2-arylbenzofuran flavonoids |
| Anhydrobrazilic Acid | 8.89 | 233.04662 | 39048 | 233.04555 | C12H10O5 | Chromones |
| N-acetylphenylalanine | 8.82 | 206.08293 | 16594 | 206.08226 | C11H13NO3 | Phenylalanine and derivatives |
| SINAPIC ACID | 8.70 | 223.06146 | 6576 | 223.06119 | C11H12O5 | Hydroxycinnamic acids |
| N-Hydroxybenzamide | 8.70 | 136.04129 | 7963 | 136.04041 | C7H7NO2 | Benzoic acids and derivatives |
| [(2S,3R)-2-(3,4-dihydroxyphenyl)-3,5-dihydroxy-3,4-dihydro-2H-chromen-7-yl] 3,4,5-trihydroxybenzoate | 8.68 | 441.08392 | 10591 | 441.0827 | C22H18O10 | Catechin gallates |
| NCGC00091914-04!(2R,3R)-2,3-bis[[(E)-3-(3,4-dihydroxyphenyl)prop-2-enoyl]oxy]butanedioic acid | 8.62 | 473.07424 | 10884 | 473.073 | C22H18O12 | Tetracarboxylic acids and derivatives |
| SUBERIC ACID | 8.59 | 173.08243 | 20564 | 173.08099 | C8H14O4 | Medium-chain fatty acids |
| Porphyrilic acid | 8.44 | 313.0352 | 5835 | 313.035 | C16H10O7 | Dibenzofurans |
| 3-phenyllactic acid | 8.41 | 165.05656 | 18502 | 165.05571 | C9H10O3 | Phenylpropanoic acids |
| (2S,3R,4S,5S,6R)-2-[4-[(3R,4S)-4-hydroxy-4-[(4-hydroxy-3-methoxyphenyl)methyl]-3-(hydroxymethyl)oxolan-2-yl]-2-methoxyphenoxy]-6-(hydroxymethyl)oxane-3,4,5-triol | 8.31 | 537.19926 | 7896 | 537.19775 | C26H34O12 | Lignan glycosides |
| Evodiamine | 8.30 | 302.12644 | 7548 | 302.12988 | C19H17N3O | Beta carbolines |
| methyl (3R,4R,5R)-5-[(2R,3R,4S,5S,6R)-4,5-dihydroxy-6-(hydroxymethyl)-3-[(E)-3-phenylprop-2-enoyl]oxyoxan-2-yl]oxy-3,4-dihydroxycyclohexene-1-carboxylate | 8.21 | 515.1339 | 26474 | 515.13171 | C23H28O11 | Fatty acyl glycosides of mono- and disaccharides |
| Paeoniflorin * | 8.18 | 525.16112 | 8944552 | 525.16138 | C23H28O11 | Terpene glycosides |
| [(1S,2S,3R,5R,6S,8S)-6-hydroxy-8-methyl-3-{[(2S,3R,4S,5S,6R)-3,4,5-trihydroxy-6-(hydroxymethyl)oxan-2-yl]oxy}-9,10-dioxatetracyclo[4.3.1.0?,?.0?,?]decan-2-yl]methyl benzoate | 8.18 | 479.15695 | 276414 | 479.15588 | C23H28O11 | Terpene glycosides |
| Hydroxysuberic acid | 8.08 | 189.07697 | 266892 | 189.0766 | C8H14O5 | Medium-chain hydroxy acids and derivatives |
| Galangin | 8.07 | 269.04644 | 6763 | 269.0455 | C15H10O5 |  |
| 4-Vinylphenol | 8.05 | 119.05064 | 13889 | 119.0491 | C8H8O | Styrenes |
| 4-COUMARATE | 8.05 | 163.04048 | 39454 | 163.03999 | C9H8O3 | Hydroxycinnamic acids |
| 3-O-Feruloylquinic acid | 7.96 | 367.10367 | 92988 | 367.10345 | C17H20O9 | Quinic acids and derivatives |
| (2S)-6-[(2S)-5,7-dihydroxy-2-(4-hydroxyphenyl)-4-oxo-2,3-dihydrochromen-8-yl]-5,7-dihydroxy-2-(4-hydroxyphenyl)-2,3-dihydrochromen-4-one | 7.96 | 541.15712 | 19439 | 541.15686 | C30H22O10 | Biflavonoids and polyflavonoids |
| (2S,3S,4S,5R,6R)-6-(3-benzoyloxy-2-hydroxypropoxy)-3,4,5-trihydroxyoxane-2-carboxylic acid | 7.93 | 371.09861 | 67193 | 371.09836 | C16H20O10 | O-glucuronides |
| 1-Methoxy-3-carbaldehyde | 7.92 | 174.05678 | 16055 | 174.05551 | C10H9NO2 | Indoles |
| [(2S,3R,4S,5S,6R)-3,4,5-trihydroxy-6-(hydroxymethyl)oxan-2-yl] (2E,6E)-8-hydroxy-2,6-dimethylocta-2,6-dienoate | 7.89 | 345.15574 | 113174 | 345.15549 | C16H26O8 | Terpene glycosides |
| 4-Hydroxyacetophenone | 7.87 | 135.04586 | 21425 | 135.0452 | C8H8O2 | Alkyl-phenylketones |
| NCGC00380875-01 | 7.86 | 505.19283 | 60451 | 505.19299 | C21H32O11 | Fatty acyl glycosides of mono- and disaccharides |
| (5Z)-4-[2-[2-(3,4-dihydroxyphenyl)ethoxy]-2-oxoethyl]-5-ethylidene-6-[(2S,3R,4S,5S,6R)-3,4,5-trihydroxy-6-(hydroxymethyl)oxan-2-yl]oxy-4H-pyran-3-carboxylic acid | 7.77 | 525.1619 | 759786 | 525.16138 | C24H30O13 | Terpene glycosides |
| 6-PHOSPHOGLUCONIC ACID | 7.75 | 275.02049 | 30857 | 275.017 | C6H13O10P | Monosaccharide phosphates |
| Feruloyl Lactate | 7.68 | 281.06726 | 49656 | 281.06479 | C13H14O7 | Coumaric acids and derivatives |
| (4S,5Z,6S)-4-(2-methoxy-2-oxoethyl)-5-[2-[(E)-3-phenylprop-2-enoyl]oxyethylidene]-6-[(2S,3R,4S,5S,6R)-3,4,5-trihydroxy-6-(hydroxymethyl)oxan-2-yl]oxy-4H-pyran-3-carboxylic acid | 7.65 | 149.06109 | 205210 | 149.06081 | C9H10O2 | Catechols |
| (2S,3R,4R,5R,6S)-2-[(2R,3R,4S,5R,6R)-2-[2-(3,4-dihydroxyphenyl)ethoxy]-3,5-dihydroxy-6-(hydroxymethyl)oxan-4-yl]oxy-6-methyloxane-3,4,5-triol | 7.64 | 461.16724 | 7520 | 461.16644 | C20H30O12 | O-glycosyl compounds |
| 4-hydroxy-3-[(4-hydroxy-6-methyl-2-oxopyran-3-yl)methyl]-6-methylpyran-2-one | 7.50 | 263.05654 | 28509 | 263.05612 | C13H12O6 | Pyranones and derivatives |
| N-Acetylleucine | 7.44 | 172.09831 | 23230 | 172.0999 | C8H15NO3 | Leucine and derivatives |
| 1,3,6-tri-O-galloylglucose | 7.38 | 635.0894 | 15581 | 635.08899 | C27H24O18 |  |
| 2-Hydroxy-4-methylpentanoic acid | 7.38 | 131.07174 | 22620 | 131.0713 | C6H12O3 | Hydroxy fatty acids |
| 1-(3,4-dihydroxyphenyl)-6,7-dihydroxy-1,2-dihydronaphthalene-2,3-dicarboxylic acid | 7.38 | 357.06203 | 56843 | 357.06158 | C18H14O8 | Aryltetralin lignans |
| 12:4+3O fatty acyl hexoside | 7.33 | 387.16637 | 8202 | 387.16501 | C18H28O9 | Fatty acyl glycosides of mono- and disaccharides |
| ginnalin A | 7.23 | 467.08348 | 20114 | 467.0831 | C20H20O13 | Galloyl esters |
| Phenylacetylaspartic acid | 7.20 | 250.07234 | 45818 | 250.0713 | C12H13NO5 | Amino acids |
| [(1aS,1bS,5S,5aS,6S)-6-hydroxy-6-(hydroxymethyl)-2-[[(2R,3R,4S,5S,6R)-3,4,5-trihydroxy-6-(hydroxymethyl)oxan-2-yl]oxymethyl]-1b,5,5a,6a-tetrahydro-1aH-oxireno[3,4]cyclopenta[1,3-d]pyran-5-yl] 3-methylbutanoate | 7.19 | 475.18204 | 14858 | 475.1821 | C21H32O12 | O-glycosyl compounds |
| Benzyl alcohol + Hex-Pen | 7.11 | 447.15075 | 53225 | 447.1517 | C18H26O10 | O-glycosyl compounds |
| 2-Hydroxy-3,4-Dimethoxybenzoic Acid | 7.08 | 197.04605 | 8563 | 197.0455 | C9H10O5 | P-methoxybenzoic acids and derivatives |
| Methoxytyrosine; PlaSMA ID-288 | 6.89 | 210.07709 | 215385 | 210.0766 | C10H13NO4 | Amino acids |
| fraxin | 6.88 | 369.08331 | 11852 | 369.0827 | C16H18O10 | Coumarin glycosides |
| 12:4+3O fatty acyl hexoside; PlaSMA ID-966 | 6.85 | 387.16605 | 9132 | 387.16501 | C18H28O9 | Fatty acyl hexosides |
| dihydroferulic acid | 6.74 | 195.0667 | 47351 | 195.06628 | C10H12O4 | Phenylpropanoic acids |
| 4-HYDROXYBENZALDEHYDE | 6.71 | 121.02974 | 32401 | 121.029 | C7H6O2 | Hydroxybenzaldehydes |
| PYROCATECHUIC ACID | 6.48 | 153.01996 | 40576 | 153.01933 | C7H6O4 | Salicylic acids |
| (1R,3R,4S,5R)-1,3,4-trihydroxy-5-[(E)-3-(4-hydroxy-3-methoxyphenyl)prop-2-enoyl]oxycyclohexane-1-carboxylic acid | 6.47 | 367.1039 | 26189 | 367.10345 | C17H20O9 | Quinic acids and derivatives |
| Caffeic acid | 6.46 | 179.03537 | 216523 | 179.03529 | C9H8O4 | Hydroxycinnamic acids |
| (1S,3R,4S,5R)-4-{[(2E)-3-(3,4-dihydroxyphenyl)prop-2-enoyl]oxy}-1,3,5-trihydroxycyclohexane-1-carboxylic acid | 6.29 | 353.08781 | 396652 | 353.0878 | C16H18O9 | Quinic acids and derivatives |
| 3,4-DIHYDROXYBENZOATE | 6.27 | 153.01953 | 143518 | 153.019 | C7H6O4 | Hydroxybenzoic acid derivatives |
| 4-ACETOXYPHENOL | 6.24 | 151.04049 | 20677 | 151.04007 | C8H8O3 | Phenol esters |
| reticulol | 6.20 | 221.04653 | 6349 | 221.04555 | C11H10O5 | Isocoumarins and derivatives |
| Uncarine c | 6.20 | 367.16149 | 11567 | 367.16632 | C21H24N2O4 | Indolizidines |
| Ferulic Acid * | 6.17 | 193.05139 | 27920 | 193.05063 | C10H10O4 | Hydroxycinnamic acids |
| D-(-)-Quinic acid | 6.05 | 191.0566 | 35101 | 191.05559 | C7H12O6 | Quinic acids and derivatives |
| Chlorogenic Acid | 6.05 | 353.08786 | 217236 | 353.0878 | C16H18O9 |  |
| 3-AMINO-5-HYDROXYBENZOIC ACID | 6.05 | 152.03586 | 45599 | 152.035 | C7H7NO3 | Hydroxybenzoic acid derivatives |
| [4,5,8-trihydroxy-5-(hydroxymethyl)-3-(3-methoxy-3-oxoprop-1-en-2-yl)-8a-methyl-1,2,3,4,4a,6,7,8-octahydronaphthalen-2-yl] 3,4-dihydroxy-2-methylidenebutanoate | 5.98 | 443.19194 | 179283 | 443.19226 | C21H32O10 | Eudesmane, isoeudesmane or cycloeudesmane sesquiterpenoids |
| hypericin | 5.91 | 503.14059 | 33364 | 503.1413 | C30H16O8 | Benzopyrenes |
| (Z)-3-[4-methoxy-2-[(2S,3R,4S,5S,6R)-3,4,5-trihydroxy-6-(hydroxymethyl)oxan-2-yl]oxyphenyl]prop-2-enoic acid | 5.82 | 355.10287 | 110734 | 355.10345 | C16H20O9 | Phenolic glycosides |
| NCGC00180844-02!(E)-3-[4-methoxy-2-[(2S,3R,4S,5S,6R)-3,4,5-trihydroxy-6-(hydroxymethyl)oxan-2-yl]oxyphenyl]prop-2-enoic acid | 5.82 | 401.10863 | 35312 | 401.10901 | C16H20O9 | Phenolic glycosides |
| Tetrasaccharides (Hex-Hex-Hex-Hex) | 5.82 | 711.21321 | 11249 | 711.21698 | C24H42O21 | Oligosaccharides |
| 3-[4-methoxy-2-[(2S,3R,4S,5S,6R)-3,4,5-trihydroxy-6-(hydroxymethyl)oxan-2-yl]oxyphenyl]propanoic acid | 5.73 | 357.11961 | 11275 | 357.11911 | C16H22O9 | Phenolic glycosides |
| Methylgallate | 5.72 | 183.03042 | 20222 | 183.03329 | C8H8O5 | Galloyl esters |
| (2R)-3-(3,4-dihydroxyphenyl)-1-[2,4-dihydroxy-3-[(2S,3R,4R,5S,6R)-3,4,5-trihydroxy-6-(hydroxymethyl)oxan-2-yl]phenyl]-2-hydroxypropan-1-one | 5.64 | 451.12511 | 10315 | 451.12457 | C21H24O11 | 2'-Hydroxy-dihydrochalcones |
| NCGC00381247-01!3-ethenyl-4-[2-(3,4,5-trihydroxybenzoyl)oxyethyl]-2-[3,4,5-trihydroxy-6-(hydroxymethyl)oxan-2-yl]oxy-3,4-dihydro-2H-pyran-5-carboxylic acid | 5.61 | 527.14063 | 71467 | 527.14099 | C23H28O14 | Terpene glycosides |
| Coumarin + 1O + 1MeO, O-Hex-Hex | 5.59 | 515.14073 | 65513 | 515.14319 | C22H28O14 | Coumarin and derivatives |
| 2-Isopropylmalic acid | 5.55 | 175.06116 | 886605 | 175.06067 | C7H12O5 | Hydroxy fatty acids |
| 3-[(1-Carboxyvinyl)oxy]benzoic acid | 5.45 | 207.03013 | 59814 | 207.03044 | C10H8O5 | Phenoxyacetic acid derivatives |
| [3,4,5-trihydroxy-6-(3,4,5-trihydroxybenzoyl)oxyoxan-2-yl]methyl 3,4,5-trihydroxybenzoate | 5.43 | 483.07822 | 16545 | 483.07803 | C20H20O14 | Tannins |
| NCGC00169476-02 | 5.37 | 529.15615 | 27227 | 529.15601 | C22H28O12 | Coumaric acids and derivatives |
| 4-hydroxyphenylacetic acid | 5.26 | 151.04038 | 18046 | 151.04007 | C8H8O3 | 1-hydroxy-2-unsubstituted benzenoids |
| (2R,3S,4S,5R,6S)-2-(hydroxymethyl)-6-(3,4,5-trimethoxyphenoxy)oxane-3,4,5-triol | 5.15 | 345.11966 | 10114 | 345.11911 | C15H22O9 | Phenolic glycosides |
| rhodioloside | 5.15 | 299.11414 | 9101 | 299.11362 | C14H20O7 | O-glycosyl compounds |
| Protocatechuic aldehyde | 4.96 | 137.02408 | 8766388 | 137.0239 | C7H6O3 | Hydroxybenzaldehydes |
| [(2R,3S,4S,5R,6R)-6-[(2S,3S,4S,5R)-3,4-dihydroxy-2,5-bis(hydroxymethyl)oxolan-2-yl]oxy-3,4,5-trihydroxyoxan-2-yl]methyl 4-hydroxybenzoate | 4.81 | 461.13035 | 85279 | 461.13007 | C19H26O13 | O-glycosyl compounds |
| Proline-hydroxyproline | 4.76 | 227.10393 | 28094 | 227.10339 | C10H16N2O4 | Dipeptides |
| Caffeic acid hexoside | 4.74 | 341.08833 | 51769 | 341.0871 | C15H18O9 | Hydroxycinnamic acid glycosides |
| Aspirin | 4.74 | 179.03569 | 11103 | 179.035 | C9H8O4 | Acylsalicylic acids |
| Pidotimod | 4.69 | 243.04459 | 263584 | 243.0426 | C9H12N2O4S | Dipeptides |
| Biotin | 4.69 | 487.09699 | 14395 | 487.09854 | C10H16N2O3S | Biotin and derivatives |
| Hydroxysuberic acid | 4.65 | 189.07741 | 7133 | 189.0766 | C8H14O5 | Organic acids |
| N-Methylglutamic acid | 4.61 | 160.06158 | 307567 | 160.06171 | C6H11NO4 | Glutamic acid and derivatives |
| (Z)-2-methyl-4-[(2R,3R,4S,5S,6R)-3,4,5-trihydroxy-6-(hydroxymethyl)oxan-2-yl]oxybut-2-enenitrile | 4.57 | 258.0989 | 16441 | 258.0983 | C11H17NO6 | Fatty acyl glycosides of mono- and disaccharides |
| 3-(4-Hydroxyphenyl)lactic acid | 4.53 | 181.05093 | 323447 | 181.052 | C9H10O4 | Phenylpropanoic acids |
| (2R,3S,4S,5R,6S)-2-[[(2S,3R,4R)-3,4-dihydroxy-4-(hydroxymethyl)oxolan-2-yl]oxymethyl]-6-(3,4,5-trimethoxyphenoxy)oxane-3,4,5-triol | 4.50 | 477.16177 | 9372 | 477.16135 | C20H30O13 | Phenolic glycosides |
| (1R,21S,23R)-6,7,8,11,12,13,22,23-octahydroxy-3,16-dioxo-2,17,20-trioxatetracyclo[17.3.1.0?,?.0??,??]tricosa-4(9),5,7,10(15),11,13-hexaen-21-yl 3,4,5-trihydroxybenzoate | 4.39 | 633.07346 | 10771 | 633.07336 | C27H22O18 | Hydrolyzable tannins |
| (1r,3R,4s,5S)-4-{[(2E)-3-(3,4-Dihydroxyphenyl)-2-propenoyl]oxy}-1,3,5-trihydroxycyclohexanecarboxylic acid | 4.35 | 353.08725 | 822018 | 353.0878 | C16H18O9 | Quinic acids and derivatives |
| TRYPTOPHAN | 4.28 | 203.08254 | 129316 | 203.0826 | C11H12N2O2 | Indolyl carboxylic acids and derivatives |
| Oryzalin | 4.25 | 345.08281 | 27058 | 345.0874 | C12H18N4O6S | Dinitroanilines |
| 4-METHYLCATECHOL | 4.21 | 123.04522 | 19507 | 123.045 | C7H8O2 | Catechols |
| 3-Hydroxyvaleric acid | 4.04 | 117.05612 | 12455 | 117.0545 | C5H10O3 | Hydroxy fatty acids |
| Haematommic acid | 3.85 | 195.03037 | 13057 | 195.02901 | C9H8O5 | Hydroxybenzoic acid derivatives |
| Benzoic acid + 2O, O-Hex | 3.58 | 315.0718 | 896497 | 315.07001 | C13H16O9 | Phenolic glycosides |
| Coniferyl aldehyde * | 3.56 | 177.05607 | 18704 | 177.05573 | C10H10O3 | Methoxyphenols |
| methyl (1S,4aR,7aR)-4a-hydroxy-7-(hydroxymethyl)-1-[(2S,3R,4S,5S,6R)-3,4,5-trihydroxy-6-(hydroxymethyl)oxan-2-yl]oxy-5,7a-dihydro-1H-cyclopenta[c]pyran-4-carboxylate | 3.55 | 403.12482 | 18609 | 403.12457 | C17H24O11 | Iridoid O-glycosides |
| 2,3-DIHYDROXYBENZOATE | 3.26 | 153.01944 | 279406 | 153.019 | C7H6O4 | Salicylic acids |
| Catechol | 3.26 | 109.02981 | 44983 | 109.02898 | C6H6O2 | Catechols |
| Benzoic acid + 1O, 1MeO, O-Hex; PlaSMA ID-733 | 3.16 | 329.08805 | 25858 | 329.08841 | C14H18O9 | Phenolic glycosides |
| Pantothenate | 3.06 | 218.10382 | 20411 | 218.10339 | C9H17NO5 | Secondary alcohols |
| Piscidic Acid | 2.92 | 255.05109 | 18707 | 255.05099 | C11H12O7 | Phenylpropanoic acids |
| PHENYLACETIC ACID | 2.77 | 135.0457 | 183730 | 135.045 | C8H8O2 | Benzene and substituted derivatives |
| !methyl 2-(4a,6,10a-trihydroxy-1-methyl-5,10-dioxo-3,4-dihydro-1H-benzo[g]isochromen-3-yl)acetate | 2.76 | 395.09741 | 13982587 | 395.09799 | C17H18O8 | Naphthopyranones |
| Benzoic acid + 2O, O-Hex | 2.68 | 315.0719 | 94880 | 315.07089 | C13H16O9 | Phenolic glycosides |
| Glutaric acid | 2.68 | 131.03516 | 22448 | 131.03419 | C5H8O4 | Dicarboxylic acids and derivatives |
| Hydroquinone | 2.53 | 109.03 | 9740 | 109.0295 | C6H6O2 | Hydroquinones |
| Vanillic acid + O-sulfonateHex | 2.38 | 409.04476 | 11344 | 409.04419 | C14H18O12S | Sulfate containing metabolites |
| Phenylalanine | 2.38 | 164.07187 | 87451 | 164.07001 | C9H11NO2 | Phenylalanine and derivatives |
| (1S,3R,4S,5R)-1,3,4,5-tetrahydroxycyclohexane-1-carboxylic acid | 2.37 | 191.05605 | 526148 | 191.05611 | C7H12O6 | Quinic acids and derivatives |
| Melezitose | 2.30 | 503.16136 | 28986 | 503.16174 | C18H32O16 | Oligosaccharides |
| Pyrogallol | 2.14 | 125.0243 | 244601 | 125.0249 | C6H6O3 | 5-unsubstituted pyrrogallols |
| Kojic Acid | 2.07 | 141.01983 | 17762 | 141.01933 | C6H6O4 | Pyranones and derivatives |
| ASCORBIC ACID | 2.06 | 175.0254 | 13234 | 175.02481 | C6H8O6 | Butenolides |
| Shanzhiside methyl ester | 1.98 | 405.1394 | 181860 | 405.14023 | C17H26O11 | Iridoid O-glycosides |
| alpha-Hydroxyhippuric acid | 1.88 | 194.04625 | 35729 | 194.0459 | C9H9NO4 | Hippuric acids |
| Mesaconic acid | 1.81 | 129.01969 | 70766 | 129.01881 | C5H6O4 | Methyl-branched fatty acids |
| Gallic acid | 1.71 | 169.01414 | 5467383 | 169.0152 | C7H6O5 | Gallic acids |
| Larixinic Acid | 1.69 | 125.0248 | 230381 | 125.02441 | C6H6O3 | Pyranones and derivatives |
| 3,4-Dihydroxyphenylacetic acid | 1.69 | 166.99836 | 15155 | 167 | C8H8O4 | Catechols |
| (1S,4aS,7S,7aS)-7-hydroxy-7-methyl-1-[(2S,3R,4S,5S,6R)-3,4,5-trihydroxy-6-(hydroxymethyl)oxan-2-yl]oxy-4a,5,6,7a-tetrahydro-1H-cyclopenta[c]pyran-4-carboxylic acid | 1.63 | 375.12958 | 12977 | 375.12967 | C16H24O10 | Iridoid O-glycosides |
| 4-Pyridoxic acid | 1.56 | 182.04608 | 8898 | 182.04671 | C8H9NO4 | Pyridinecarboxylic acids |
| Meglutol | 1.51 | 161.04579 | 508951 | 161.04555 | C6H10O5 | Hydroxy fatty acids |
| N-Fructosyl isoleucine | 1.50 | 292.14016 | 182364 | 292.1391 | C12H23NO7 | Isoleucine and derivatives |
| L-tert-Leucine | 1.48 | 130.08749 | 28566 | 130.0874 | C6H13NO2 | Valine and derivatives |
| Citrazinc acid | 1.43 | 154.0149 | 7773 | 154.0146 | C6H5NO4 | Pyridinecarboxylic acids |
| 3-oxo-C4-homoserine lactone | 1.43 | 184.06174 | 13129 | 184.06149 | C8H11NO4 | N-acyl-alpha amino acids and derivatives |
| C6H9NO3; PlaSMA ID-80 | 1.40 | 142.05133 | 11619 | 142.0504 | C6H9NO3 | Formula predicted |
| CITRAMALATE | 1.38 | 147.03021 | 33331 | 147.02873 | C5H8O5 | Hydroxy fatty acids |
| Succinic acid | 1.36 | 117.01917 | 440775 | 117.0181 | C4H6O4 | Dicarboxylic acids and derivatives |
| Guanosine | 1.31 | 282.08439 | 46138 | 282.08386 | C10H13N5O5 | Purine nucleosides |
| N-Acetylglutamic acid | 1.27 | 188.0566 | 49651 | 188.05589 | C7H11NO5 | Glutamic acid and derivatives |
| Pyroglutamic acid | 1.23 | 128.03501 | 1045849 | 128.0343 | C5H7NO3 | Alpha amino acids and derivatives |
| 6-Hydroxynicotinic acid | 1.22 | 138.01987 | 68311 | 138.0186 | C6H5NO3 | Pyridinecarboxylic acids |
| 3-Hydroxypyridine | 1.22 | 94.03011 | 26247 | 94.02931 | C5H5NO | Hydroxypyridines |
| Aconitic Acid | 1.21 | 173.00926 | 50255 | 173.00916 | C6H6O6 | Tricarboxylic acids and derivatives |
| Arabinofuranosyluracil | 1.21 | 243.06304 | 18932 | 243.06226 | C9H12N2O6 | Pyrimidine nucleosides |
| N-Fructosyl pyroglutamate | 1.21 | 290.08813 | 262695 | 290.08719 | C11H17NO8 | N-Fructosyl amino acids |
| DL-Lactic acid | 1.04 | 89.02478 | 69288 | 89.02389 | C3H6O3 | Alpha hydroxy acids and derivatives |
| 2',4',6'-Trihydroxydihydrochalcone | 1.02 | 257.07783 | 19370 | 257.08191 | C15H14O4 |  |
| Citric acid | 0.95 | 191.01932 | 845550 | 191.0197 | C6H8O7 | Tricarboxylic acids and derivatives |
| 2-Furoic acid | 0.95 | 111.00899 | 75348 | 111.0077 | C5H4O3 | Furoic acids |
| MALATE | 0.93 | 133.01421 | 998064 | 133.01401 | C4H6O5 | Beta hydroxy acids and derivatives |
| Gluconic acid | 0.80 | 195.05109 | 40183 | 195.0508 | C6H12O7 | Medium-chain hydroxy acids and derivatives |
| Tetrasaccharides (Hex-Hex-Hex-Hex | 0.79 | 665.21302 | 91854 | 665.21252 | C24H42O21 | Sugars |
| maltotriose | 0.77 | 503.1608 | 101244 | 503.1618 | C18H32O16 |  |
| PFSA-Clsubstituted | 0.68 | 614.90029 | 7611 | 614.89429 | C10HClF20O3S | Organosulfonic acids |

## **2.3 Supplementary Table 3. Chemical annotation of QBHF-related MS features in traditional Chinese medicines (positive-ion mode).**

| Name | RT (min) | Precursor m/z | Area | Reference m/z | Formula | Ontology |
| --- | --- | --- | --- | --- | --- | --- |
| Xanthyletin | 11.872 | 251.07114 | 119952 | 251.07001 | C14H12O3 | Linear pyranocoumarins |
| Xanthotoxol | 10.746 | 203.03454 | 65043 | 203.035 | C11H6O4 | 8-hydroxypsoralens |
| Xanthone | 11.5 | 197.06123 | 9064 | 197.05969 | C13H8O2 | Xanthones |
| Viramune | 11.018 | 267.12133 | 6723 | 267.1235 | C15H14N4O | Alkyldiarylamines |
| Veraguensin | 5.094 | 411.15551 | 31237 | 411.159 | C22H28O5 | 7,7'-epoxylignans |
| Vanillin | 6.947 | 153.05458 | 117040 | 153.05499 | C8H8O3 | Methoxyphenols |
| ValylIsoleucine | 2.192 | 231.17045 | 151481 | 231.1714 | C11H22N2O3 | Dipeptides |
| Valerophenone | 13.934 | 163.11189 | 107895 | 163.11169 | C11H14O | Alkyl-phenylketones |
| Usaramine | 13.256 | 352.16691 | 17743 | 352.17001 | C18H25NO6 | Macrolides and analogues |
| Tyrosine | 1.096 | 182.08128 | 124206 | 182.08118 | C9H11NO3 | Tyrosine and derivatives |
| Tryptophan | 2.461 | 205.09721 | 1372163 | 205.09715 | C11H12N2O2 | Indolyl carboxylic acids and derivatives |
| Tris(2-butoxyethyl) phosphate | 24.105 | 399.25038 | 1010213 | 399.25061 | C18H39O7P | Trialkyl phosphates |
| TRIGONELLINE | 0.863 | 138.05499 | 194783 | 138.05499 | C7H7NO2 | Alkaloids and derivatives |
| Tricaproin (C6:0) | 17.516 | 409.25675 | 12867 | 409.25656 | C21H38O6 | Triacylglycerols |
| Trenbolone | 17.184 | 271.16986 | 33546 | 271.16931 | C18H22O2 | Estrogens and derivatives |
| trans-Zeatin-riboside | 5.598 | 352.16181 | 16636 | 352.16205 | C15H21N5O5 | Purine nucleosides |
| Trans-Zeatin-o-glucoside | 2.819 | 382.17032 | 15746 | 382.17212 | C16H23N5O6 | Fatty acyl glycosides of mono- and disaccharides |
| trans-Ferulic acid * | 13.773 | 177.05467 | 656310 | 177.05463 | C10H10O4 | Hydroxycinnamic acids |
| trans-Cinnamaldehyde | 7.463 | 133.06506 | 179199 | 133.06531 | C9H8O | Cinnamaldehydes |
| trans-Caffeic acid | 12.539 | 181.05031 | 17688 | 181.04953 | C9H8O4 | Hydroxycinnamic acids |
| Tramadol N-Oxide | 5.762 | 280.19049 | 14192 | 280.1907 | C16H25NO3 | Anisoles |
| Thioridazine | 43.383 | 371.16347 | 3727 | 371.16168 | C21H26N2S2 | Phenothiazines |
| Tetramethylscutellarein | 15.16 | 365.10138 | 7714 | 365.10001 | C19H18O6 | 7-O-methylated flavonoids |
| Tetrahydroalstonine | 5.504 | 375.15569 | 23377 | 375.16 | C21H24N2O3 | Yohimbine alkaloids |
| Tetracycline | 19.067 | 445.16543 | 11607 | 445.16049 | C22H24N2O8 | Tetracyclines |
| Tanshinone Iia * | 25.083 | 295.1324 | 5016436 | 295.13287 | C19H18O3 | Tanshinones, isotanshinones, and derivatives |
| Tangeritin | 18.364 | 373.12964 | 19270 | 373.13 | C20H20O7 | 8-O-methylated flavonoids |
| Syringic acid | 6.496 | 199.061 | 28376 | 199.06 | C9H10O5 | Gallic acid and derivatives |
| Syringaldehyde | 7.622 | 183.0658 | 14073 | 183.06519 | C9H10O4 | Methoxyphenols |
| synephrine | 1.819 | 168.10182 | 15828 | 168.10001 | C9H13NO2 | 1-hydroxy-2-unsubstituted benzenoids |
| SUCROSE | 43.37 | 365.10588 | 13563 | 365.10501 | C12H22O11 | O-glycosyl compounds |
| STACHYOSE | 0.775 | 689.21063 | 174932 | 689.211 | C24H42O21 | Oligosaccharides |
| Soyasapogenol B base + O-HexA-HexA-dHex; PlaSMA ID-3477 | 18.39 | 957.50774 | 12615 | 957.50348 | C48H76O19 | Triterpene saponins |
| skullcapflavone II | 17.225 | 375.10845 | 12562 | 375.10739 | C19H18O8 | 8-O-methylated flavonoids |
| skimmin | 5.258 | 325.09274 | 41136 | 325.0918 | C15H16O8 | Coumarin glycosides |
| skimmianine | 10.732 | 284.09302 | 8584 | 284.09 | C14H15NO4 | Dihydrofuranoquinolines |
| sinomenine | 6.414 | 352.15058 | 100184 | 352.14999 | C19H23NO4 | Morphinans |
| Sinapoylcholine | 4.319 | 310.16536 | 15915 | 310.16434 | C16H24NO5 | Coumaric acids and derivatives |
| Simazine | 1.277 | 202.08657 | 34075 | 202.0854 | C7H12ClN5 | Chloro-s-triazines |
| Seneciphylline | 13.853 | 334.15735 | 11290 | 334.16 | C18H23NO5 | Alkaloids and derivatives |
| Scopoletin | 6.537 | 193.05017 | 19875 | 193.05 | C10H8O4 | 7-hydroxycoumarins |
| Schisandrin C | 2.099 | 407.14568 | 26925 | 407.14999 | C22H24O6 | Hydrolyzable tannins |
| Schinifoline | 6.427 | 258.18127 | 11547 | 258.17999 | C17H23NO | Hydroquinolones |
| Salvianolic acid A * | 5.994 | 495.12924 | 26382 | 495.13 | C26H22O10 | Stilbenes |
| Salicylic acid | 3.948 | 139.03873 | 1546536 | 139.039 | C7H6O3 | Salicylic acids |
| Rosuvastatin | 2.153 | 482.17807 | 35399 | 482.1756 | C22H28FN3O6S | Phenylpyrimidines |
| ROSMARINIC ACID | 10.241 | 361.09274 | 71705 | 361.09201 | C20H11BrClF3N4O2 | Aromatic anilides |
| Retinoic acid | 23.55 | 301.2155 | 677363 | 301.21619 | C20H28O2 | Retinoids |
| Resacetophenone | 9.847 | 153.05522 | 13619 | 153.05499 | C8H8O3 | Alkyl-phenylketones |
| Rebemide | 4.821 | 178.12296 | 9033 | 178.12263 | C11H15NO | Benzamides |
| quinine anhydre | 24.054 | 347.16562 | 8365 | 347.17001 | C20H24N2O2 | Cinchona alkaloids |
| Pyrrolo[1,2-a]pyrazine-1,4-dione, hexahydro-3-[(4-hydroxyphenyl)methyl]- | 4.971 | 261.12309 | 19977 | 261.12299 | C14H16N2O3 | Alpha amino acids and derivatives |
| Pyrrolo[1,2-a]pyrazine-1,4-dione, hexahydro-3-(1-methylethyl)- | 5.244 | 197.12862 | 84122 | 197.12801 | C10H16N2O2 | Alpha amino acids and derivatives |
| Pyrocatechol | 3.948 | 111.04415 | 257353 | 111.0441 | C6H6O2 | Catechols |
| Pyrimethamine | 22.495 | 249.09115 | 13876 | 249.09019 | C12H13ClN4 | Aminopyrimidines and derivatives |
| Pyocyanin | 15.148 | 211.0872 | 102878 | 211.08659 | C13H10N2O | Phenazines and derivatives |
| Psoralen | 12.446 | 187.03939 | 79356 | 187.039 | C11H6O3 | Psoralens |
| PSICOSE | 0.8 | 203.0529 | 71755 | 203.05299 | C6H12O6 | Monosaccharides |
| Prunetin | 10.48 | 285.0767 | 16238 | 285.07599 | C16H12O5 | 7-O-methylisoflavones |
| Propazine-2-hydroxy | 6.198 | 212.15096 | 18350 | 212.1506 | C9H17N5O | N-aliphatic s-triazines |
| PROLINE | 0.889 | 116.07007 | 710193 | 116.071 | C5H9NO2 | Proline and derivatives |
| Progesterone | 21.05 | 315.23257 | 18994 | 315.2319 | C21H30O2 | Gluco/mineralocorticoids, progestogins and derivatives |
| POTASSIUM SORBATE | 3.723 | 113.05974 | 11795 | 113.06 | C6H7O2 | Medium-chain fatty acids |
| Podophyllotoxin | 20.963 | 437.12142 | 11077 | 437.12 | C22H22O8 | Podophyllotoxins |
| P-OCTOPAMINE | 9.697 | 136.07589 | 21840 | 136.075 | C8H11NO2 | 1-hydroxy-2-unsubstituted benzenoids |
| p-methoxycinnamic acid ethyl ester | 10.467 | 207.10086 | 4829495 | 207.10001 | C12H14O3 | Cinnamic acid esters |
| Plumericin | 9.116 | 291.08763 | 11138 | 291.0863 | C15H14O6 | Terpene lactones |
| Phytosphingosine | 15.314 | 318.30109 | 15444 | 318.29941 | C18H39NO3 | Lipids |
| Phorbol | 13.403 | 365.19453 | 10543 | 365.19 | C20H28O6 | Tigliane and ingenane diterpenoids |
| Phloretin | 10.241 | 275.09161 | 8745 | 275.0914 | C15H14O5 | 2'-Hydroxy-dihydrochalcones |
| Phenylalanylisoleucine (isomer of 1329); PlaSMA ID-1328 | 5.367 | 279.17082 | 23781 | 279.17181 | C15H22N2O3 | Dipeptides |
| PHENYLALANINE | 1.558 | 166.08587 | 2228651 | 166.08701 | C9H11NO2 | Phenylalanine and derivatives |
| Phenylacetaldehyde | 15.988 | 121.06528 | 10096 | 121.0639 | C8H8O | Phenylacetaldehydes |
| Peonidin-3-O-glucoside | 9.833 | 463.12333 | 369068 | 463.12292 | C22H23O11 | Anthocyanidin-3-O-glycosides |
| Pentose + Proline | 1.161 | 274.09234 | 449778 | 274.0932 | C11H15NO7 | Amino acids |
| Pelargonin | 6.469 | 595.16693 | 13834 | 595.16626 | C27H31O15 | Anthocyanidin-5-O-glycosides |
| p-Coumaraldehyde | 10.812 | 149.06004 | 11290 | 149.05971 | C9H8O2 | Cinnamaldehydes |
| PC(16:0/0:0) | 23.977 | 496.34026 | 46892 | 496.33981 | C24H50NO7P | 1-acyl-sn-glycero-3-phosphocholines |
| Pantothenate | 2.408 | 220.11807 | 93471 | 220.1179 | C9H17NO5 | Secondary alcohols |
| Palmatine | 9.561 | 352.15433 | 184035 | 352.15378 | C21H22NO4 | Alkaloids |
| Paeonol * | 6.619 | 167.0708 | 13141 | 167.07027 | C9H10O3 | Alkyl-phenylketones |
| Paclobutrazole | 17.463 | 294.13755 | 31355 | 294.13681 | C15H20ClN3O | Amphetamines and derivatives |
| Oxysophocarpine | 21.844 | 285.14908 | 13906 | 285.14999 | C15H22N2O2 | Matrine alkaloids |
| Oxypeucedanin hydrate | 13.365 | 305.10243 | 12772 | 305.10001 | C16H16O6 | Psoralens |
| Oxamniquine | 19.683 | 280.17038 | 45351 | 280.16556 | C14H21N3O3 | Nitroquinolines and derivatives |
| Ononin * | 10.905 | 431.13239 | 6377909 | 431.13 | C22H22O9 | Isoflavonoid O-glycosides |
| ONONETIN | 16.58 | 259.09748 | 18485 | 259.0965 | C15H14O4 | Stilbenes |
| Olopatadine | 16.66 | 338.17586 | 18003 | 338.17511 | C21H23NO3 | Dibenzoxepines |
| O-desmethylvenlafaxine | 8.151 | 264.19635 | 26893 | 264.1958 | C16H25NO2 | Cyclohexanols |
| Octopamine, N-feruloyl- | 12.742 | 352.1188 | 10237 | 352.12 | C18H19NO5 | Hydroxycinnamic acids and derivatives |
| Nylidrin | 11.691 | 300.19283 | 12770 | 300.1958 | C19H25NO2 | Phenylpropanes |
| Nuciferine | 21.532 | 296.1653 | 88897 | 296.16 | C19H21NO2 | Aporphines |
| Norvaline | 8.611 | 118.06535 | 14062 | 118.0642 | C5H11NO2 | L-alpha-amino acids |
| Norharman | 3.935 | 169.07627 | 104576 | 169.07602 | C11H8N2 | Beta carbolines |
| Nodakenitin | 10.967 | 247.09471 | 19984 | 247.09 | C14H14O4 | Psoralens |
| Nobiletin | 17.117 | 403.13958 | 37966 | 403.14001 | C21H22O8 | 8-O-methylated flavonoids |
| N-Methylcytisine | 16.554 | 227.10761 | 12020 | 227.11 | C12H16N2O | Cytisine and derivatives |
| N-isopropyl-2-(5-methoxy-4,8,8-trimethyl-2-oxo-2,8,9,10-tetrahydropyrano[2,3-f]chromen-3-yl)acetamide | 16.175 | 396.18146 | 74570 | 396.17999 | C21H27NO5 | Angular pyranocoumarins |
| N-isopentyl-2-(3,4,9-trimethyl-7-oxo-7H-furo[2,3-f]chromen-8-yl)acetamide | 18.454 | 378.17082 | 14551 | 378.17001 | C21H25NO4 | Angular furanocoumarins |
| NICOTINATE | 1.096 | 124.03926 | 154802 | 124.04 | C6H5NO2 | Pyridinecarboxylic acids |
| Nicotinamide | 1.006 | 123.05504 | 70735 | 123.0553 | C6H6N2O | Nicotinamides |
| N-Fructosyl phenylalanine | 1.807 | 328.13882 | 3380687 | 328.13879 | C15H21NO7 | N-Fructosyl amino acids |
| N-Fructosyl isoleucine | 1.187 | 276.14386 | 1983642 | 276.1445 | C12H23NO7 | N-Fructosyl amino acids |
| NCGC00386106-01 | 22.052 | 261.14926 | 21350 | 261.14801 | C16H22O4 | Macrolides and analogues |
| NCGC00386065-01 | 14.749 | 343.11836 | 52239 | 343.117 | C19H20O7 | Benzofurans |
| NCGC00386020-01 | 14.232 | 293.21144 | 180662 | 293.211 | C18H28O3 | Prostaglandins and related compounds |
| NCGC00385948-01 | 4.374 | 288.12411 | 15442 | 288.12299 | C16H17NO4 | Alkaloids and derivatives |
| NCGC00385911-01 | 24.041 | 219.17449 | 79900 | 219.174 | C15H24O2 | Sesquiterpenoids |
| NCGC00385879-01 | 5.012 | 313.07219 | 14312 | 313.07001 | C17H12O6 | Naphthalenecarboxylic acids |
| NCGC00385841-01 | 15.645 | 249.14944 | 20309 | 249.149 | C15H20O3 | Xanthanolides |
| NCGC00385811-01 | 19.834 | 415.21162 | 319298 | 415.211 | C24H32O7 | Dibenzylbutane lignans |
| NCGC00385674-01!6,8-dihydroxy-3-(10-hydroxyundecyl)-3,4-dihydroisochromen-1-one [IIN-based on: CCMSLIB00000846799] | 23.222 | 333.20641 | 23566 | 333.20599 | C20H30O5 | Hydroxybenzoic acid derivatives |
| NCGC00385557-01_C48H76O18_ | 19.383 | 941.5097 | 146015 | 941.51001 | C48H76O18 | Triterpene saponins |
| NCGC00385492-01 | 21.962 | 215.10712 | 126557 | 215.106 | C14H16O3 | Isobenzofurans |
| NCGC00385441-01 | 10.583 | 369.15662 | 34571 | 369.15201 | C16H26O8 | Terpene glycosides |
| NCGC00385425-01 | 26.913 | 487.21212 | 44178 | 487.215 | C21H36O11 | O-glycosyl compounds |
| NCGC00385412-01 | 26.848 | 467.18379 | 8720 | 467.189 | C21H32O10 | Abscisic acids and derivatives |
| NCGC00385365-01 | 9.253 | 197.11756 | 40649 | 197.117 | C11H16O3 | Benzofurans |
| NCGC00385364-01 | 14.876 | 301.07124 | 37954 | 301.07001 | C16H12O6 | Isoflavones |
| NCGC00385309-01 | 16.554 | 245.11729 | 238745 | 245.117 | C15H16O3 | Butenolides |
| NCGC00385243-01 | 7.371 | 227.12906 | 23848 | 227.12801 | C12H18O4 | Jasmonic acids |
| NCGC00385236-01 | 11.77 | 553.13651 | 23787 | 553.13397 | C28H24O12 | 2-arylbenzofuran flavonoids |
| NCGC00385235-01 | 19.22 | 333.20617 | 76419 | 333.20499 | C20H30O5 | Gamma butyrolactones |
| NCGC00385123-01 | 19.834 | 432.23868 | 57764 | 432.23801 | C22H30O6 | Tigliane and ingenane diterpenoids |
| NCGC00385078-01 | 18.57 | 295.13316 | 24761 | 295.133 | C19H18O3 | 2-arylbenzofuran flavonoids |
| NCGC00385025-01 | 10.812 | 556.14628 | 31331 | 556.14502 | C27H22O12 | 2-arylbenzofuran flavonoids |
| NCGC00385013-01 | 10.557 | 271.0972 | 19620 | 271.09601 | C16H14O4 | 2-benzopyrans |
| NCGC00385003-01 | 23.825 | 425.29024 | 53981 | 425.29001 | C24H42O7 | Colensane and clerodane diterpenoids |
| NCGC00384971-01 | 6.66 | 469.13302 | 10860 | 469.13199 | C19H26O12 | O-glycosyl compounds |
| NCGC00384951-01 | 19.565 | 195.13804 | 229246 | 195.13699 | C12H20O3 | Butenolides |
| NCGC00384860-01 | 9.833 | 287.09186 | 64935 | 287.091 | C16H14O5 | Pterocarpans |
| NCGC00384813-01 | 8.274 | 359.07766 | 16569 | 359.07599 | C18H14O8 | Phenylbenzo-1,4-dioxanes |
| NCGC00384799-01 | 17.954 | 315.15894 | 1060123 | 315.159 | C19H22O4 | Colensane and clerodane diterpenoids |
| NCGC00384618-01 | 10.719 | 629.33338 | 164080 | 629.33002 | C33H50O10 | Steroidal saponins |
| NCGC00384596-01 | 8.87 | 556.14626 | 47879 | 556.14502 | C27H22O12 | 2-arylbenzofuran flavonoids |
| NCGC00384574-01 | 15.658 | 349.16548 | 63338 | 349.16501 | C19H24O6 | Steroid lactones |
| NCGC00384531-01 | 24.679 | 431.18524 | 105923 | 431.18301 | C25H28O5 | 8-prenylated isoflavanones |
| NCGC00384481-01 | 18.125 | 313.1443 | 38606 | 313.14301 | C19H22O5 | Terpene lactones |
| NCGC00381434-01 | 10.518 | 327.15968 | 95614 | 327.159 | C20H22O4 | 2-arylbenzofuran flavonoids |
| NCGC00381359-01 | 8.546 | 245.12944 | 30713 | 245.12801 | C14H18N2O3 | Dipeptides |
| NCGC00381179-01 | 23.902 | 265.08705 | 13130 | 265.08499 | C17H14O4 | 1-hydroxy-2-unsubstituted benzenoids |
| NCGC00381022-01 | 5.381 | 265.14387 | 91692 | 265.14301 | C15H22O5 | Terpene lactones |
| NCGC00380877-01 | 8.001 | 369.11958 | 13771 | 369.11801 | C17H20O9 | Quinic acids and derivatives |
| NCGC00380809-01 | 5.176 | 342.17039 | 22485 | 342.17001 | C20H23NO4 | Aporphines |
| NCGC00380795-01 | 19.488 | 313.14352 | 1085127 | 313.14301 | C19H22O5 | C19-gibberellin 6-carboxylic acids |
| NCGC00380749-01 | 13.365 | 567.18704 | 23459 | 567.18597 | C30H32O12 | Terpene glycosides |
| NCGC00380626-01 | 9.279 | 209.11709 | 779702 | 209.117 | C12H18O4 | Cyclohexenones |
| NCGC00380585-01 | 13.64 | 463.13903 | 15402 | 463.13699 | C24H24O8 | Depsides and depsidones |
| NCGC00380572-01 | 12.271 | 419.17123 | 12971 | 419.17001 | C22H26O8 | Furanoid lignans |
| NCGC00380524-01 | 11.423 | 736.18931 | 72915 | 736.18701 | C36H30O16 | 2-arylbenzofuran flavonoids |
| NCGC00380522-01 | 16.87 | 317.21192 | 38566 | 317.211 | C20H30O4 | Diterpenoids |
| NCGC00380393-01 | 11.563 | 247.13352 | 26327 | 247.132 | C15H20O4 | Terpene lactones |
| NCGC00380364-01 | 16.449 | 767.45912 | 32241 | 767.45697 | C41H66O13 | Triterpenoids |
| NCGC00380339-01 | 2.34 | 260.12843 | 18123 | 260.12799 | C15H19NO4 | Terpene lactones |
| NCGC00380239-01 | 16.279 | 437.34171 | 300456 | 437.34201 | C30H50O5 | Triterpenoids |
| NCGC00380212-01 | 8.233 | 480.24528 | 10338 | 480.24399 | C21H34O11 | O-glycosyl compounds |
| NCGC00380167-01 | 15.225 | 353.13738 | 34744 | 353.13599 | C19H22O5 | Methoxyphenols |
| NCGC00347704-02 | 20.283 | 415.21175 | 16949 | 415.211 | C24H32O7 | Naphthofurans |
| NCGC00347665-02 | 2.448 | 627.24182 | 16027 | 627.23938 | C31H40O12 | Limonoids |
| NCGC00347634-02 | 18.044 | 319.19126 | 24755 | 319.19 | C19H26O4 | Androgens and derivatives |
| NCGC00347585-02 | 22.25 | 421.19906 | 33472 | 421.19901 | C21H34O6 | Naphthofurans |
| NCGC00347419-02 | 14.6 | 283.09653 | 206831 | 283.09601 | C17H16O5 | Dimethoxybenzenes |
| NCGC00347365-02 | 19.501 | 335.1262 | 18716 | 335.12601 | C19H20O4 | Linear pyranocoumarins |
| NCGC00347357-02 | 23.785 | 471.34749 | 31802 | 471.34601 | C30H48O5 | Triterpenoids |
| NCGC00180746-02 | 16.896 | 487.34286 | 35865 | 487.341 | C30H48O6 | Triterpenoids |
| NCGC00180744-03 | 11.112 | 331.15444 | 142005 | 331.15302 | C19H24O6 | Eremophilane, 8,9-secoeremophilane and furoeremophilane sesquiterpenoids |
| NCGC00180657-03 | 23.965 | 309.11266 | 20338 | 309.112 | C19H20O6 | Tetralins |
| NCGC00180593-02 | 10.253 | 738.20403 | 11649 | 738.203 | C36H32O16 | Cyclobutane lignans |
| NCGC00180529-03 | 26.693 | 657.44439 | 5989 | 657.44299 | C33H57N3O9 | Cyclic depsipeptides |
| NCGC00180527-03 | 7.463 | 463.16101 | 228641 | 463.159 | C23H28O11 | Terpene glycosides |
| NCGC00180342-02 | 14.016 | 354.13442 | 26252 | 354.134 | C20H19NO5 | Indanones |
| NCGC00180293-02 | 16.357 | 251.16484 | 25050 | 251.164 | C15H22O3 | Eudesmane, isoeudesmane or cycloeudesmane sesquiterpenoids |
| NCGC00180259-02 | 7.689 | 503.19106 | 38258 | 503.19 | C26H32O11 | 2-arylbenzofuran flavonoids |
| NCGC00179861-02 | 13.456 | 221.1903 | 8503 | 221.189 | C15H26O2 | Sesquiterpenoids |
| NCGC00179768-02 | 16.58 | 343.11866 | 38502 | 343.117 | C19H20O7 | Tetralins |
| NCGC00179704-04 | 21.66 | 235.16969 | 37461 | 235.16901 | C15H24O3 | Eudesmane, isoeudesmane or cycloeudesmane sesquiterpenoids |
| NCGC00169984-03 | 10.467 | 499.12432 | 26033 | 499.12299 | C25H24O12 | Quinic acids and derivatives |
| NCGC00169692-02 | 23.851 | 293.1172 | 266363 | 293.117 | C19H18O4 | Phenanthrols |
| NCGC00169670-02 | 9.819 | 598.25123 | 19683 | 598.25 | C28H36O13 | Lignan glycosides |
| NCGC00169621-02 | 15.021 | 247.09642 | 251913 | 247.09599 | C14H14O4 | Kavalactones |
| NCGC00169296-02 | 13.052 | 461.10917 | 10362 | 461.108 | C22H20O11 | Flavonoid-7-O-glucuronides |
| NCGC00169117-02 | 20.038 | 405.17034 | 59758 | 405.16901 | C25H26O6 | Pyranochromenes |
| NCGC00169103-03 | 14.138 | 325.10775 | 140692 | 325.10699 | C19H18O6 | Tetralins |
| NCGC00169049-03 | 10.467 | 225.11266 | 158254 | 225.112 | C12H16O4 | Isobenzofurans |
| NCGC00169029-02 | 15.988 | 251.16483 | 13735 | 251.164 | C15H22O3 | Terpene lactones |
| NCGC00168812-02 | 21.456 | 439.35753 | 70498 | 439.35699 | C30H48O3 | Triterpenoids |
| NCGC00091907-03 | 16.357 | 233.15399 | 45732 | 233.153 | C15H22O3 | Sesquiterpenoids |
| N-Carbobenzyloxyleucine | 3.004 | 266.139 | 21286 | 266.13849 | C14H19NO4 | Leucine and derivatives |
| N-Benzyloxycarbonylglycine | 3.75 | 210.0763 | 19768 | 210.076 | C10H11NO4 | Benzyloxycarbonyls |
| naringenin | 13.12 | 273.07644 | 33296 | 273.07571 | C15H12O5 | Flavanones |
| Naphthalene | 14.775 | 128.06207 | 18652 | 128.0621 | C10H8 | Naphthalenes |
| Nandrolone | 14.246 | 275.20071 | 214311 | 275.20123 | C18H26O2 | Estrogens and derivatives |
| N-ACETYLPROLINE | 2.551 | 158.08158 | 92284 | 158.08099 | C7H11NO3 | Proline and derivatives |
| N-ACETYL-L-PHENYLALANINE | 8.124 | 208.09751 | 18938 | 208.097 | C11H13NO3 | Phenylalanine and derivatives |
| N-ACETYL-D-TRYPTOPHAN | 4.184 | 247.10744 | 62503 | 247.108 | C13H14N2O3 | N-acyl-alpha amino acids |
| N6-Threonylcarbamoyladenosine | 5.367 | 413.14217 | 28742 | 413.14151 | C15H20N6O8 | Purine nucleosides |
| N6-Isopentenyladenosine | 8.625 | 336.1673 | 26782 | 336.1676 | C15H21N5O4 | Purine nucleosides |
| N2,N2-Dimethylguanosine | 2.286 | 312.13105 | 55511 | 312.1297 | C12H17N5O5 | Purine nucleosides |
| N,N-Dimethyldodecylamine N-oxide | 15.135 | 230.24747 | 8910 | 230.2478 | C14H31NO | Long-chain alkyl amine oxides |
| N-(2,4-dimethylphenyl)formamide | 10.597 | 150.09165 | 30016 | 150.09129 | C9H11NO | Anilides |
| N-(2-(2-(2-hydroxy-5-oxo-4,5-dihydro-3H-benzo[e][1,4]diazepin-3-yl)acetamido)ethyl)-1-methyl-1H-indole-2-carboxamide | 5.966 | 434.18191 | 30596 | 434.17999 | C23H23N5O4 | Indolecarboxamides and derivatives |
| N-(1-hydroxy-3-methylbutan-2-yl)-2-((4-oxo-3-phenyl-4H-chromen-7-yl)oxy)acetamide | 1.109 | 382.16159 | 54719 | 382.16 | C22H23NO5 | Isoflavones |
| myrcene | 15.352 | 159.11694 | 11276 | 159.12 | C10H16 | Acyclic monoterpenoids |
| Morphine | 17.821 | 308.12933 | 7807 | 308.1257 | C17H19NO3 | Morphinans |
| monolinolein | 28.147 | 355.2846 | 28108 | 355.28427 | C21H38O4 | Lineolic acids and derivatives |
| MMV688888 | 18.071 | 350.17761 | 6797 | 350.17728 | C20H20FN5 | Pyridinylpyrimidines |
| MMV688469 | 2.192 | 276.12367 | 17680 | 276.12418 | C16H13N5 | Naphthalenes |
| Miltirone | 26.501 | 305.15132 | 56383 | 305.14999 | C19H22O2 | Tanshinones, isotanshinones, and derivatives |
| Metolachlor morpholinone | 4.48 | 234.14917 | 11521 | 234.1489 | C14H19NO2 | Phenylmorpholines |
| Methylnissolin-3-O-glucoside | 11.614 | 463.16066 | 277527 | 463.16 | C23H26O10 | Pterocarpans |
| Methyl trans-styryl ketone | 11.302 | 147.08085 | 18600 | 147.0804 | C10H10O | Styrenes |
| Methyl trans-cinnamic acid | 26.463 | 163.07566 | 12162 | 163.07539 | C10H10O2 | Cinnamic acids |
| Methyl nicotinic acid | 5.162 | 138.0553 | 27295 | 138.05496 | C7H7NO2 | Pyridinecarboxylic acids |
| methyl 1-oxo-2-(o-tolyl)-1,2,3,6,7,7a-hexahydro-3a,6-epoxyisoindole-7-carboxylate | 7.265 | 322.1091 | 8288 | 322.10501 | C17H17NO4 | Phenylpyrrolidines |
| methyl (3R,4R,5R)-5-[(2R,3R,4S,5S,6R)-4,5-dihydroxy-6-(hydroxymethyl)-3-[(E)-3-phenylprop-2-enoyl]oxyoxan-2-yl]oxy-3,4-dihydroxycyclohexene-1-carboxylate | 8.546 | 503.15347 | 14240 | 503.1521 | C23H28O11 | Fatty acyl glycosides of mono- and disaccharides |
| Methoxyindoleacetic acid | 6.414 | 206.08189 | 14396 | 206.08118 | C11H11NO3 | Indole-3-acetic acid derivatives |
| Meranzin hydrate | 8.546 | 301.10798 | 161895 | 301.10999 | C15H18O5 | Coumarins and derivatives |
| Mellein | 6.158 | 179.07051 | 64813 | 179.0697 | C10H10O3 | 2-benzopyrans |
| Melezitose | 0.775 | 527.15848 | 87058 | 527.15826 | C18H32O16 | Oligosaccharides |
| Maltol | 3.031 | 127.03893 | 344698 | 127.0387 | C6H6O3 | Pyranones and derivatives |
| Malonyltryptophan | 8.625 | 291.09814 | 17431 | 291.0972 | C14H14N2O5 | N-acyl-alpha amino acids |
| magnolol | 19.079 | 267.1387 | 84523 | 267.14001 | C18H18O2 | Biphenyls and derivatives |
| Lycorine-diacetate | 3.559 | 372.14502 | 28086 | 372.14417 | C20H21NO6 | Lycorine-type amaryllidaceae alkaloids |
| lycorine | 6.13 | 310.10801 | 16592 | 310.10999 | C16H17NO4 | Lycorine-type amaryllidaceae alkaloids |
| Lup-20(29)-en-28-oic acid, 6-hydroxy-3-oxo-, (5xi,9xi,18xi)- | 23.785 | 453.33688 | 36461 | 453.336 | C30H46O4 | Triterpenoids |
| L-Tryptophan | 4.876 | 205.09716 | 17289 | 205.09715 | C11H12N2O2 | Indolyl carboxylic acids and derivatives |
| L-Proline | 2.991 | 116.07059 | 54563 | 116.0706 | C5H9NO2 | Proline and derivatives |
| LPC 18:3 | 21.596 | 518.32422 | 224856 | 518.32233 | C26H48NO7P | Lipids |
| Linoleic acid | 22.078 | 281.24771 | 34634 | 281.2475 | C18H32O2 | Lineolic acids and derivatives |
| linderalactone | 16.554 | 267.09725 | 11836 | 267.10001 | C15H16O3 | Butenolides |
| Limonene | 12.15 | 137.13257 | 22220 | 137.131 | C10H16 | Menthane monoterpenoids |
| Licoflavone A | 16.845 | 323.12685 | 72490 | 323.13 | C20H18O4 | 6-prenylated flavones |
| Licochalcone A | 21.962 | 339.15771 | 13218 | 339.16 | C21H22O4 | Retrochalcones |
| Licarin A | 10.346 | 327.15677 | 12770 | 327.16 | C20H22O4 | 2-arylbenzofuran flavonoids |
| Leucylphenylalanine | 5.749 | 279.17052 | 57328 | 279.17249 | C15H22N2O3 | Dipeptides |
| Leucylleucyltyrosine | 6.578 | 408.24948 | 10384 | 408.24982 | C21H33N3O5 | Peptides |
| leonurine | 16.397 | 312.15954 | 10571 | 312.16 | C14H21N3O5 | Gallic acid and derivatives |
| L-Cysteine | 5.067 | 122.02357 | 113271 | 122.027 | C3H7NO2S | Cysteine and derivatives |
| Lauryldiethanolamine | 15.073 | 274.27426 | 24552 | 274.27411 | C16H35NO2 | 1,2-aminoalcohols |
| Laurocapram | 29.28 | 282.27879 | 164563 | 282.27911 | C18H35NO | Caprolactams |
| Lauramidopropyl betaine | 17.383 | 343.29597 | 11199 | 343.2955 | C19H39N2O3 | Alpha amino acids |
| L-Asarinin | 8.98 | 355.11864 | 27373 | 355.12 | C20H18O6 | Furanoid lignans |
| L-Arginine | 2.246 | 175.12302 | 25635 | 175.11948 | C6H14N4O2 | L-alpha-amino acids |
| lapachol | 15.604 | 243.10242 | 9075 | 243.10001 | C15H14O3 | Vitamin K compounds |
| L-(-)-Phenylalanine | 1.384 | 166.08611 | 37180 | 166.08678 | C9H11NO2 | Phenylalanine and derivatives |
| Koparin | 7.397 | 301.07143 | 101747 | 301.07065 | C16H12O6 | 3'-hydroxy,4'-methoxyisoflavonoids |
| Kojic Acid | 3.072 | 143.03377 | 419055 | 143.03329 | C6H6O4 | Pyranones and derivatives |
| Khasianine | 7.622 | 744.42621 | 5613 | 744.42999 | C39H63NO11 | Steroidal saponins |
| Kaempferol | 13.921 | 287.05543 | 14826 | 287.05499 | C15H10O6 | Flavonols |
| Isoxsuprine | 9.656 | 302.1752 | 21719 | 302.17572 | C18H23NO3 | Phenylpropanes |
| Isotetrandrine | 13.84 | 645.29248 | 8114 | 645.28998 | C38H42N2O6 | Lignans, neolignans and related compounds |
| Isorhamnetin | 10.119 | 339.05086 | 6221 | 339.04999 | C16H12O7 | Flavonols |
| Isopropylidenylacetyl-marmesin | 13.813 | 329.13914 | 50014 | 329.14001 | C19H20O5 | Psoralens |
| Isophorone | 10.279 | 139.11187 | 41866 | 139.11169 | C9H14O | Cyclohexenones |
| Isoleucylserine | 1.225 | 219.13434 | 22327 | 219.1356 | C9H18N2O4 | Dipeptides |
| Isoleucylaspartate | 3.342 | 247.1295 | 12508 | 247.1346 | C10H18N2O5 | Dipeptides |
| ISOLEUCINE | 1.109 | 132.10188 | 501179 | 132.10201 | C6H13NO2 | Isoleucine and derivatives |
| Isoimperatorin | 19.342 | 293.08142 | 14332 | 293.07999 | C16H14O4 | Psoralens |
| Isofraxidin | 8.444 | 223.06089 | 109539 | 223.0601 | C11H10O5 | 7-hydroxycoumarins |
| Isoflavone base + 1O, 2MeO, O-Hex | 11.098 | 461.145 | 72920 | 461.1456 | C23H24O10 | Isoflavonoid O-glycosides |
| Isocorydine | 4.535 | 364.15072 | 20395 | 364.14999 | C20H23NO4 | Aporphines |
| Isobavachin | 16.201 | 325.142 | 74377 | 325.14001 | C20H20O4 | 8-prenylated flavanones |
| Isobavachalcone | 2.819 | 347.12484 | 18569 | 347.12601 | C20H20O4 | 3-prenylated chalcones |
| Isoastragaloside II | 19.328 | 849.46432 | 9013 | 849.46002 | C43H70O15 | Cucurbitacin glycosides |
| Irinotecan Hydrochloride | 4.821 | 623.25723 | 28687 | 623.26001 | C33H39ClN4O6 | Camptothecins |
| Ingenol | 15.949 | 231.10232 | 15816 | 231.10001 | C20H28O5 | Tigliane and ingenane diterpenoids |
| Indolelactic acid | 3.505 | 206.08148 | 43872 | 206.0813 | C11H11NO3 | Indolyl carboxylic acids and derivatives |
| Indole-3-acetyl-L-valine | 7.078 | 275.13992 | 14075 | 275.13901 | C15H18N2O3 | Valine and derivatives |
| INDOLE-3-ACETATE | 7.662 | 176.07114 | 13505 | 176.071 | C10H9NO2 | Indole-3-acetic acid derivatives |
| Indirubin | 11.986 | 263.08275 | 26644 | 263.082 | C16H10N2O2 | Hydroxyindoles |
| Imazamox | 29.548 | 306.14903 | 14141 | 306.14481 | C15H19N3O4 | Alpha amino acids and derivatives |
| Ibuprofen | 6.96 | 207.13889 | 13643 | 207.138 | C13H18O2 | Phenylpropanoic acids |
| hydroquinidine | 6.359 | 349.18714 | 14105 | 349.19 | C20H26N2O2 | Cinchona alkaloids |
| Hydrocotarnine | 5.012 | 222.11345 | 13434 | 222.11247 | C12H15NO3 | Tetrahydroisoquinolines |
| H-Pro-Hyp-OH | 2.3 | 229.1184 | 62786 | 229.1189 | C10H16N2O4 | Dipeptides |
| Hosenkoside C | 17.423 | 1001.53381 | 6862 | 1001.53003 | C48H82O20 | Triterpenoids |
| honokiol | 18.247 | 267.13885 | 21967 | 267.14001 | C18H18O2 | Biphenyls and derivatives |
| Homogentisic acid | 5.844 | 169.04994 | 72753 | 169.0495 | C8H8O4 | 2(hydroxyphenyl)acetic acids |
| HOMOGENTISATE | 2.819 | 169.05043 | 41691 | 169.05 | C8H8O4 | 2(hydroxyphenyl)acetic acids |
| Harmane | 5.176 | 183.09332 | 31613 | 183.09167 | C12H10N2 | Harmala alkaloids |
| h_92_oxabolone | 17.012 | 291.19514 | 460717 | 291.19501 | C18H26O3 | Estrogens and derivatives |
| h_65_epitrenbolone | 19.746 | 253.15908 | 393149 | 253.158 | C18H22O2 | Estrogens and derivatives |
| h_315_oxymetholone_m2 | 21.175 | 299.23822 | 45224 | 299.23599 | C21H32O2 | Oxosteroids |
| h_268_isomere_de_oxymetholone_m2 | 21.175 | 317.24852 | 21920 | 317.24701 | C21H32O2 | Oxosteroids |
| h_14_19_norandrosterone | 18.953 | 259.20623 | 70638 | 259.20499 | C18H28O2 | Estrogens and derivatives |
| Guggulsterone E&Z | 24.957 | 335.20155 | 16636 | 335.20001 | C21H28O2 | Androgens and derivatives |
| Guanosine cyclic monophosphate | 2.031 | 346.05553 | 68206 | 346.05469 | C10H12N5O7P | 3',5'-cyclic purine nucleotides |
| GUANOSINE | 1.122 | 284.09973 | 141825 | 284.099 | C10H13N5O5 | Purine nucleosides |
| Guanine | 1.122 | 152.05681 | 282379 | 152.05701 | C5H5N5O | Purines and purine derivatives |
| Guaiacin | 20.58 | 329.17597 | 17698 | 329.17999 | C20H24O4 | Aryltetralin lignans |
| Griseofulvin | 15.697 | 353.07971 | 6376 | 353.07809 | C17H17ClO6 | Benzofurans |
| Gly-Leu | 1.303 | 189.12372 | 8881 | 189.1227 | C8H16N2O3 | Peptides |
| glycycoumarin | 13.747 | 369.13407 | 11390 | 369.13 | C21H20O6 | Hydroxyisoflavonoids |
| glycitin | 7.078 | 447.13022 | 12776 | 447.129 | C22H22O10 | Isoflavonoid O-glycosides |
| Glutamylphenylalanine | 4.835 | 295.12925 | 19128 | 295.12949 | C14H18N2O5 | Dipeptides |
| GLUCOSAMINE | 0.631 | 162.07614 | 215220 | 162.076 | C6H13NO5 | Hexoses |
| Gentiannine | 2.435 | 176.0711 | 17637 | 176.07001 | C10H9NO2 | Pyranopyridines |
| Genipin 1-gentiobioside | 7.383 | 573.1836 | 17150 | 573.17999 | C23H34O15 | Iridoid O-glycosides |
| gelsemine | 6.973 | 345.14593 | 17231 | 345.14999 | C20H22N2O2 | Gelsemium alkaloids |
| Gastrodin | 8.788 | 309.08763 | 11572 | 309.09 | C13H18O7 | Phenolic glycosides |
| gamma-sitosterol | 23.351 | 437.37398 | 19053 | 437.37537 | C29H50O | Stigmastanes and derivatives |
| gamma-Glutamylleucine | 3.935 | 261.14552 | 24659 | 261.14471 | C11H20N2O5 | Dipeptides |
| Gallic Acid Ethyl Ester | 9.629 | 221.0444 | 579284 | 221.03999 | C9H10O5 | Galloyl esters |
| Gallic acid | 1.451 | 171.02873 | 236401 | 171.02879 | C7H6O5 | Gallic acids |
| Gabexate | 30.078 | 322.1802 | 44671 | 322.1756 | C16H23N3O4 | Benzoic acid esters |
| Furanodienon | 13.442 | 253.12292 | 11781 | 253.12 | C15H18O2 | Germacrane sesquiterpenoids |
| FruLeuIle | 5.367 | 407.23914 | 140751 | 407.23901 | C18H34N2O8 | Peptides |
| Fraxetin | 6.81 | 209.04517 | 15972 | 209.03999 | C10H8O5 | 7,8-dihydroxycoumarins |
| Formononetin * | 15.098 | 269.08051 | 6307453 | 269.08081 | C16H12O4 | 4'-O-methylisoflavones |
| Flavonol base + 2MeO | 15.51 | 299.09165 | 136105 | 299.0928 | C17H14O5 | Flavonol O-glycosides |
| Feruloyl putrescine | 2.578 | 265.15513 | 22123 | 265.15469 | C14H20N2O3 | Hydroxycinnamic acids and derivatives |
| Ferulate * | 8.392 | 195.06576 | 118669 | 195.06599 | C10H10O4 | Hydroxycinnamic acids |
| falcarindiol | 22.949 | 225.16432 | 10455 | 225.164 | C17H24O2 | Long-chain fatty alcohols |
| Fabesetron | 20.168 | 294.16061 | 71281 | 294.1601 | C18H19N3O | 3-methylindoles |
| FA 18:4+1O | 15.842 | 293.21152 | 50923 | 293.21121 | C18H28O3 | Prostaglandins and related compounds |
| FA 18:3+1O | 24.771 | 295.22667 | 281108 | 295.228 | C18H30O3 | Lineolic acids and derivatives |
| Exemestane | 20.27 | 297.18617 | 28752 | 297.18491 | C20H24O2 | Androgens and derivatives |
| Evodine | 4.426 | 330.12848 | 15540 | 330.13 | C18H19NO5 | Furanoquinolines |
| Eupalinolide K | 1.225 | 385.16035 | 19300 | 385.16 | C20H26O6 | Germacranolides and derivatives |
| Eugenol | 7.635 | 165.09147 | 14448 | 165.091 | C10H12O2 | Methoxyphenols |
| Eudesmin | 5.504 | 387.17599 | 26270 | 387.17999 | C22H26O6 | Furanoid lignans |
| ethyl 3-(2,4-dihydroxy-6-methylpyridin-3-yl)-3-(3-hydroxyphenyl)propanoate | 1.343 | 318.13367 | 46013 | 318.13 | C17H19NO5 | Pyridinones |
| Erucamide | 34.355 | 338.3413 | 1448295 | 338.34161 | C22H43NO | Fatty amides |
| Eriosematin | 22.013 | 313.14416 | 48846 | 313.14001 | C19H20O4 | Pyranochromenes |
| Eremantholide A | 5.749 | 349.15044 | 60816 | 349.151 | C19H24O6 | Furofurans |
| Enterodiol | 19.079 | 303.15992 | 182501 | 303.15909 | C18H22O4 | Dibenzylbutanediol lignans |
| Ellagic acid | 8.558 | 303.01385 | 13032 | 303.01001 | C14H6O8 | Hydrolyzable tannins |
| ELAIDIC ACID | 25.987 | 283.26345 | 84486 | 283.26401 | C18H34O2 | Long-chain fatty acids |
| Diosmetin | 11.974 | 301.072 | 19040 | 301.07001 | C16H12O6 | 4'-O-methylated flavonoids |
| Dioscin | 19.631 | 891.47175 | 25913 | 891.46997 | C45H72O16 | Steroidal saponins |
| Dioctyl Phthalate | 34.2 | 391.28432 | 55553 | 391.284 | C24H38O4 | Benzoic acid esters |
| Dihydrotanshinone I * | 20.963 | 279.10148 | 6114885 | 279.10156 | C18H14O3 | Tanshinones, isotanshinones, and derivatives |
| Dihydropalmatine | 8.625 | 354.17062 | 27157 | 354.17001 | C21H23NO4 | Protoberberine alkaloids and derivatives |
| Dihydromuronic acid | 17.516 | 369.26365 | 126160 | 369.26401 | C21H36O5 | Gamma butyrolactones |
| Dihydroberberine | 8.392 | 338.13977 | 52085 | 338.14001 | C20H19NO4 | Protoberberine alkaloids and derivatives |
| Diethyl-phthalate | 14.07 | 223.0969 | 44827 | 223.0965 | C12H14O4 | Benzoic acid esters |
| Dibenz[a,j]acridine | 25.07 | 280.11112 | 7948 | 280.11209 | C21H13N | Benzacridines |
| Diacetylmorphine | 6.714 | 370.16607 | 23168 | 370.16489 | C21H23NO5 | Morphinans |
| Dextromethorphan | 7.933 | 272.19687 | 119419 | 272.2009 | C18H25NO | Morphinans |
| desmotroposantonin | 1.832 | 247.12878 | 17783 | 247.13 | C15H18O3 | Naphthofurans |
| DESMOSTEROL | 30.602 | 385.34688 | 12951 | 385.34698 | C27H44O | Cholesterols and derivatives |
| Dehydroevodiamine | 6.701 | 324.10894 | 11976 | 324.10999 | C19H15N3O | Quinazolines |
| dehydrocostus lactone | 11.885 | 231.1396 | 7881 | 231.138 | C15H18O2 | Guaianolides and derivatives |
| Dehydrocorydaline | 18.518 | 366.17105 | 10439 | 366.16943 | C22H24NO4 | Protoberberine alkaloids and derivatives |
| DEET | 2.192 | 192.1387 | 14152 | 192.13831 | C12H17NO | N,N-dialkyl-m-toluamides |
| Daphnetin | 2.273 | 179.03419 | 137366 | 179.03391 | C9H6O4 | 7,8-dihydroxycoumarins |
| daniellic acid | 24.413 | 317.20986 | 26936 | 317.20999 | C20H28O3 | Colensane and clerodane diterpenoids |
| Daidzin | 7.595 | 417.11874 | 25202 | 417.117 | C21H20O9 | Isoflavonoid O-glycosides |
| Daidzein | 11.329 | 255.0656 | 41820 | 255.0652 | C15H10O4 | Isoflavones |
| D-(+)-Glucosamine | 2.286 | 180.0885 | 82156 | 180.08717 | C6H13NO5 | Hexoses |
| Cystathionine | 11.872 | 223.07597 | 48848 | 223.07523 | C7H14N2O4S | L-cysteine-S-conjugates |
| Cyprodinil-TP CGA 249287 | 1.819 | 150.10299 | 12085 | 150.1026 | C8H11N3 | Aminopyrimidines and derivatives |
| Cyclo(Leu-Pro) | 7.676 | 211.14421 | 197541 | 211.14391 | C11H18N2O2 | Alpha amino acids and derivatives |
| Cyclic AMP | 1.317 | 330.06002 | 110502 | 330.06 | C10H12N5O6P | 3',5'-cyclic purine nucleotides |
| Curcumenol | 23.6 | 235.16941 | 16235 | 235.16925 | C15H22O2 | Guaianes |
| Cryptotanshinone * | 22.432 | 297.14781 | 12108840 | 297.1485 | C19H20O3 | Tanshinones, isotanshinones, and derivatives |
| CREATINE | 2.474 | 132.08084 | 29647 | 132.08 | C4H9N3O2 | Alpha amino acids and derivatives |
| Coumarin | 7.567 | 147.04438 | 17819 | 147.0441 | C9H6O2 | Coumarins and derivatives |
| Corylin | 4.794 | 321.10949 | 19541 | 321.10999 | C20H16O4 | Pyranoisoflavonoids |
| Corydaline | 9.969 | 370.19737 | 18765 | 370.20001 | C22H27NO4 | Protoberberine alkaloids and derivatives |
| Coniferyl aldehyde | 2.525 | 179.07095 | 23774 | 179.07027 | C10H10O3 | Methoxyphenols |
| Coniferaldehyde | 7.463 | 179.07017 | 3228594 | 179.0703 | C10H10O3 | Methoxyphenols |
| Codeine | 3.302 | 322.13991 | 27824 | 322.14139 | C18H21NO3 | Morphinans |
| cis-Cinnamic acid | 15.802 | 149.06015 | 18164 | 149.05971 | C9H8O2 | Cinnamic acids |
| cirsimarin | 8.72 | 477.14015 | 49154 | 477.1391 | C23H24O11 | Flavonoid O-glycosides |
| CINNAMATE | 9.266 | 149.06116 | 46536 | 149.06 | C9H8O2 | Cinnamic acids |
| Cimiracemoside D | 22.818 | 701.37339 | 7739 | 701.38 | C37H58O11 | Cucurbitacin glycosides |
| cimifugin, (s)-form, 1''-o-beta-d-glucopyranoside | 7.798 | 469.16897 | 12498 | 469.17001 | C22H28O11 | Furanochromones |
| Chrysin | 11.821 | 255.06626 | 11084 | 255.0652 | C15H10O4 | Flavones |
| Choline | 0.813 | 104.1066 | 802857 | 104.10699 | C5H14NO | Cholines |
| Chlorogenic acid | 5.994 | 355.10305 | 31489 | 355.10236 | C16H18O9 | Quinic acids and derivatives |
| Camptothecin | 18.682 | 349.10555 | 65075 | 349.10999 | C20H16N2O4 | Camptothecins |
| Calycosin-7-O-beta-D-glucoside * | 8.042 | 447.12941 | 58938 | 447.129 | C22H22O10 | Isoflavonoid O-glycosides |
| Calycosin * | 12.062 | 285.07485 | 5836970 | 285.07999 | C16H12O5 | 3'-hydroxy,4'-methoxyisoflavonoids |
| Caffeoyl lysine | 2.367 | 309.14483 | 239000 | 309.14609 | C15H20N2O5 | Hydroxycinnamic acids and derivatives |
| CAFFEIC ACID | 10.241 | 181.04983 | 111490 | 181.05 | C9H8O4 | Hydroxycinnamic acids |
| C18(Plasm)-18:1 PC | 31.555 | 284.29476 | 41111 | 284.29401 | C44H86NO7P | 1-(1Z-alkenyl),2-acyl-glycerophosphocholines |
| Butylparaben | 10.187 | 195.10211 | 40965 | 195.10159 | C11H14O3 | p-Hydroxybenzoic acid alkyl esters |
| Bullatine G | 7.225 | 380.21946 | 11374 | 380.22 | C22H31NO3 | Napelline-type diterpenoid alkaloids |
| Bufogenin | 24.45 | 407.22046 | 7830 | 407.22 | C24H32O4 | Bufanolides and derivatives |
| Bisphenol A | 21.494 | 229.12318 | 12920 | 229.1223 | C15H16O2 | Bisphenols |
| Biotin | 4.252 | 245.09224 | 66166 | 245.0954 | C10H16N2O3S | Biotin and derivatives |
| Bergamottin | 21.739 | 361.1444 | 12640 | 361.14001 | C21H22O4 | Terpene lactones |
| Berberine | 9.4 | 336.12327 | 1033775 | 336.12357 | C20H18NO4 | Protoberberine alkaloids and derivatives |
| Benzyl-2-naphthylether | 20.555 | 235.11227 | 30091 | 235.11169 | C17H14O | Naphthalenes |
| Benzoylcholine | 2.166 | 208.13328 | 44959 | 208.13266 | C12H18NO2 | Benzoic acid esters |
| Benzoic acid | 8.392 | 145.02882 | 59978 | 145.03 | C7H6O2 | Benzoic acids |
| BENZALDEHYDE | 2.286 | 107.0493 | 33005 | 107.05 | C7H6O | Benzoyl derivatives |
| Baicalin | 11.357 | 447.09355 | 43191 | 447.09271 | C21H18O11 | Flavonoid-7-O-glucuronides |
| AZELAIC ACID | 9.955 | 189.11256 | 10955 | 189.11214 | C9H16O4 | Medium-chain fatty acids |
| Azadirachtin | 5.121 | 743.25157 | 8034 | 743.24939 | C35H44O16 | Limonoids |
| Aurantiamide acetate | 19.369 | 445.21273 | 18710 | 445.21161 | C27H28N2O4 | Phenylalanine and derivatives |
| Atractylenolide I | 22.237 | 253.12278 | 16801 | 253.12 | C15H18O2 | Naphthofurans |
| Aspulvinone E | 11.031 | 297.07643 | 156474 | 297.077 | C17H12O5 | 1-hydroxy-2-unsubstituted benzenoids |
| Artemisinin | 11.512 | 283.1551 | 21585 | 283.15399 | C15H22O5 | Terpene lactones |
| Arginine | 0.631 | 175.1188 | 636244 | 175.11896 | C6H14N4O2 | L-alpha-amino acids |
| Apigenin-7-O-glucoside | 9.021 | 433.11389 | 45912 | 433.11343 | C21H20O10 | Flavonoid-7-O-glycosides |
| Apigenin 7-O-glucoside | 6.537 | 433.11436 | 9655 | 433.10999 | C21H20O10 | Flavonoid-7-O-glycosides |
| Anthranilic acid | 4.467 | 138.05521 | 28436 | 138.05548 | C7H7NO2 | Aminobenzoic acids |
| Anhydrobrazilic Acid | 8.288 | 235.06044 | 250990 | 235.0601 | C12H10O5 | Chromones |
| angoletin | 16.832 | 301.14349 | 1648525 | 301.1434 | C18H20O4 | 2'-Hydroxy-dihydrochalcones |
| ancistrocladine | 22.739 | 408.21812 | 10710 | 408.22 | C25H29NO4 | Naphthylisoquinolines |
| Amphetamine | 4.931 | 158.09647 | 86822 | 158.09399 | C9H13N | Amphetamines and derivatives |
| Amisulpride N-Oxide | 20.683 | 386.17626 | 8958 | 386.17441 | C17H27N3O5S | Aminobenzamides |
| AMINOADIPATE | 7.159 | 144.06598 | 62280 | 144.065 | C6H11NO4 | L-alpha-amino acids |
| Amfenac | 8.857 | 256.09765 | 11689 | 256.09747 | C15H13NO3 | Benzophenones |
| AM580 | 25.715 | 352.19134 | 12584 | 352.1907 | C22H25NO3 | Aromatic anilides |
| Alternariol monomethylether | 12.729 | 295.0609 | 174383 | 295.06 | C15H12O5 | Coumarins and derivatives |
| alpha-lobeline | 10.105 | 338.20785 | 19081 | 338.20999 | C22H27NO2 | Alkyl-phenylketones |
| alpha-Linolenoyl Ethanolamide | 24.463 | 322.27317 | 9215 | 322.27411 | C20H35NO2 | N-acylethanolamines |
| Alizarin | 8.843 | 241.05019 | 15890 | 241.05 | C14H8O4 | Hydroxyanthraquinones |
| albocycline | 17.012 | 309.20655 | 89285 | 309.20602 | C18H28O4 | Macrolides and analogues |
| Albiflorin * | 7.039 | 481.17085 | 1072784 | 481.1701 | C23H28O11 | Terpene glycosides |
| Alanylleucine | 1.29 | 203.13926 | 32088 | 203.14011 | C9H18N2O3 | Dipeptides |
| AKOS040738618 | 21.163 | 337.14165 | 22678 | 337.14249 | C21H20O4 | Pyranoflavonoids |
| AKOS003368487 | 5.612 | 837.36471 | 18203 | 837.35999 | C45H54N2O12 | Macrolactams |
| AKOS003368419 | 10.346 | 805.3286 | 7928 | 805.33002 | C44H56N2O12 | Macrolactams |
| Adrenosterone | 21.34 | 301.17983 | 901559 | 301.17969 | C19H24O3 | Androgens and derivatives |
| ADENOSINE 5'-MONOPHOSPHATE | 1.384 | 348.07147 | 11803 | 348.07101 | C10H14N5O7P | Purine ribonucleoside monophosphates |
| Adenosine | 1.096 | 268.10338 | 1413477 | 268.104 | C10H13N5O4 | Purine nucleosides |
| ADENINE HYDROCHLORIDE | 1.096 | 136.06212 | 203709 | 136.062 | C5H6ClN5 | 6-aminopurines |
| ADENINE | 0.863 | 136.0619 | 69592 | 136.062 | C5H5N5 | 6-aminopurines |
| Acetyl tributyl citrate | 26.874 | 403.23224 | 282378 | 403.2326 | C20H34O8 | Tetracarboxylic acids and derivatives |
| Acetaminophen / paracetamol | 5.19 | 152.07063 | 22830 | 152.0706 | C8H9NO2 | 1-hydroxy-2-unsubstituted benzenoids |
| Acenapthylene | 14.775 | 153.07083 | 17839 | 153.0699 | C12H8 | Acenaphthylenes |
| Abscisic acid | 15.842 | 265.14406 | 11843 | 265.1445 | C15H20O4 | Abscisic acids and derivatives |
| 9-methoxycarbonyldec-9-enoic acid | 13.08 | 229.14419 | 23554 | 229.14301 | C12H20O4 | Medium-chain fatty acids |
| 9-methoxy-7H-furo[3,2-g]chromen-7-one | 13.12 | 217.0498 | 69216 | 217.05 | C12H8O4 | 8-methoxypsoralens |
| 9-(2,3-dihydroxypropoxy)-9-oxononanoic acid | 9.493 | 263.14947 | 13206 | 263.1489 | C12H22O6 | Medium-chain fatty acids |
| 8phiC8SPC | 10.557 | 301.10728 | 211431 | 301.11041 | C14H20O5S | Benzenesulfonic acids and derivatives |
| 8-hydroxy-8-(3-octyloxiran-2-yl)octanoic acid | 20.555 | 337.23579 | 48332 | 337.23599 | C18H34O4 | Lineolic acids and derivatives |
| 8-hydroxy-6,7-dimethoxy-2H-chromen-2-one | 6.81 | 223.06186 | 18942 | 223.06 | C11H10O5 | Hydroxycoumarins |
| 8-hydroxy-3-(4-oxopentyl)-3,4-dihydroisochromen-1-one | 8.884 | 249.11283 | 43479 | 249.112 | C14H16O4 | 2-benzopyrans |
| 8-Deoxy-lactucin | 18.603 | 261.11301 | 27534 | 261.11401 | C15H16O4 | Gamma butyrolactones |
| 8-Benzylcanadine | 27.235 | 430.20175 | 23044 | 430.20129 | C27H27NO4 | Protoberberine alkaloids and derivatives |
| 8-(1,2-dihydroxy-3-methylbut-3-enyl)-7-methoxychromen-2-one | 10.119 | 277.10755 | 18008 | 277.10699 | C15H16O5 | Coumarins and derivatives |
| 7-Hydroxy-4-methylcoumarin | 6.756 | 177.05497 | 56328 | 177.0546 | C10H8O3 | 7-hydroxycoumarins |
| 7-hydroxy-3-(hydroxymethyl)-5-methylisochromen-1-one | 12.553 | 207.06579 | 30912 | 207.065 | C11H10O4 | Isocoumarins and derivatives |
| 7-hydroxy-3-(4-methoxyphenyl)chromen-4-one | 10.905 | 269.08069 | 1499881 | 269.08099 | C16H12O4 | 4'-O-methylisoflavones |
| 7-hydroxy-3-(3-hydroxy-4-methoxyphenyl)-6-methoxychromen-4-one | 12.499 | 315.08647 | 384622 | 315.086 | C17H14O6 | 3'-hydroxy,4'-methoxyisoflavonoids |
| 7-hydroxy-1,4a-dimethyl-9-oxo-7-propan-2-yl-2,3,4,4b,5,6,10,10a-octahydrophenanthrene-1-carboxylic acid | 16.239 | 335.22209 | 25721 | 335.22168 | C20H30O4 | Diterpenoids |
| 7-[3-[(2R,3R,4R)-3,4-dihydroxy-4-(hydroxymethyl)oxolan-2-yl]oxy-4,5-dihydroxy-6-(hydroxymethyl)oxan-2-yl]oxy-3-(4-methoxyphenyl)chromen-4-one | 10.652 | 563.17677 | 60465 | 563.17578 | C27H30O13 | Isoflavonoid O-glycosides |
| 7-[(2E)-5-hydroxy-3,7-dimethylocta-2,6-dienoxy]chromen-2-one | 19.734 | 651.29455 | 50036 | 651.29303 | C19H22O4 | Terpene lactones |
| 7-[(2E)-3,7-dimethylocta-2,6-dienoxy]chromen-2-one | 23.235 | 321.1467 | 30666 | 321.146 | C19H22O3 | Terpene lactones |
| 7,8-Dihydroxy-4-methylcoumarin | 10.611 | 193.04982 | 57470 | 193.05 | C10H8O4 | 7,8-dihydroxycoumarins |
| 7,8-dihydroxy-3-[(1E,3E)-7-hydroxy-3,5-dimethylhepta-1,3-dienyl]-7-methyl-8H-isochromen-6-one | 17.33 | 333.17031 | 124673 | 333.16965 | C19H24O5 | Azaphilones |
| 7-(Ethylamino)-4-methylcoumarin | 11.731 | 204.10204 | 9050 | 204.1019 | C12H13NO2 | Coumarins and derivatives |
| 6-Pentyl-2H-pyran-2-one | 7.319 | 167.10699 | 33285 | 167.1067 | C10H14O2 | Pyranones and derivatives |
| 6''-O-Acetylglycitin | 10.955 | 489.13949 | 1050567 | 489.14001 | C24H24O11 | Isoflavonoid O-glycosides |
| 6-Methylcoumarin | 13.377 | 161.06027 | 25051 | 161.06 | C10H8O2 | Coumarins and derivatives |
| 6-methoxy-4-methyl-2H-chromen-2-one | 10.799 | 191.07107 | 8476 | 191.07001 | C11H10O3 | Coumarins and derivatives |
| 6-isopropoxynicotinic acid | 8.341 | 204.06546 | 15930 | 204.06 | C9H11NO3 | Pyridinecarboxylic acids |
| 6-Hydroxynicotinic acid | 5.067 | 140.03453 | 50893 | 140.03474 | C6H5NO3 | Pyridinecarboxylic acids |
| 6-Hydroxy-7-methoxycoumarin | 7.987 | 193.05008 | 75129 | 193.05 | C10H8O4 | Hydroxycoumarins |
| 6-Hydroxy-5a-methyl-3,9-bis(methylene)decahydronaphtho[1,2-b]furan-2(3H)-one | 15.645 | 231.13849 | 118281 | 231.13699 | C15H20O3 | Eudesmanolides, secoeudesmanolides, and derivatives |
| 6-Hydroxy-4-methylcoumarin, 6-Hydroxy-4-methyl-2-benzopyrone | 9.629 | 177.0548 | 177320 | 177.0546 | C10H8O3 | Hydroxycoumarins |
| 6-ethyl-2,3,5-trimethyl-7H-furo[3,2-g]chromen-7-one | 13.694 | 279.10227 | 14192 | 279.10001 | C16H16O3 | Psoralens |
| 6a,7,8-trihydroxy-3-methyl-3,4,5,6,7,12a-hexahydro-2H-benzo[a]anthracene-1,12-dione | 13.975 | 329.13903 | 81448 | 329.138 | C19H20O5 | Tetralins |
| 6,8-Diprenylorobol | 20.038 | 445.16341 | 12758 | 445.16 | C25H26O6 | 6-prenylated isoflavanones |
| 6,7-dimethoxy-1-phenyl-3,4-dihydro-isoquinoline | 16.607 | 268.13417 | 8583 | 268.13 | C17H17NO2 | Dihydroisoquinolines |
| 5'-S-Methylthioadenosine | 2.924 | 298.09786 | 14283 | 298.09735 | C11H15N5O3S | 5'-deoxy-5'-thionucleosides |
| 5-OXO-L-PROLINE | 1.032 | 130.0494 | 3200474 | 130.05 | C5H7NO3 | Alpha amino acids and derivatives |
| 5-O-methylvisammioside | 24.679 | 453.16794 | 6241 | 453.17001 | C22H28O10 | Furanochromones |
| 5-methoxyindole-3-carbaldehyde | 7.039 | 176.07057 | 63812 | 176.0706 | C10H9NO2 | Indoles |
| 5-hydroxyculmorin | 18.953 | 237.18554 | 25078 | 237.1844 | C15H26O3 | Bicyclic monoterpenoids |
| 5-hydroxy-6,7-dimethoxy-2-phenylchromen-4-one | 16.331 | 299.09173 | 148979 | 299.091 | C17H14O5 | 7-O-methylated flavonoids |
| 5-hydroxy-3-[4-hydroxy-2-[(2S,3R,4S,5S,6R)-3,4,5-trihydroxy-6-(hydroxymethyl)oxan-2-yl]oxyphenyl]-7-methoxychromen-4-one | 11.974 | 463.12496 | 36199 | 463.12299 | C22H22O11 | 7-O-methylisoflavones |
| 5-hydroxy-3,6,7-trimethoxy-2-(3,4,5-trimethoxyphenyl)-4H-chromen-4-one | 18.364 | 419.13493 | 12504 | 419.133 | C21H22O9 | 7-O-methylated flavonoids |
| 5-hydroxy-3-(4-methoxyphenyl)-7-(((2S,3R,4S,5S,6R)-3,4,5-trihydroxy-6-(hydroxymethyl)tetrahydro-2H-pyran-2-yl)oxy)-4H-chromen-4-one | 10.48 | 447.12886 | 25104 | 447.13 | C22H22O10 | Isoflavonoid O-glycosides |
| 5-hydroxy-2-(4-hydroxyphenyl)-6,7-dimethoxychromen-4-one | 12.984 | 315.08727 | 11343 | 315.086 | C17H14O6 | 7-O-methylated flavonoids |
| 5-[6-(3-hydroxy-4-methoxyphenyl)-1,3,3a,4,6,6a-hexahydrofuro[3,4-c]furan-3-yl]-2-methoxyphenol | 12.553 | 341.13953 | 23468 | 341.138 | C20H22O6 | Furanoid lignans |
| 5-[(8aS)-2,5,5,8a-tetramethyl-3-oxo-4a,6,7,8-tetrahydro-4H-naphthalen-1-yl]-3-methylpentanoic acid | 16.528 | 321.243 | 41130 | 321.2424 | C20H32O3 | Diterpenoids |
| 5,8-dimethoxyquinoline-2-carbaldehyde | 10.332 | 218.08203 | 8982 | 218.08 | C12H11NO3 | Quinolines and derivatives |
| 5,7-Dimethoxy-2H-chromen-2-one | 6.345 | 207.06566 | 11300 | 207.0652 | C11H10O4 | Coumarins and derivatives |
| 5,7-dihydroxy-8-methoxy-2-phenylchromen-4-one | 16.807 | 285.07663 | 16319 | 285.07501 | C16H12O5 | 8-O-methylated flavonoids |
| 5,7-dihydroxy-6-methoxy-2-phenylchromen-4-one | 8.953 | 285.07658 | 13589 | 285.07581 | C16H12O5 | 6-O-methylated flavonoids |
| 5,7-Dihydroxy-4-methylcoumarin | 10.2 | 193.05011 | 11259 | 193.05 | C10H8O4 | 7-hydroxycoumarins |
| 5,7-dihydroxy-3-(4-methoxyphenyl)chromen-4-one | 17.682 | 285.07655 | 23018 | 285.07501 | C16H12O5 | 4'-O-methylisoflavones |
| 5,7-dihydroxy-3-(4-methoxyphenyl)-4H-chromen-4-one | 12.809 | 285.07623 | 40326 | 285.07999 | C16H12O5 | 4'-O-methylisoflavones |
| 5,7-dihydroxy-3-(4-hydroxyphenyl)-4H-chromen-4-one | 13.456 | 271.06117 | 14975 | 271.06 | C15H10O5 | Isoflavones |
| 5,7-dihydroxy-2-(4-hydroxyphenyl)-4H-chromen-4-one | 9.034 | 271.06122 | 12576 | 271.06 | C15H10O5 | Flavones |
| 4-oxododecanedioic acid | 11.018 | 245.1387 | 8536 | 245.138 | C12H20O5 | Medium-chain keto acids and derivatives |
| 4-Methylumbelliferone | 11.194 | 177.05508 | 45633 | 177.05515 | C10H8O3 | 7-hydroxycoumarins |
| 4-Methylcoumarin | 7.463 | 161.06006 | 253389 | 161.05969 | C10H8O2 | Coumarins and derivatives |
| 4-Methylbenzylidene camphor | 20.657 | 255.1746 | 163453 | 255.1743 | C18H22O | Bicyclic monoterpenoids |
| 4-Methyl-5-thiazoleethanol | 1.519 | 144.04792 | 52336 | 144.04828 | C6H9NOS | 4,5-disubstituted thiazoles |
| 4-methoxy-6-prop-2-enyl-1,3-benzodioxole | 7.635 | 193.08623 | 11766 | 193.086 | C11H12O3 | Benzodioxoles |
| 4-Hydroxyquinoline | 3.519 | 146.06012 | 31320 | 146.06004 | C9H7NO | Hydroquinolones |
| 4-hydroxymellein_130076 | 6.756 | 195.06532 | 107052 | 195.064 | C10H10O4 | 2-benzopyrans |
| 4-HYDROXYBENZALDEHYDE | 5.721 | 123.04423 | 12334 | 123.045 | C7H6O2 | Hydroxybenzaldehydes |
| 4-hydroxy-7H-furo[3,2-g]chromen-7-one | 9.629 | 203.03463 | 33164 | 203.03999 | C11H6O4 | 5-hydroxypsoralens |
| 4-hydroxy-4-(pyridin-2-yl)butan-2-one | 2.461 | 188.07034 | 2601226 | 188.07001 | C9H11NO2 | Pyridines and derivatives |
| 4-hydroxy-3-[(2E,6E)-4-hydroxy-3,7,11-trimethyldodeca-2,6,10-trienyl]benzoic acid | 27.053 | 381.20651 | 23772 | 381.20499 | C22H30O4 | Sesquiterpenoids |
| 4-hydroxy-3-(3-methylbut-2-enyl)benzoic acid | 15.645 | 207.10168 | 554250 | 207.101 | C12H14O3 | Hydroxybenzoic acid derivatives |
| 4-Formyl-antipyrine | 4.387 | 217.09733 | 855872 | 217.0972 | C12H12N2O2 | Phenylpyrazoles |
| 4-Androstene-3,17-dione | 19.475 | 287.20039 | 353363 | 287.20059 | C19H26O2 | Androgens and derivatives |
| 4-Androsten-11beta-ol-3,17-dione | 20.423 | 303.19573 | 248758 | 303.1955 | C19H26O3 | Androgens and derivatives |
| 4-Acetamidoantipyrine | 2.672 | 246.12402 | 11479 | 246.1237 | C13H15N3O2 | Phenylpyrazoles |
| 4-Acetamidoantipyrin | 8.611 | 246.12496 | 13017 | 246.1237 | C13H15N3O2 | Phenylpyrazoles |
| 4-[5-[[4-[5-[acetyl(hydroxy)amino]pentylamino]-4-oxobutanoyl]-hydroxyamino]pentylamino]-4-oxobutanoic acid | 7.132 | 461.26137 | 10538 | 461.26059 | C20H36N4O8 | N-acyl amines |
| 4-[5-(4-hydroxy-3-methoxyphenyl)-3,4-dimethyloxolan-2-yl]-2-methoxyphenol | 17.198 | 345.17072 | 22474 | 345.16965 | C20H24O5 | 7,7'-epoxylignans |
| 4-[(E)-3-[1-carboxy-2-(3,4-dihydroxyphenyl)ethoxy]-3-oxoprop-1-enyl]-2-(3,4-dihydroxyphenyl)-7-hydroxy-2,3-dihydro-1-benzofuran-3-carboxylic acid | 10.799 | 539.11973 | 49745 | 539.11841 | C27H22O12 | 2-arylbenzofuran flavonoids |
| 4',5-bisdeoxy-dothistromin | 10.799 | 341.06569 | 541286 | 341.065 | C18H12O7 | Anthraquinones |
| 4-(4-Hydroxyphenyl)butan-2-one | 8.015 | 165.09167 | 25811 | 165.091 | C10H12O2 | 1-hydroxy-2-unsubstituted benzenoids |
| 4-(2-methyl-6-oxopyran-3-yl)butanoic acid | 7.45 | 197.08153 | 146848 | 197.08099 | C10H12O4 | Pyranones and derivatives |
| 4-((3S,10R,13R,14S,17R)-3-(((2R,3R,4S,5R,6R)-3,4-dihydroxy-6-methyl-5-(((2S,3R,4S,5S,6R)-3,4,5-trihydroxy-6-(hydroxymethyl)tetrahydro-2H-pyran-2-yl)oxy)tetrahydro-2H-pyran-2-yl)oxy)-14-hydroxy-10-(hydroxymethyl)-13-methylhexadecahydro-1H-cyclopenta[a]phenanthren-17-yl)furan-2(5H)-one | 21.404 | 699.35897 | 7228 | 699.35999 | C35H54O14 | Cardenolide glycosides and derivatives |
| 3-oxo-3-[[(2R,3S,4S,5R,6S)-3,4,5-trihydroxy-6-(4-hydroxy-5-methyl-2-propan-2-ylphenoxy)oxan-2-yl]methoxy]propanoic acid | 9.021 | 432.18736 | 26561 | 432.1864 | C19H26O10 | Terpene glycosides |
| 3-METHYLHISTAMINE | 1.343 | 126.10262 | 19862 | 126.103 | C6H11N3 | 2-arylethylamines |
| 3-methoxy-4-[1-(3,4,5-trimethoxyphenyl)propan-2-yloxy]benzoic acid | 17.529 | 359.14961 | 27086 | 359.14899 | C20H24O7 | M-methoxybenzoic acids and derivatives |
| 3-methoxy-4-[1-(3,4,5-trimethoxyphenyl)propan-2-yloxy]benzaldehyde | 13.786 | 361.16533 | 38008 | 361.164 | C20H24O6 | Phenylpropanes |
| 3-Hydroxyphenylacetic acid | 2.3 | 153.05462 | 547232 | 153.0546 | C8H8O3 | 1-hydroxy-4-unsubstituted benzenoids |
| 3-HYDROXYBENZOATE | 4.589 | 139.03903 | 74811 | 139.03999 | C7H6O3 | Hydroxybenzoic acid derivatives |
| 3-Hydroxyanthranilic acid | 3.302 | 154.05038 | 28249 | 154.04987 | C7H7NO3 | Hydroxybenzoic acid derivatives |
| 3-hydroxy-3-methyl-5-oxo-5-[[(2R,3S,4S,5R,6S)-3,4,5-trihydroxy-6-(2-methyl-4-oxopyran-3-yl)oxyoxan-2-yl]methoxy]pentanoic acid | 6.332 | 433.13584 | 24867 | 433.13391 | C18H24O12 | Saccharolipids |
| 3-Hydroxy-3',4'-Dimethoxyflavone | 13.708 | 299.09182 | 35307 | 299.0914 | C17H14O5 | Flavonols |
| 3-Formylindole | 2.461 | 146.06005 | 212315 | 146.06004 | C9H7NO | Indoles |
| 3-benzyl-6-(1H-indol-3-ylmethyl)piperazine-2,5-dione | 11.77 | 334.15534 | 9059 | 334.155 | C20H19N3O2 | Alpha amino acids and derivatives |
| 3-Aminoacetophenon | 6.048 | 136.0755 | 31151 | 136.0757 | C8H9NO | Alkyl-phenylketones |
| 3-[4-methyl-1-(2-methylpropanoyl)-3-oxocyclohexyl]butanoic acid | 14.3 | 269.17511 | 73237 | 269.17471 | C15H24O4 | Menthane monoterpenoids |
| 3-[(7S,8S)-5-hydroxy-2,2,7,8-tetramethyl-6-oxo-7,8-dihydropyrano[3,2-g]chromen-10-yl]-3-phenylpropanoic acid | 20.038 | 423.18075 | 155826 | 423.17709 | C25H26O6 | Pyranochromenes |
| 3-[(2S,3R,4S,5S,6R)-4,5-dihydroxy-6-(hydroxymethyl)-3-[(2S,3R,4S,5R,6R)-3,4,5-trihydroxy-6-(hydroxymethyl)oxan-2-yl]oxyoxan-2-yl]oxy-5-hydroxy-2-(4-hydroxyphenyl)-7-methoxychromen-4-one | 5.354 | 625.17697 | 32106 | 625.17633 | C28H32O16 | Flavonoid-3-O-glycosides |
| 3,6-diphenylcyclohexane-1,2-diol | 1.871 | 291.13464 | 32215 | 291.13599 | C18H20O2 | Cyclohexanols |
| 3,6,9,12-Tetraoxatetracosan-1-ol | 26.539 | 363.31135 | 54386 | 363.31049 | C20H42O5 | Polyethylene glycols |
| 3,5,6,7,8,3',4'-Heptamethoxyflavone | 17.915 | 433.15 | 51001 | 433.14999 | C22H24O9 | 8-O-methylated flavonoids |
| 3,4-Dihydroxybenzoic acid | 2.7 | 155.03412 | 18746 | 155.034 | C7H6O4 | Hydroxybenzoic acid derivatives |
| 3',4',7-Trihydroxyflavanone | 10.01 | 273.07679 | 9309 | 273.07581 | C15H12O5 | Flavanones |
| 3,15-dihydroxyhexadecanoic acid | 13.352 | 289.23793 | 14393 | 289.237 | C16H32O4 | Long-chain fatty acids |
| 3-(benzo[d][1,3]dioxol-5-yl)-6-ethyl-7-isopropoxy-2-methyl-4H-chromen-4-one | 9.426 | 367.15037 | 38553 | 367.14999 | C22H22O5 | Isoflavones |
| 3-(3,5-dimethyl-7-oxo-7H-furo[3,2-g]chromen-6-yl)-N-(2-fluorophenyl)propanamide | 4.105 | 380.13397 | 759464 | 380.13 | C22H18FNO4 | Psoralens |
| 3-(2-Hydroxy-3,4-dimethoxyphenyl)-7-chromanol | 15.789 | 303.12292 | 411723 | 303.12299 | C17H18O5 | 4'-O-methylated isoflavonoids |
| 3-(2,4-dihydroxypentyl)-8-hydroxy-6-methoxyisochromen-1-one | 5.491 | 317.10298 | 32996 | 317.10001 | C15H18O6 | Isocoumarins and derivatives |
| 3-(1H-indol-3-ylmethyl)-2,3,6,7,8,8a-hexahydropyrrolo[1,2-a]pyrazine-1,4-dione | 8.953 | 284.14034 | 9190 | 284.13901 | C16H17N3O2 | Alpha amino acids and derivatives |
| 2-Phenylpyridine | 5.108 | 156.08126 | 8198 | 156.0808 | C11H9N | Phenylpyridines |
| 2-phenylphenate tetrahydrate | 14.775 | 171.08105 | 82911 | 171.0804 | C12H10O | Biphenyls and derivatives |
| 2-Palmitoylglycerol | 29.483 | 331.28454 | 9777 | 331.28391 | C19H38O4 | 2-monoacylglycerols |
| 2-O-Methyladenosine | 1.225 | 282.12053 | 106792 | 282.11969 | C11H15N5O4 | Purine nucleosides |
| 2-Naphthoxyacetic acid | 19.247 | 203.07082 | 9773 | 203.0703 | C12H10O3 | Phenoxyacetic acid derivatives |
| 2-methyl-4-oxo-3-phenyl-4H-chromen-7-yl acetate | 19.301 | 295.09697 | 39083 | 295.10001 | C18H14O4 | Isoflavones |
| 2-methyl-4-(pyridin-2-yl)but-3-yn-2-ol | 10.359 | 162.09171 | 28978 | 162.09 | C10H11NO | Pyridines and derivatives |
| 2-Methoxy-1-naphthaldehyde | 16.607 | 187.07554 | 21830 | 187.07539 | C12H10O2 | Naphthalenes |
| 2-Hydroxyphenylacetic acid | 9.279 | 153.05498 | 66395 | 153.0546 | C8H8O3 | 2(hydroxyphenyl)acetic acids |
| 2-hydroxy-1-phenyl-5,6,7,8-tetrahydropyrrolizin-3-one | 6.062 | 216.10269 | 11245 | 216.10201 | C13H13NO2 | Pyrrolizines |
| 2-Aminoacetophenone | 8.056 | 136.0757 | 64960 | 136.0757 | C8H9NO | Alkyl-phenylketones |
| 2-acetoxy-4-pentadecylbenzoic acid | 34.2 | 413.26694 | 11279 | 413.26599 | C24H38O4 | Acylsalicylic acids |
| 22-Hydoxy-2-hopen-1-one | 18.313 | 441.3732 | 75602 | 441.37299 | C30H48O2 | Hopanoids |
| 2-[1-(4-hydroxyphenyl)-2-propenyl]-5-methoxy-2,5-cyclohexadiene-1,4-dione | 7.595 | 271.0976 | 18074 | 271.10001 | C16H14O4 | Dalbergiones |
| 2-[(2-amino-3-methylbutanoyl)amino]-3-phenylpropanoic acid | 3.614 | 265.15471 | 60134 | 265.15399 | C14H20N2O3 | Dipeptides |
| 2,5-bis(4-hydroxy-3-methoxyphenyl)-3,4-dimethyloxolan-3-ol | 11.207 | 343.15399 | 259567 | 343.15302 | C20H24O6 | Lignan glycosides |
| 2,3-Dihydroxypyridine; CE10; GGOZGYRTNQBSSA-UHFFFAOYSA-N | 5.067 | 112.03914 | 152822 | 112.03931 | C5H5NO2 | Pyridinones |
| 2,3,5,7-tetramethoxy-9,10-dihydrophenanthrene | 16.832 | 623.26288 | 85451 | 623.26202 | C18H20O4 | Hydrophenanthrenes |
| 2,2'-(Tetradecylimino)diethanol | 17.67 | 302.30576 | 11394 | 302.30539 | C18H39NO2 | 1,2-aminoalcohols |
| 2-(6-methyl-7-oxooctyl)-2H-furan-5-one | 22.569 | 263.10718 | 21936 | 263.10501 | C13H20O3 | Butenolides |
| 2-(3,5-difluorophenyl)-N-(((1S,9aR)-octahydro-1H-quinolizin-1-yl)methyl)acetamide | 15.444 | 345.17068 | 29693 | 345.17001 | C18H24F2N2O | Quinolizines |
| 2-(3,4-dihydroxyphenyl)-7-hydroxychromen-4-one | 9.887 | 271.06054 | 19948 | 271.06 | C15H10O5 | Flavones |
| 2-(2-hydroxyphenyl)-4-phenylbenzene-1,3,5-triol | 8.367 | 336.1237 | 40905 | 336.12299 | C18H14O4 | M-terphenyls |
| 2-(2-hydroxybut-3-en-2-yl)-3a,6,6,9a-tetramethyl-2,4,5,5a,7,8,9,9b-octahydro-1H-benzo[e][1]benzofuran-4,5-diol | 15.842 | 356.28008 | 37131 | 356.27951 | C20H34O4 | Naphthofurans |
| 1-METHYLADENOSINE | 1.438 | 282.11939 | 130686 | 282.12 | C11H15N5O4 | Purine nucleosides |
| 1-isopropyl-1,2,3,4,-tetrahydro-beta-carboline-3-carboxylic acid | 8.367 | 259.1453 | 18516 | 259.14999 | C15H18N2O2 | Harmala alkaloids |
| 1H-Indole-3-propanoic acid, alpha-hydroxy-beta-methyl- | 7.595 | 220.09707 | 31494 | 220.097 | C12H13NO3 | 3-alkylindoles |
| 1h-indole-3-butanoic acid | 0.658 | 204.09806 | 44067 | 204.10001 | C12H13NO2 | 3-alkylindoles |
| 1-allyl-2-hydroxy-7,8,9,10-tetrahydroazepino[2,1-b]quinazolin-12(6H)-one | 7.73 | 271.14471 | 10247 | 271.14001 | C16H18N2O2 | Quinazolines |
| 13,17,24,25-Tetrahydroxy-16,23-epoxydammaran-3-one | 15.225 | 507.36898 | 45007 | 507.36801 | C30H50O6 | Triterpenoids |
| 11-Ketotestosterone | 14.981 | 303.19593 | 20764 | 303.1955 | C19H26O3 | Androgens and derivatives |
| 10-HYDROXYDECANOATE | 7.649 | 153.12775 | 58593 | 153.127 | C10H20O3 | Medium-chain hydroxy acids and derivatives |
| 10,11-trans-Dihydroxy-10,11-dihydrocarbamazepine | 11.449 | 271.10904 | 28934 | 271.1077 | C15H14N2O3 | Dibenzazepines |
| 1,7,8-trihydroxy-3-methyl-2,3,4,7-tetrahydro-1H-benzo[a]anthracen-12-one | 17.607 | 311.12891 | 58075 | 311.12799 | C19H18O4 | Phenanthrols |
| 1,5-Naphthalenediamine | 2.474 | 159.09147 | 58412 | 159.09171 | C10H10N2 | Naphthalenes |
| 1,5-Anhydrosorbitol | 19.734 | 165.0706 | 25892 | 165.0703 | C6H12O5 | Monosaccharides |
| 1,3-Cyclobutanedicarboxylic acid, 2,4-bis(4-hydroxyphenyl)-, monomethyl ester | 11.731 | 343.11906 | 41285 | 343.11801 | C19H18O6 | 1-hydroxy-2-unsubstituted benzenoids |
| 1-(4-Methoxyphenyl)-1-pentene-3-one | 19.696 | 191.10703 | 526560 | 191.1067 | C12H14O2 | Anisoles |
| 1-(3-carboxypropyl)-3,7-dimethylxanthine | 5.34 | 267.11279 | 7237 | 267.1088 | C11H14N4O4 | Xanthines |
| 1-(2-hydroxy-4,6-dimethoxyphenyl)ethanone | 5.477 | 197.08141 | 25689 | 197.08 | C10H12O4 | Alkyl-phenylketones |
| 1-(2-(1H-indol-3-yl)ethyl)urea | 7.476 | 226.09852 | 13931 | 226.10001 | C11H13N3O | 3-alkylindoles |
| [D-Asp3,E-Dhb7]-Microcystin-RR | 8.482 | 1024.54929 | 13767 | 1024.55737 | C48H73N13O12 | Hybrid peptides |
| [3,4,5-trihydroxy-6-(hydroxymethyl)oxan-2-yl] 9-(hydroxymethyl)-2,2,6a,6b,9,12a-hexamethyl-10-[3,4,5-trihydroxy-6-[(3,4,5-trihydroxy-6-methyloxan-2-yl)oxymethyl]oxan-2-yl]oxy-1,3,4,5,6,6a,7,8,8a,10,11,12,13,14b-tetradecahydropicene-4a-carboxylate | 18.339 | 965.51057 | 8766 | 965.50562 | C48H78O18 | Triterpene saponins |
| [(1S,3R,5R,6S,8S)-3-{[(2S,3R,4S,5S,6R)-6-[(benzoyloxy)methyl]-3,4,5-trihydroxyoxan-2-yl]oxy}-6-hydroxy-8-methyl-9,10-dioxatetracyclo[4.3.1.0?,?.0?,?]decan-2-yl]methyl benzoate | 13.365 | 602.22379 | 863088 | 602.22302 | C30H32O12 | Terpene glycosides |
| [(1S,2S,3R,5R,6S,8S)-6-hydroxy-8-methyl-3-{[(2S,3R,4S,5S,6R)-3,4,5-trihydroxy-6-(hydroxymethyl)oxan-2-yl]oxy}-9,10-dioxatetracyclo[4.3.1.0?,?.0?,?]decan-2-yl]methyl benzoate | 9.089 | 498.19804 | 42109 | 498.19699 | C23H28O11 | Terpene glycosides |
| (Z)-9,12,13-trihydroxyoctadec-15-enoic acid | 15.211 | 353.23075 | 104799 | 353.2291 | C18H34O5 | Long-chain fatty acids |
| (Z)-7-((butyl(methyl)ammonio)methyl)-2-(2,5-dimethoxybenzylidene)-3-oxo-2,3-dihydrobenzofuran-6-olate | 13.989 | 398.19702 | 31162 | 398.20001 | C23H27NO5 | Aurone flavonoids |
| (Z)-6-methoxybenzofuran-3(2H)-one oxime | 4.078 | 180.06625 | 24187 | 180.07001 | C9H9NO3 | Coumarans |
| (Z)-5,8,11-trihydroxyoctadec-9-enoic acid | 15.225 | 348.27523 | 294451 | 348.27341 | C18H34O5 | Long-chain fatty acids |
| (Z)-2-(4-(methoxycarbonyl)benzylidene)-7-(morpholino-4-iummethyl)-3-oxo-2,3-dihydrobenzofuran-6-olate | 6.414 | 396.14442 | 19350 | 396.14001 | C22H21NO6 | Aurone flavonoids |
| (S)-4-((7-acetamido-1,2,3-trimethoxy-9-oxo-5,6,7,9-tetrahydrobenzo[a]heptalen-10-yl)amino)-N-(3-(methylthio)phenyl)butanamide | 6.226 | 592.25114 | 16324 | 592.25 | C32H37N3O6S | Gamma amino acids and derivatives |
| (S)-3-(4-(2-(2-(hydroxymethyl)pyrrolidin-1-yl)-2-oxoethoxy)phenyl)-8-methoxy-2H-chromen-2-one | 2.34 | 410.15604 | 52903 | 410.16 | C23H23NO6 | Isoflav-3-enones |
| (S)-2-(((S)-7-acetamido-1,2,3-trimethoxy-9-oxo-5,6,7,9-tetrahydrobenzo[a]heptalen-10-yl)amino)-4-(methylthio)-N-(2-(2-oxopyrrolidin-1-yl)ethyl)butanamide | 19.079 | 627.29286 | 11515 | 627.28998 | C32H42N4O7S | Methionine and derivatives |
| (E)-N-[2-hydroxy-2-(4-methoxyphenyl)ethyl]-3-phenylprop-2-enamide | 4.958 | 320.12442 | 231844 | 320.12601 | C18H19NO3 | Cinnamic acid amides |
| (E)-7-(4-methoxy-5-methyl-6-oxopyran-2-yl)-5-methyloct-6-enoic acid | 13.721 | 295.15454 | 231795 | 295.15399 | C16H22O5 | Medium-chain fatty acids |
| (E)-5-hydroxy-1,7-diphenylhept-1-en-3-one | 25.591 | 583.28267 | 16153 | 583.28101 | C19H20O2 | Linear diarylheptanoids |
| (E)-4-[(1R,2S,3S,4R,8aS)-2,3,4-trihydroxy-2,5,5,8a-tetramethyl-3,4,4a,6,7,8-hexahydro-1H-naphthalen-1-yl]but-3-en-2-one | 18.544 | 311.22245 | 83751 | 311.22168 | C18H30O4 | Sesquiterpenoids |
| (E)-1-(2,4-dihydroxyphenyl)-3-(4-hydroxyphenyl)prop-2-en-1-one | 8.571 | 257.08228 | 10610 | 257.08099 | C15H12O4 | 2'-Hydroxychalcones |
| (9Z,12E)-15,16-dihydroxyoctadeca-9,12-dienoic acid | 18.953 | 313.23789 | 539774 | 313.23734 | C18H32O4 | Lineolic acids and derivatives |
| (7R,8S)-7,8-dihydroxy-3,7-dimethyl-6-oxo-8H-isochromene-5-carbaldehyde | 10.214 | 237.07597 | 12292 | 237.075 | C12H12O5 | Azaphilones |
| (7,8-dimethoxy-2,2,3-trimethyl-2,3-dihydro-1H-benzo[d]azepin-4-yl)(3-hydroxy-2H-benzo[b][1,4]oxazin-7-yl)methanone | 14.813 | 423.19319 | 16300 | 423.19 | C24H26N2O5 | Benzazepines |
| (7,8-dimethoxy-2,2,3-trimethyl-2,3-dihydro-1H-benzo[d]azepin-4-yl)(3,4-dimethoxyphenyl)methanone | 21.482 | 412.21302 | 14584 | 412.20999 | C24H29NO5 | Benzazepines |
| (6E,10Z,14E)-6,14-dimethyl-3-methylidene-2-oxo-3a,4,5,8,9,12,13,15a-octahydrocyclotetradeca[b]furan-10-carboxylic acid | 23.6 | 331.191 | 12688 | 331.18909 | C20H26O4 | Cembranolides |
| (5xi,9xi)-Beyer-15-en-18-ol | 21.456 | 271.24271 | 13927 | 271.241 | C20H32O | Diterpenoids |
| (5R)-5-hydroxy-1-(4-hydroxy-3-methoxyphenyl)tetradecan-3-one | 14.3 | 351.25326 | 52042 | 351.25296 | C21H34O4 | Gingerols |
| (5beta,8alpha,10alpha,16xi)-16,17-Dihydroxykaur-9(11)-en-18-oic acid | 24.873 | 317.2109 | 178193 | 317.211 | C20H30O4 | Kaurane diterpenoids |
| (5-benzoyloxy-4,6-dihydroxy-3-methoxycyclohexen-1-yl)methyl benzoate | 14.328 | 421.1296 | 11427 | 421.12601 | C22H22O7 | Benzoic acid esters |
| (4S,5Z,6S)-4-(2-methoxy-2-oxoethyl)-5-[2-[(E)-3-phenylprop-2-enoyl]oxyethylidene]-6-[(2S,3R,4S,5S,6R)-3,4,5-trihydroxy-6-(hydroxymethyl)oxan-2-yl]oxy-4H-pyran-3-carboxylic acid | 17.915 | 329.13919 | 16288 | 329.1384 | C19H20O5 | Angular pyranocoumarins |
| (4-chlorophenyl)(7,8-dimethoxy-3-methyl-2,3-dihydro-1H-benzo[d]azepin-4-yl)methanone | 0.762 | 380.09527 | 211479 | 380.10001 | C20H20ClNO3 | Benzazepines |
| (3Z,5E)-4-hydroxy-6-(4-hydroxyphenyl)hexa-3,5-dien-2-one | 16.607 | 205.0859 | 350764 | 205.08592 | C12H12O3 | Styrenes |
| (3S,4R)-7-hydroxy-3-[(3S,4R)-7-hydroxy-4-(4-hydroxyphenyl)-2-oxo-5-[(2S,3R,4S,5S,6R)-3,4,5-trihydroxy-6-(hydroxymethyl)oxan-2-yl]oxy-3,4-dihydrochromen-3-yl]-4-(4-hydroxyphenyl)-5-[(2S,3R,4S,5S,6R)-3,4,5-trihydroxy-6-(hydroxymethyl)oxan-2-yl]oxy-3,4-dihydrochromen-2-one | 0.775 | 867.23911 | 60596 | 867.23059 | C42H42O20 | Coumarin glycosides |
| (3beta,5xi,9xi,13alpha,17alpha,18xi)-3-Hydroxy-13,28-epoxyurs-11-en-28-one | 23.952 | 437.34244 | 41144 | 437.341 | C30H46O3 | Triterpenoids |
| (3,5,14-trihydroxy-13-methyl-17-(5-oxo-2,5-dihydrofuran-3-yl)hexadecahydro-1H-cyclopenta[a]phenanthren-10-yl)methyl nitrate | 12.984 | 452.2188 | 13989 | 452.22 | C23H33NO8 | Cardenolides and derivatives |
| (2Z)-4,6-dihydroxy-2-[(4-hydroxy-3,5-dimethoxyphenyl)methylidene]-1-benzofuran-3-one | 11.948 | 331.08241 | 12064 | 331.0813 | C17H14O7 | Aurone flavonoids |
| (2Z)-4,6-dihydroxy-2-[(3-hydroxy-4-methoxyphenyl)methylidene]-1-benzofuran-3-one | 14.098 | 301.07063 | 128386 | 301.07065 | C16H12O6 | Aurone flavonoids |
| (2S,3S,4S,5R,6R)-6-[[(3S,4S,6aR,6bS,8aR,9R,12aS,14bR)-9-hydroxy-4-(hydroxymethyl)-4,6a,6b,8a,11,11,14b-heptamethyl-1,2,3,4a,5,6,7,8,9,10,12,12a,14,14a-tetradecahydropicen-3-yl]oxy]-5-[(2S,3R,4S,5R,6R)-4,5-dihydroxy-6-(hydroxymethyl)-3-[(2S,3R,4R,5R,6S)-3,4,5-trihydroxy-6-methyloxan-2-yl]oxyoxan-2-yl]oxy-3,4-dihydroxyoxane-2-carboxylic acid | 18.339 | 943.52536 | 724405 | 943.52612 | C48H78O18 | Triterpene saponins |
| (2R,3S,4S,5R,6S)-2-(hydroxymethyl)-6-[4-(hydroxymethyl)-1-propan-2-ylcyclohex-3-en-1-yl]oxyoxane-3,4,5-triol | 8.097 | 350.21732 | 13640 | 350.21732 | C16H28O7 | Terpene glycosides |
| (2R,3S,4S,5R,6R)-2-[[(2R,3R,4R,5S)-3,4-dihydroxy-5-(hydroxymethyl)oxolan-2-yl]oxymethyl]-6-[(2E)-3,7-dimethylocta-2,6-dienoxy]oxane-3,4,5-triol | 11.461 | 466.26475 | 15256 | 466.26465 | C21H36O10 | Terpene glycosides |
| (2R,3S,4S,5R,6R)-2-[[(2R,3R,4R)-3,4-dihydroxy-4-(hydroxymethyl)oxolan-2-yl]oxymethyl]-6-[(2E)-3,7-dimethylocta-2,6-dienoxy]oxane-3,4,5-triol | 12.257 | 466.26538 | 68028 | 466.26321 | C21H36O10 | Terpene glycosides |
| (2R,3R,4S,5S,6R)-2-[(2E)-4-hydroxy-3,7-dimethylocta-2,6-dienoxy]-6-(hydroxymethyl)oxane-3,4,5-triol | 8.417 | 350.21824 | 14733 | 350.21732 | C16H28O7 | Terpene glycosides |
| (2R,3R,4S,5R)-2-(6-((E)-4-hydroxy-3-methylbut-2-enylamino)-9H-purin-9-yl)-5-(hydroxymethyl)tetrahydrofuran-3,4-diol | 17.279 | 352.16495 | 9487 | 352.16 | C15H21N5O5 | Purine nucleosides |
| (2R,3R)-3,7-dihydroxy-6-methoxy-2-phenyl-2,3-dihydrochromen-4-one | 12.782 | 287.09223 | 34562 | 287.0914 | C16H14O5 | 6-O-methylated flavonoids |
| (2R,3R)-3,5-dihydroxy-2-(4-hydroxyphenyl)-7-methoxy-2,3-dihydrochromen-4-one | 10.091 | 303.08705 | 21184 | 303.086 | C16H14O6 | 7-O-methylated flavonoids |
| (2R)-5,8-dihydroxy-2-(2-hydroxyphenyl)-7-methoxy-2,3-dihydrochromen-4-one | 13.76 | 285.07651 | 30087 | 285.07501 | C16H14O6 | 7-O-methylated flavonoids |
| (2E,8E)-9-(1,3-benzodioxol-5-yl)-1-piperidin-1-ylnona-2,8-dien-1-one | 21.391 | 364.19186 | 18432 | 364.18799 | C21H27NO3 | Benzodioxoles |
| (2E,4E)-12-hydroxy-13-(hydroxymethyl)-3,5,7-trimethyltetradeca-2,4-dienedioic acid | 18.044 | 307.1913 | 22601 | 307.19 | C18H30O6 | Long-chain fatty acids |
| (2E)-1-(2,4-dihydroxyphenyl)-3-(4-hydroxyphenyl)-2-propen-1-one | 11.384 | 257.0812 | 42833 | 257.07999 | C15H12O4 | 2'-Hydroxychalcones |
| (2alpha,3beta,5xi,9xi,18xi)-2,3-Dihydroxyolean-12-en-28-oic acid | 24.692 | 473.36289 | 26645 | 473.36301 | C30H48O4 | Triterpenoids |
| (2-{[3-(hexadecanoyloxy)-2-hydroxypropyl phosphonato]oxy}ethyl)trimethylazanium | 23.404 | 496.34058 | 9424 | 496.34 | C24H50NO7P | 1-acyl-sn-glycero-3-phosphocholines |
| (1R,5S)-3-(1-oxo-2-(pyridin-4-ylmethyl)-1,2-dihydroisoquinoline-4-carbonyl)-3,4,5,6-tetrahydro-1H-1,5-methanopyrido[1,2-a][1,5]diazocin-8(2H)-one | 20.49 | 453.1895 | 12703 | 453.19 | C27H24N4O3 | Cytisine and derivatives |
| (1R,3aS,5aR,5bR,11aR,13aR)-7-hydroxy-5a,5b,8,8,11a-pentamethyl-9-oxo-1-prop-1-en-2-yl-2,3,4,5,6,7,7a,10,11,11b,12,13,13a,13b-tetradecahydro-1H-cyclopenta[a]chrysene-3a-carboxylic acid | 20.181 | 471.34739 | 78051 | 471.34686 | C30H46O4 | Triterpenoids |
| (1R,2R,5S,8R,14R,15R,16S)-16-hydroxy-1,2,14,17,17-pentamethyl-8-(prop-1-en-2-yl)pentacyclo[11.7.0.0?,??.0?,?.0??,??]icosane-5,15-dicarboxylic acid | 20.862 | 487.34191 | 23555 | 487.34079 | C30H46O5 | Triterpenoids |
| (1R,2R,5R,5'S,6S,8aS)-6-(acetyloxy)-2,5,8a-trimethyl-5''-oxo-octahydro-2H-dispiro[naphthalene-1,2':5',3''-bis(oxolane)]-5-ylmethyl acetate | 13.202 | 437.25545 | 15347 | 437.25 | C24H36O7 | Diterpene lactones |
| (1R,2R,4aS,6aS,6bR,10S,12aR,14bS)-1,8,10-trihydroxy-1,2,6a,6b,9,9,12a-heptamethyl-2,3,4,5,6,6a,7,8,8a,10,11,12,13,14b-tetradecahydropicene-4a-carboxylic acid | 20.181 | 489.35832 | 9615 | 489.35751 | C30H48O5 | Triterpenoids |
| (1E,4E)-1,5-bis(4-methoxyphenyl)penta-1,4-dien-3-one | 25.083 | 611.24106 | 174434 | 611.24103 | C19H18O3 | Anisoles |
| (16alpha,17beta)-estra-1,3,5(10)-triene-3,16,17-triol | 18.058 | 289.18037 | 25190 | 289.17999 | C18H24O3 | Estrogens and derivatives |
| (10E,15Z)-9,12,13-trihydroxyoctadeca-10,15-dienoic acid | 14.232 | 346.25951 | 56694 | 346.25751 | C18H32O5 | Lineolic acids and derivatives |
| (+/-)-alpha-lipoic acid amide | 2.394 | 228.05018 | 17860 | 228.05 | C8H15NOS2 | Lipoamides |
| (+)-Costunolide | 13.297 | 233.1542 | 28176 | 233.15359 | C15H20O2 | Germacranolides and derivatives |
| (+)Catechin | 4.279 | 291.08687 | 29701 | 291.09 | C15H14O6 | Catechins |
| (-)-Riboflavin | 6.81 | 377.1459 | 196168 | 377.14557 | C17H20N4O6 | Flavins |
| (-)-Nuciferine | 14.838 | 296.16532 | 36185 | 296.16449 | C19H21NO2 | Aporphines |

## **2.4 Supplementary Table 4. LC-MS/MS parameters for untargeted metabolomics analysis**

***Liquid Chromatography Conditions***

| **Ion Mode** | **Instrument & Column** | **Mobile Phases (A/B)** | **Gradient Program** | **Flow Rate** | **Column Temp** |
| --- | --- | --- | --- | --- | --- |
| Positive (ESI+) | Vanquish UHPLC System (Thermo Fisher) + ACQUITY UPLC® HSS T3 (150×2.1 mm, 1.8 µm) | A2: 0.1% formic acid in water B2: 0.1% formic acid in acetonitrile | 0-1 min: 2% B2 1-9 min: 2%→50% B2 9-12 min: 50%→98% B2 12-13.5 min: 98% B2 13.5-14 min: 98%→2% B2 14-20 min: 2% B2 | 0.25 mL/min | 40 °C |
| Negative (ESI-) | Same as above | A3: 5 mM ammonium formate B3: Acetonitrile | 0-1 min: 2% B3 1-9 min: 2%→50% B3 9-12 min: 50%→98% B3 12-13.5 min: 98% B3 13.5-14 min: 98%→2% B3 14-17 min: 2% B3 | 0.25 mL/min | 40 °C |

***Mass Spectrometry Conditions***

| **Instrument** | **Ion Source** | **Spray Voltage (±)** | **Capillary Temp** | | **MS1 Range / Resolving Power** | **MS/MS Resolving Power** | **Collision Energy & Scan Settings** |
| --- | --- | --- | --- | --- | --- | --- | --- |
| Orbitrap Exploris 120 (Thermo Fisher) | ESI (Full MS-ddMS2) | +3.50 kV / -2.50 kV | | 325 °C | m/z 100-1000 / 60000 FWHM | 15000 FWHM | Sheath gas: 30 arb Aux gas: 10 arb CE: 30% 4 ddMS2 scans/cycle Dynamic exclusion: auto |

## **2.5 Supplementary Table 5. Detection of QBHF constituents in serum from QBHF-treated rats (Negative-ion mode).**

| **Classification** | **Subcategory** | **Retention time**  **(min)** | **Metabolite** | **Type** |
| --- | --- | --- | --- | --- |
| Organic acids | Dicarboxylic acid | 0.92 | MALATE | neg |
|  |  | 0.96 | MS2: Aconitic Acid | neg |
|  |  | 1.21 | Citric acid | neg |
|  |  | 1.37 | Succinic acid | neg |
|  |  | 10.61 | Azelaic acid | neg |
|  |  | 14.39 | Undecanedioic acid | neg |
|  |  | 16.16 | Dodecanedioic acid | neg |
|  |  | 17.84 | Tridecanedioic acid | neg |
|  | Aromatic acid | 0.95 | MS2: 2-Furoic acid | neg |
|  |  | 1.69 | Gallic acid | neg |
|  | Short-chain fatty acids | 1.36 | Meglutol | neg |
|  |  | 4.04 | 3-Hydroxyvaleric acid | neg |
|  | Others | 0.79 | Gluconic acid | neg |
|  |  | 1.06 | MS2: DL-Lactic acid | neg |
|  |  | 1.38 | MS2: Citramalate | neg |
| Phenylbutazone | Flavonoids | 9.11 | MS2: Biochanin A * | neg |
|  | Phenolic acids | 7.87 | 4-Hydroxyacetophenone | neg |
|  |  | 10.03 | Salicylic acid | neg |
|  | Phenylpropionic acid derivatives | 5.73 | MS2: 3-[4-methoxy-2-[(2S,3R,4S,5S,6R)-3,4,5-trihydroxy-6-(hydroxymethyl)oxan-2-yl]oxyphenyl]propanoic acid | neg |
| Fatty acids and their oxidation derivatives | Oxidized fatty acids | 13.73 | MS2: FA 18:2+4O | neg |
|  |  | 13.85 | MS2: FA 9:1+1O | neg |
|  |  | 14.02 | MS2: (10E,15Z)-9,12,13-trihydroxyoctadeca-10,15-dienoic acid | neg |
|  |  | 14.91 | (10E,15Z)-9,12,13-trihydroxyoctadeca-10,15-dienoic acid | neg |
|  |  | 18.81 | MS2: FA 18:1+2O; PlaSMA ID-662 | neg |
|  |  | 20.51 | MS2: FA 18:1+2O | neg |
| Amino acids and their metabolites | Amino acids | 4.31 | Tryptophan | neg |
|  | Amino acid derivatives | 1.07 | MS2: N-Acetylglutamic acid | neg |
|  |  | 1.27 | N-Acetylglutamic acid | neg |
|  |  | 1.41 | MS2: C6H9NO3; PlaSMA ID-80 | neg |
|  |  | 1.43 | MS2: 3-oxo-C4-homoserine lactone | neg |
| Nucleotide/Vitamin-Related | Vitamin | 1.22 | MS2: 6-Hydroxynicotinic acid | neg |
|  |  | 1.56 | 4-Pyridoxic acid | neg |
|  |  | 3.07 | Pantothenate | neg |
| Carbohydrate derivatives | / | 12.94 | 2-[2-[(Z)-pent-2-enyl]-3-[3,4,5-trihydroxy-6-(hydroxymethyl)oxan-2-yl]oxycyclopentyl]acetic acid | neg |

## **2.6 Supplementary Table 6. Detection of QBHF constituents in serum from QBHF-treated rats (Positive-ion mode).**

| **Classification** | **Subcategory** | **Retention time（min）** | **Metabolite** | **Type** |
| --- | --- | --- | --- | --- |
| Phenylpropanoids & Phenolic acids | Flavonoids | 9.89 | 2-(3,4-dihydroxyphenyl)-7-hydroxychromen-4-one | pos |
|  |  | 11.38 | (2E)-1-(2,4-dihydroxyphenyl)-3-(4-hydroxyphenyl)-2-propen-1-one | pos |
|  |  | 12.06 | Calycosin * | pos |
|  |  | 13.71 | 3-Hydroxy-3',4'-Dimethoxyflavone | pos |
|  |  | 15.51 | Flavonol base + 2MeO | pos |
|  |  | 16.33 | 5-hydroxy-6,7-dimethoxy-2-phenylchromen-4-one | pos |
|  |  | 16.83 | Angoletin | pos |
|  | Coumarins | 6.16 | Mellein | pos |
|  |  | 6.62 | Paeonol * | pos |
|  |  | 10.61 | 7,8-Dihydroxy-4-methylcoumarin | pos |
|  |  | 13.81 | Isopropylidenylacetyl-marmesin | pos |
|  | Phenolic acids | 2.30 | 3-Hydroxyphenylacetic acid | pos |
|  |  | 2.53 | Coniferyl aldehyde | pos |
|  |  | 4.59 | 3-HYDROXYBENZOATE | pos |
|  |  | 5.72 | 4-HYDROXYBENZALDEHYDE | pos |
|  |  | 11.73 | 1,3-Cyclobutanedicarboxylic acid, 2,4-bis(4-hydroxyphenyl)-, monomethyl ester | pos |
|  |  | 13.77 | trans-Ferulic acid | pos |
|  |  | 15.65 | 4-hydroxy-3-(3-methylbut-2-enyl)benzoic acid | pos |
|  | Cinnamic acid derivatives | 7.46 | trans-Cinnamaldehyde | pos |
|  |  | 9.27 | CINNAMATE | pos |
|  |  | 15.80 | cis-Cinnamic acid | pos |
|  | Benzaldehyde/Phenylaldehyde Derivatives | 13.79 | 3-methoxy-4-[1-(3,4,5-trimethoxyphenyl)propan-2-yloxy]benzaldehyde | pos |
|  |  | 14.30 | 3-[4-methyl-1-(2-methylpropanoyl)-3-oxocyclohexyl] butanoic acid | pos |
|  |  | 15.99 | Phenylacetaldehyde | pos |
|  |  | 16.61 | (3Z,5E)-4-hydroxy-6-(4-hydroxyphenyl)hexa-3,5-dien-2-one | pos |
|  | Lignans | 20.58 | Guaiacin | pos |
| Alkaloids | Isoquinoline derivatives | 5.50 | Tetrahydroalstonine | pos |
|  |  | 14.84 | (-)-Nuciferine |  |
|  | Pyridine/Pyrrole | 0.86 | TRIGONELLINE | pos |
|  |  | 2.44 | Gentiannine | pos |
|  | Others | 0.76 | (4-chlorophenyl) (7,8-dimethoxy-3-methyl-2,3-dihydro-1H-benzo[d]azepin-4-yl)methanone | pos |
|  |  | 1.82 | synephrine | pos |
|  |  | 2.46 | 4-hydroxy-4-(pyridin-2-yl) butan-2-one | pos |
|  |  | 5.24 | Pyrrolo[1,2-a]pyrazine-1,4-dione, hexahydro-3-(1-methylethyl)- | pos |
|  |  | 11.99 | Indirubin | pos |
|  |  | 15.44 | 2-(3,5-difluorophenyl)-N-(((1S,9aR)-octahydro-1H-quinolizin-1-yl)methyl)acetamide | pos |
| Terpenes | Sesquiterpene | 1.83 | desmotroposantonin | pos |
|  |  | 13.30 | (+)-Costunolide | pos |
|  |  | 15.84 | 2-(2-hydroxybut-3-en-2-yl)-3a,6,6,9a-tetramethyl-2,4,5,5a,7,8,9,9b-octahydro-1H-benzo[e][1]benzofuran-4,5-diol | pos |
|  |  | 18.95 | 5-hydroxyculmorin | pos |
|  | Triterpenes & Steroids | 14.25 | Nandrolone | pos |
|  |  | 15.23 | 13,17,24,25-Tetrahydroxy-16,23-epoxydammaran-3-one | pos |
|  |  | 16.53 | 5-[(8aS)-2,5,5,8a-tetramethyl-3-oxo-4a,6,7,8-tetrahydro-4H-naphthalen-1-yl]-3-methylpentanoic acid | pos |
|  |  | 18.06 | (16alpha,17beta)-estra-1,3,5(10)-triene-3,16,17-triol | pos |
|  |  | 18.54 | (E)-4-[(1R,2S,3S,4R,8aS)-2,3,4-trihydroxy-2,5,5,8a-tetramethyl-3,4,4a,6,7,8-hexahydro-1H-naphthalen-1-yl]but-3-en-2-one | pos |
|  | Monoterpene | 10.28 | Isophorone | pos |
|  |  | 15.35 | myrcene | pos |
|  | Diterpenoid quinones | 13.98 | 6a,7,8-trihydroxy-3-methyl-3,4,5,6,7,12a-hexahydro-2H-benzo[a]anthracene-1,12-dione | pos |
| Fatty acids and related lipids | Oxidated fatty acids | 15.21 | (Z)-9,12,13-trihydroxyoctadec-15-enoic acid | pos |
|  |  | 15.84 | FA 18:4+1O | pos |
|  |  | 18.95 | (9Z,12E)-15,16-dihydroxyoctadeca-9,12-dienoic acid | pos |
|  | Fatty acyl | 14.30 | (5R)-5-hydroxy-1-(4-hydroxy-3-methoxyphenyl)tetradecan-3-one | pos |
|  |  | 13.93 | Valerophenone | pos |
|  | Dicarboxylic acid | 11.02 | 4-oxododecanedioic acid | pos |
| Amino acids, peptides, and their metabolites | Amino acids | 0.63 | Arginine | pos |
|  |  | 0.89 | Proline | pos |
|  |  | 1.10 | Tyrosine | pos |
|  |  | 1.11 | Isoleucine | pos |
|  |  | 1.38 | L-(-)-Phenylalanine | pos |
|  |  | 1.56 | Phenylalanine | pos |
|  |  | 2.25 | L-Arginine | pos |
|  |  | 2.29 | N2,N2-Dimethylguanosine | pos |
|  |  | 2.46 | Tryptophan | pos |
|  |  | 2.92 | 5'-S-Methylthioadenosine | pos |
|  |  | 4.88 | L-Tryptophan | pos |
|  | Dipeptide | 1.23 | Isoleucylserine | pos |
|  |  | 1.29 | Alanylleucine | pos |
|  |  | 2.30 | H-Pro-Hyp-OH | pos |
|  |  | 3.34 | Isoleucylaspartate | pos |
|  |  | 3.94 | gamma-Glutamylleucine | pos |
|  |  | 4.84 | Glutamylphenylalanine | pos |
|  | Amino acid derivatives | 0.66 | 1h-indole-3-butanoic acid | pos |
|  |  | 1.03 | 5-OXO-L-Proline | pos |
|  |  | 2.46 | 3-Formylindole | pos |
|  |  | 2.55 | N-Acetylproline | pos |
|  |  | 3.00 | N-Carbobenzyloxyleucine | pos |
|  |  | 3.75 | N-Benzyloxycarbonylglycine | pos |
|  |  | 7.66 | Indole-3-Acetate | pos |
| Nucleotides and Their Metabolites | Purine & Pyrimidine | 0.86 | Adenine |  |
|  |  | 1.10 | Adenosine | pos |
|  |  | 1.12 | Guanine | pos |
|  |  | 2.03 | Guanosine cyclic monophosphate | pos |
| Carbohydrates and their derivatives | Sugar & Sugar Alcohols | 0.78 | Stachyose | pos |
|  |  | 0.78 | Melezitose | pos |
|  |  | 0.80 | Psicose | pos |
|  |  | 2.29 | D-(+)-Glucosamine | pos |
|  |  | 19.73 | 1,5-Anhydrosorbitol | pos |
|  | Sugar-amino acid conjugates | 1.16 | Pentose + Proline | pos |
|  |  | 1.19 | N-Fructosyl isoleucine | pos |
|  |  | 1.81 | N-Fructosyl phenylalanine | pos |
| Others | / | 2.47 | Creatine | pos |
|  |  | 0.81 | Choline | pos |
|  |  | 1.01 | Nicotinamide | pos |
|  |  | 1.10 | Nicotinate | pos |
|  |  | 2.41 | Pantothenate | pos |

## **2.7 Supplementary Table 7. Primer sequences**

| Gene | Forward | Reverse |
| --- | --- | --- |
| Mmu-FTO | TTCATGCTGGATGACCTCAATG | GCCAACTGACAGCGTTCTAAG |
| Mmu-*β-actin* | GTAGCCATCCAGGCTGTGTT | ATGTCACGCACGATTTCCCT |
| hum-FTO | ACTTGGCTCCCTTATCTGACC | TGTGCAGTGTGAGAAAGGCTT |

**3. Supplementary figures**

## **3.1 Supplementary figure 1**

**Fig. S1** Characterization of QBHF serum effects, FTO inhibitor selection, and chemical profiling of QBHF components. (A) Cell viability of AC16 cardiomyocytes treated with increasing volumes of QBHF-containing serum derived from rats administered low (A), medium (B), and high (C) doses of QBHF-serum. (D) Selection of FB23-2 working concentration in AC16 cardiomyocytes. (E) BPI plot in negative/positive ion mode. (F) Total ion flow diagram in negative/positive ion mode.

## **3.2 Supplementary figure 2**

**Fig. S2** Immunohistochemical analysis of Collagen Type I protein expression in mouse cardiac tissue.

(A-**B)** IHC **and quantification of** Collagen Type I protein levels in mouse cardiac tissue (n = 3). **Scale bar = 20 µm**.

## **3.3 Supplementary figure 3**

**Fig. S3** Original, uncropped Western blot images correspond to the main figures. (A-D) Blots correspond to the cropped bands shown in Figure 9C. (E) Blots correspond to the cropped bands shown in Figure 2J.

**Reference**

[1] Sun, Y., et al. *Angelica sinensis* polysaccharide as potential protectants against recurrent spontaneous abortion: focus on autophagy regulation. Front Med (Lausanne). 2025; 12:1522503. https://doi.org/10.3389/fmed.2025.1522503.

[2] Zhong, Y., et al. *Bacillus licheniformis* alleviates clostridium perfringens-induced intestinal injury in mice model by modulating inflammation, apoptosis, and cecal microbial-metabolic responses. Animals (Basel). 2025; 15, 1409. https://doi.org/10.3390/ani15101409.
